# Supplementary material for: Effectiveness of conditional cash transfers (Afya credits incentive) to retain women in the continuum of care during pregnancy, birth and the postnatal period in Kenya: a cluster-randomised trial
Source: BMJ Open. 2022 Jan 6;12(1):e055921. doi: 10.1136/bmjopen-2021-055921 (PMC8739676; doi:10.1136/bmjopen-2021-055921)
Supplement: Supplementary data [file bmjopen-2021-055921supp001.pdf]

Supplementary material

Appendix S1 – Differences from published protocol in data collection and analysis

This summary of differences between the trial protocol and the data collection and analysis that were conducted is closely based on that in the statistical analysis plan (SAP) for the study, which was written prior to unblinding and analysis of data by the trial statistician.

The published Protocol for the study stated that the primary outcomes for the study would be derived by combining data from manual extraction of clinic health records and from the electronic card reading system used to process the trial payments. However, due to technical issues in implementation of the card reading system, it was not possible to extract outcome data from this source. As such, outcome data were also obtained from the personal clinic books of women participating in the trial. Specifically, participants were invited to attend the clinic shortly after the end of the trial with their clinic book for collection of the primary outcomes. For women who however did not attend the clinic for that purpose, data were extracted from the clinic register. Only very limited data on any treatment referrals beyond routine visits were collected and as such this primary outcome (5 of 5 in the Protocol) was dropped from the planned analysis. In addition, there were only data available for the primary outcomes of delivery at a healthcare facility and child immunisation and maternal PNC visits for those women with clinic book available for data extraction (only antenatal visits were extracted from clinic registers).

It was planned that data for the secondary outcomes would be derived from clinic records, the electronic card reading system and a series of four follow-up telephone interviews (after enrolment in all women; 2 weeks after delivery in all women who do not give birth in a facility; 6 months after EDD in 50% of women; 12 months after delivery in the same 50% of women). However, as well as issues with the card reader system, the interviews at 6 and 12 months after delivery were combined into one survey which was primarily delivered towards 12 months after delivery, after some 6-month interviews had already been conducted. The following secondary outcomes were dropped as data were not collected: mother’s perception of infant health at 6 and 12 months post-delivery, screening and control of infections for mothers and foetus/baby during pregnancy and postnatal periods.

Perinatal and maternal mortality were recorded if a woman (or family member) attended with their clinic book for data extraction, but completeness of the reporting has not been verified. The remaining outcomes were collected as part of the 6–12-month survey but the survey completion rate was ultimately relatively low, and as such, we have reported available data for the specified secondary outcomes at 6-12 months but have not carried out any formal statistical analyses. The completion rate of the baseline survey was high, but not sufficiently high to use these data to adjust our analyses for maternal characteristics such as socioeconomic factors.

Additional secondary outcomes of the counts of ANC, child immunisation and PNC clinic visits (both with and without applying any eligibility criteria regarding the timing of visits or maximum for each type) per woman were analysed and reported; this was not listed in the original Protocol, where the focus was on the primary outcomes of the *proportion* of each appointment type attended. Analysing the total counts in each case also allows for capture of the potential impact of earlier commencement of ANC care, and creates model outputs useful for health economic analysis. A further additional secondary outcome of GA at enrolment to the trial was also added, to evaluate whether the trial intervention encouraged earlier engagement with antenatal care (whereas the primary outcomes only relate to events following enrolment of each woman). This outcome was available for a large majority of women enrolled in the study.

Vanhuyse F, *et al. BMJ Open* 2022; 12:e055921. doi: 10.1136/bmjopen-2021-055921

The published Protocol stated that the analysis of primary outcomes would use logistic regression for binary outcomes and ordinal regression for ordinal outcomes, with a single pooled effect estimate for the intervention across these outcomes estimated using independence estimating equations. However, since delivery and postnatal outcome data were missing in a substantial proportion of participants, we planned to allow for dependence (i.e., correlation) between each of the outcome variables for each woman using structured random effects models. To facilitate this, visit counts for each woman were analysed as repeated binary observations rather than as ordinal variables.

Appendix S2 – Summary of available mortality data

These data have been obtained from both free-text notes in the study visit records and from information collected in the telephone surveys. It is therefore difficult to gauge the level of ascertainment of these adverse outcomes and the level of completeness will also depend on engagement with care and follow-up, which differed between the control and intervention groups.

Among the 2,949 women enrolled into the control arm, there was one record of intrauterine death of the foetus, one record of a stillbirth, 17 records of neonatal deaths (immediately or up to 1 week following delivery), 33 records of infant deaths up to 18 months after delivery and no records of maternal deaths.

Among the 2,522 women enrolled into the intervention arm, there were two records of intrauterine deaths of the foetus, three records of stillbirth, 23 records of neonatal deaths (immediately or up to 1 week following delivery), 45 records of infant deaths up to 18 months after delivery and two records of maternal deaths. The maternal deaths were both recorded at data extraction from maternal clinic books and appear to have occurred within a year of delivery, but not in the immediate neonatal period.

Vanhuyse F, *et al.* *BMJ Open* 2022; 12:e055921. doi: 10.1136/bmjopen-2021-055921

Additional Tables

Table S1: Cluster-level summaries of the enrolled women included in the primary analysis

Variable	Control clinic	Intervention clinic	ICC
N women per cluster in analysis	113 (67-174;21-301)	77 (31-158, 17-313)	—
**Baseline characteristics**			
Median age	26 (25-27, 22-29)	26 (25-27, 23-30)	—
Proportion nulliparous	0.22 (0.16-0.28, 0.004-0.37)	0.19 (0.10-0.26, 0-0.38)	—
Median GA at enrolment (days)	158 (151-166, 136-179)	153 (149-159, 139-183)	—
Proportion high-risk pregnancies	0.20 (0.11-0.24, 0.06-1)	0.17 (0.14-0.38, 0.06-0.94)	—
**Primary outcomes**			
Mean proportion attendance at eligible ANC clinic appointments (following scheduled visits)	0.61 (0.54-0.67, 0.48-0.75)	0.70 (0.65-0.75, 0.50-0.82)	0.028*
Proportion delivery at health facility	0.92 (0.89-0.96, 0.83-1)	0.90 (0.86-0.94,0.71-1)	0.012†
Proportion attendance eligible PNC clinic appointment<br>(at least one 4-12mo)	0.85 (0.76-0.90, 0.49-1)	0.85 (0.77-0.90, 0.47-0.94)	0.087†
Mean proportion attendance at child immunisation appointments (capped at 4)	0.85 (0.79-0.90, 0.71-1)	0.89 (0.86-0.91, 0.76-0.98)	0.011*

Data shown as median (interquartile range, range) by cluster.
ICC values calculated as $(var(b_i))/(var(b_i)+var(e_{ij}))$ from a linear mixed model with adjustment for intervention and subcounty, where $var(b_i)$ is cluster-level random intercept variance and $var(e_{ij})$ is the residual variance. *Linear mixed model fitted to overall proportion of visits attended as outcome for each woman. †Linear mixed model fitted to binary outcome data (i.e., 0 for no visit and 1 for attendance).

Vanhuyse F, *et al. BMJ Open* 2022; 12:e055921. doi: 10.1136/bmjopen-2021-055921

Table S2: Effect of conditional cash transfers on primary outcome measures for the sensitivity analysis only including the 12 intervention clinics and the 12 control clinics with the highest proportion of enrolled women with payment within 31 days of first ANC clinic visit.

	**Control clinic**	**Intervention clinic**		
**Primary outcome measures**	***n/N*** **(%)**	***n/N*** **(%)**	**OR (95% CI)**	**aOR (95% CI); *P***
Attendance at eligible ANC clinic appointments (following scheduled visits)*	3491/5335 (65)	3720/5775 (64)	0.78 (0.55-1.11)	0.80 (0.55-1.17); *P*=0.25
Delivery at health facility†	575/624 (92)	754/838 (90)	0.31 (0.11-0.88)	0.32 (0.11-0.92); *P*=0.04
Attendance at eligible PNC clinic appointment (at least one 4-12mo)‡	503/624 (81)	680/835 (81)	0.88 (0.45-1.74)	0.92 (0.47-1.82); *P*=0.81
Attendance at child immunisation appointments (capped at 4)†	2109/2496 (84)	2960/3352 (88)	1.65 (0.89-3.05)	1.72 (0.93-3.18); *P*=0.08
Pooled intervention estimate	—	—	0.89 (0.65-1.22)	0.92 (0.66-1.27); *P*=0.60

aOR, adjusted odds ratio; OR, odds ratio. *Available for all women included in primary analysis except three (with data from clinic book but no available EDD or ADD). †Available for 1462/3279 women with data obtained from clinic books. ‡Available for 1459 women with data obtained from clinic books and with available ADD or EDD. Available data for attendances and eligible appointments are summed over all women for the *n*/N values.

Vanhuyse F, *et al. BMJ Open* 2022; 12:e055921. doi: 10.1136/bmjopen-2021-055921
