# Supplementary material for: Effectiveness of conditional cash transfers (Afya credits incentive) to retain women in the continuum of care during pregnancy, birth and the postnatal period in Kenya: a cluster-randomised trial
Source: BMJ Open. 2022 Jan 6;12(1):e055921. doi: 10.1136/bmjopen-2021-055921 (PMC8739676; doi:10.1136/bmjopen-2021-055921)

BMJ Publishing Group Limited (BMJ) disclaims all liability and responsibility arising from any reliance placed on this supplemental material which has been supplied by the author(s)

Figure S1 Success or failure of prompt payment in individual women (● at 1 or 0), the overall success proportion (blue line) and a 31-day moving average (red line) divided by control and intervention clinics.

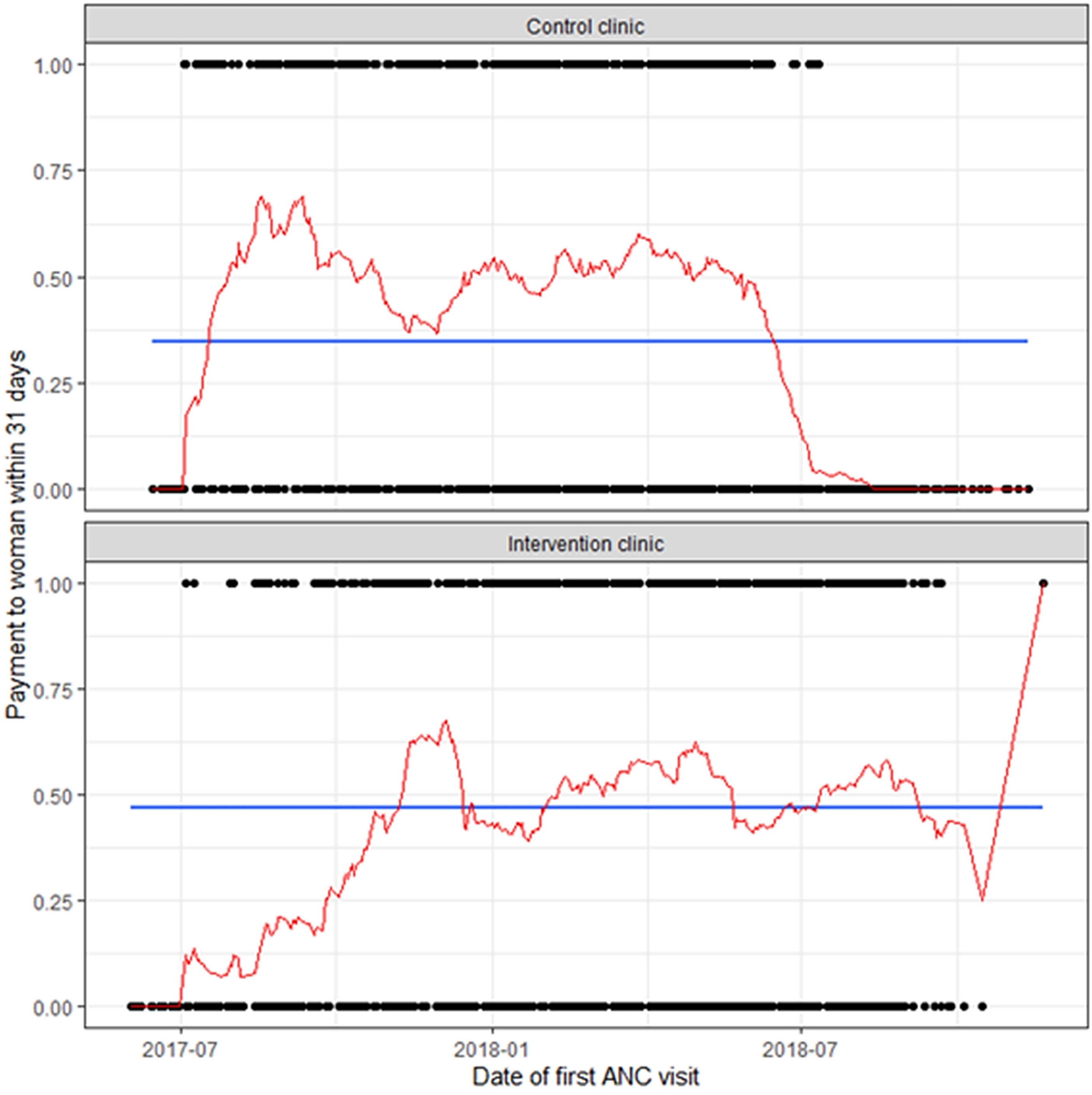

Supplement: Supplementary data [file bmjopen-2021-055921supp002.pdf]
