# Supplementary material for: Effectiveness of conditional cash transfers (Afya credits incentive) to retain women in the continuum of care during pregnancy, birth and the postnatal period in Kenya: a cluster-randomised trial
Source: BMJ Open. 2022 Jan 6;12(1):e055921. doi: 10.1136/bmjopen-2021-055921 (PMC8739676; doi:10.1136/bmjopen-2021-055921)

Figure S3 Success or failure of prompt payment in individual women (● at 1 or 0), the overall success proportion (blue line) and a 31-day moving average (red line) in individual intervention clinics.

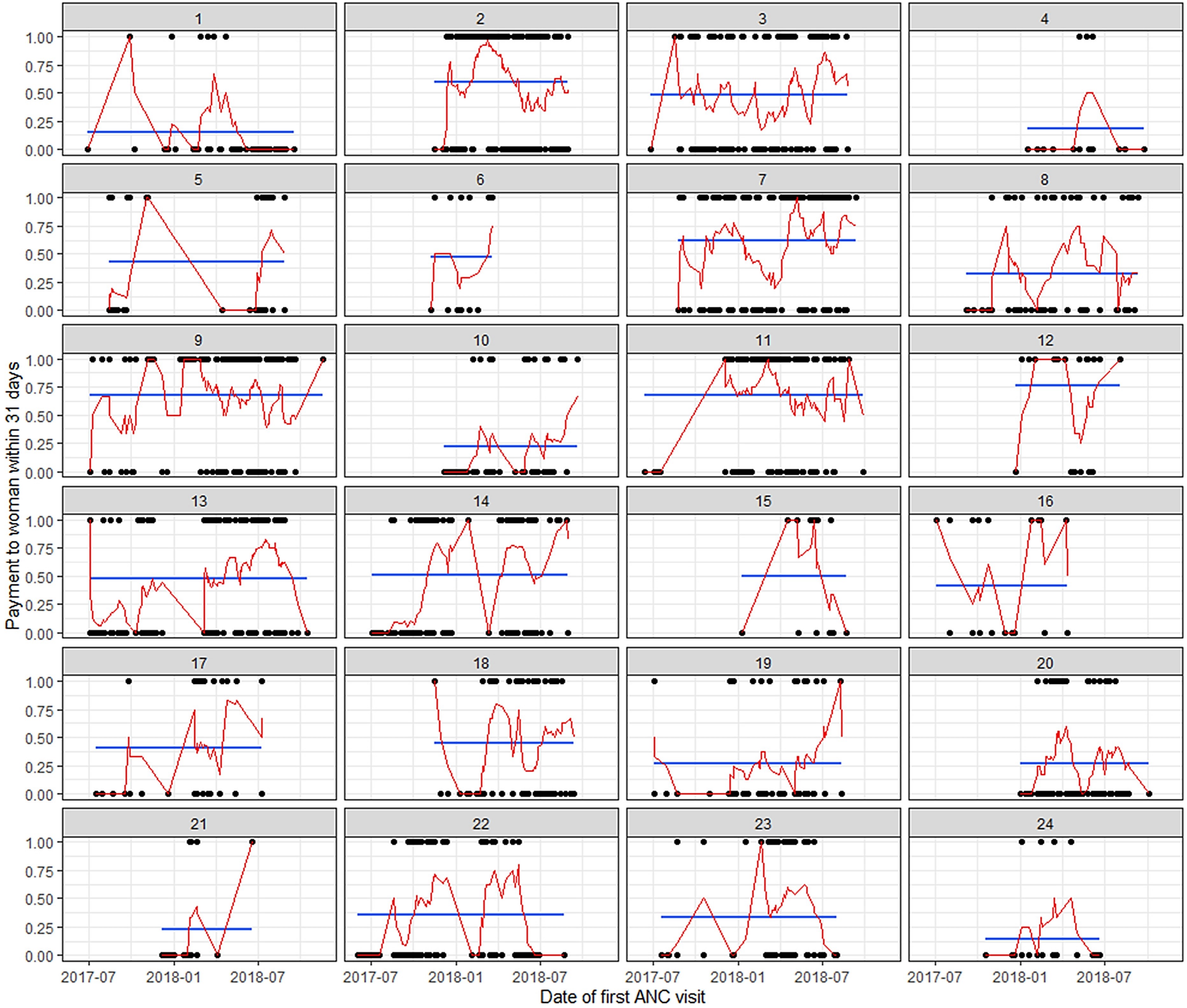

Supplement: Supplementary data [file bmjopen-2021-055921supp004.pdf]
