# Supplementary material for: Effectiveness of conditional cash transfers (Afya credits incentive) to retain women in the continuum of care during pregnancy, birth and the postnatal period in Kenya: a cluster-randomised trial
Source: BMJ Open. 2022 Jan 6;12(1):e055921. doi: 10.1136/bmjopen-2021-055921 (PMC8739676; doi:10.1136/bmjopen-2021-055921)

Figure S4 Exploratory analysis of the clinic-level relationship between proportion of women paid within 31 days of their first ANC clinic visit and the overall attendance proportion for eligible ANC visits. The graph is divided according to control and intervention clinics, with separate linear regression line (blue, with shaded 95%CI) and median percentage women with prompt payment (vertical black line) for each group.

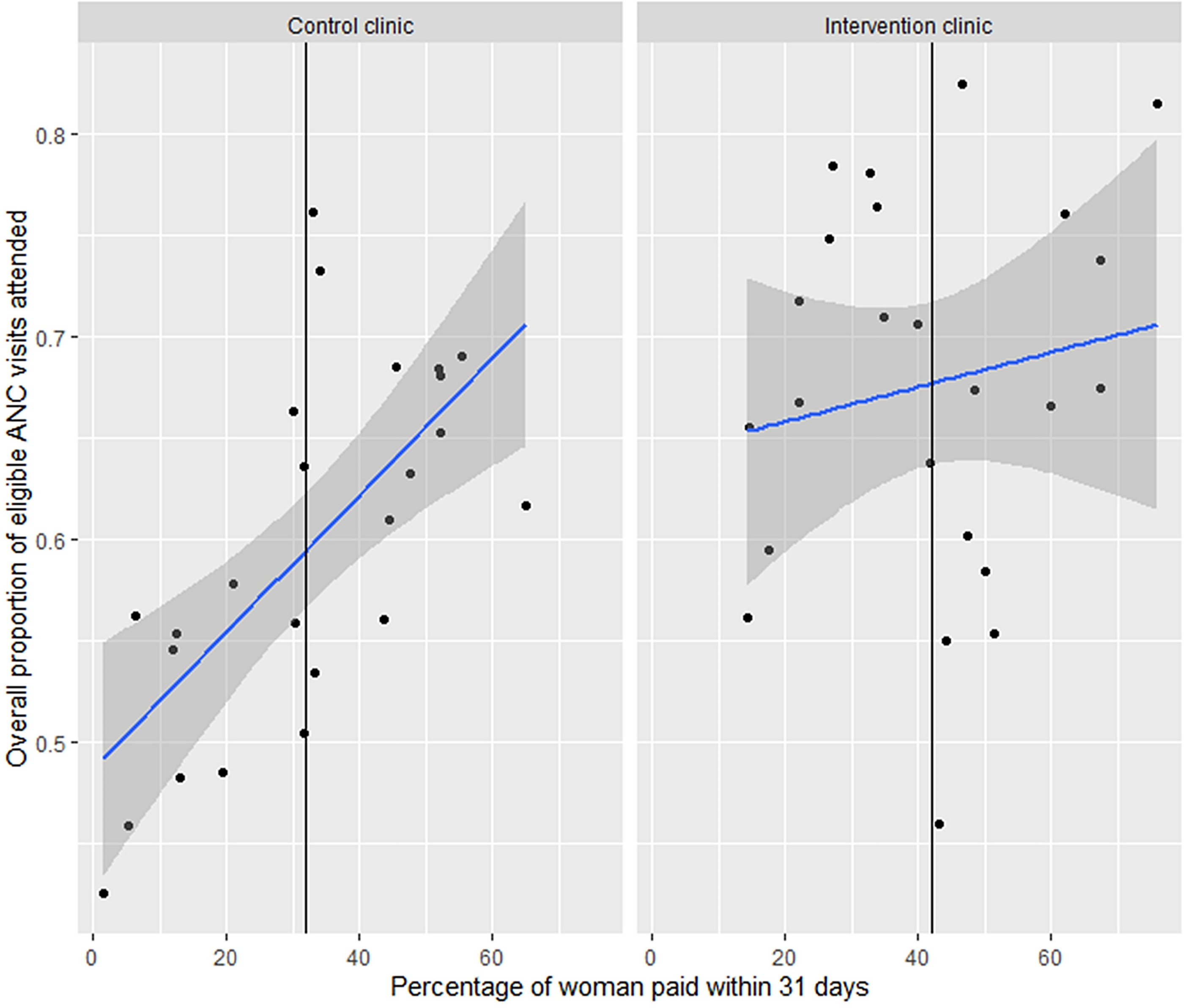

Supplement: Supplementary data [file bmjopen-2021-055921supp005.pdf]
