# Supplementary material for: Effectiveness of conditional cash transfers (Afya credits incentive) to retain women in the continuum of care during pregnancy, birth and the postnatal period in Kenya: a cluster-randomised trial
Source: BMJ Open. 2022 Jan 6;12(1):e055921. doi: 10.1136/bmjopen-2021-055921 (PMC8739676; doi:10.1136/bmjopen-2021-055921)
Supplement: Author's manuscript [file bmjopen-2021-055921.draft_revisions.pdf]

BMJ Open

BMJ Open is committed to open peer review. As part of this commitment we make the peer review history of every article we publish publicly available.

When an article is published we post the peer reviewers' comments and the authors' responses online. We also post the versions of the paper that were used during peer review. These are the versions that the peer review comments apply to.

The versions of the paper that follow are the versions that were submitted during the peer review process. They are not the versions of record or the final published versions. They should not be cited or distributed as the published version of this manuscript.

BMJ Open is an open access journal and the full, final, typeset and author-corrected version of record of the manuscript is available on our site with no access controls, subscription charges or pay-per-view fees (http://bmjopen.bmj.com).

If you have any questions on BMJ Open's open peer review process please email info.bmjopen@bmj.com

BMJ Open

Conditional cash transfers to retain women in the continuum of care in Kenya: Evaluating the Afya credits incentive for improved maternal and child health

Journal:	*BMJ Open*
Manuscript ID	bmjopen-2021-055921
Article Type:	Original research
Date Submitted by the Author:	29-Jul-2021
Complete List of Authors:	Vanhuyse, Fedra; Stockholm Environment Institute,<br>Stirrup , Oliver ; University College London, Institute for Global Health<br>Odhiambo, Aloyce; Safe Water and Aids Project (SWAP)<br>Palmer, Tom; University College London, Institute for Global Health<br>Dickin, Sarah; Stockholm Environment Institute<br>Skordis-Worrall, Jolene; UCL, Institute for Global Health<br>Batura, Neha; University College London, Institute for Global Health<br>Haghparast-Bidgoli, Hassan; University College London, Institute for Global Health<br>Mwaki, Alex; Safe Water and Aids Project (SWAP)<br>Copas, Andrew; University College London, Institute for Global Health
Keywords:	PUBLIC HEALTH, Community child health < PAEDIATRICS, Maternal medicine < OBSTETRICS

SCHOLARONE™
Manuscripts

I, the Submitting Author has the right to grant and does grant on behalf of all authors of the Work (as defined in the below author licence), an exclusive licence and/or a non-exclusive licence for contributions from authors who are: i) UK Crown employees; ii) where BMJ has agreed a CC-BY licence shall apply, and/or iii) in accordance with the terms applicable for US Federal Government officers or employees acting as part of their official duties; on a worldwide, perpetual, irrevocable, royalty-free basis to BMJ Publishing Group Ltd ("BMJ") its licensees and where the relevant Journal is co-owned by BMJ to the co-owners of the Journal, to publish the Work in this journal and any other BMJ products and to exploit all rights, as set out in our licence.

The Submitting Author accepts and understands that any supply made under these terms is made by BMJ to the Submitting Author unless you are acting as an employee on behalf of your employer or a postgraduate student of an affiliated institution which is paying any applicable article publishing charge ("APC") for Open Access articles. Where the Submitting Author wishes to make the Work available on an Open Access basis (and intends to pay the relevant APC), the terms of reuse of such Open Access shall be governed by a Creative Commons licence – details of these licences and which Creative Commons licence will apply to this Work are set out in our licence referred to above.

Other than as permitted in any relevant BMJ Author's Self Archiving Policies, I confirm this Work has not been accepted for publication elsewhere, is not being considered for publication elsewhere and does not duplicate material already published. I confirm all authors consent to publication of this Work and authorise the granting of this licence.

For peer review only

Title: Conditional cash transfers to retain women in the continuum of care in Kenya: Evaluating the Afya credits incentive for improved maternal and child health

Corresponding author: Fedra Vanhuyse, Stockholm Environment Institute, Linnégatan 87D, Box 24218, 104 51 Stockholm, Sweden.

Email: fedra.vanhuyse@sei.org

Authors: Fedra Vanhuyse*[1], Oliver Stirrup[2], Aloyce Odhiambo[3], Tom Palmer[2], Sarah Dickin[1], Jolene Skordis[2], Neha Batura[2], Hassan Haghparast-Bidgoli[2], Alex Mwaki[3] and Andrew Copas[2]

[1] Stockholm Environment Institute, Linnégatan 87D, Box 24218, 104 51 Stockholm, Sweden.

[2] Institute for Global Health, University College London, London, UK.

[3] Safe Water and AIDS Project (SWAP), P.O. Box 3323, Kisumu 40100, Kenya.

Key words: public health, community child health, maternal medicine

Word count: 4,840

For peer review only - http://bmjopen.bmj.com/site/about/guidelines.xhtml

Abstract

Objectives

Assess the impact of Conditional Cash Transfers (CCTs) to retain women in the continuum of care (antenatal care (ANC) visits, delivery at facility, postnatal care (PNC) visits and child immunization)

Setting

We conducted an unblinded cluster-randomized controlled trial in 48 health facilities in Siaya County, Kenya from May 2017 to December 2019. Health facilities were randomised 1:1 to arms.

Participants

2922 women were recruited to the control arm and 2522 to the intervention arm. Women were recruited at first ANC visit.

Interventions

For each attended appointment, women would receive 450KSH in the intervention arm and 50KSH in the control arm. An electronic system was to capture the visits and trigger automatic payments to the participant.

Primary and secondary outcome measures

Primary outcomes were ANC attendances, delivery at facility, any PNC 4-12 months after delivery, and childhood immunization. Secondary outcomes include total number of visits attended and gestational age at first ANC visit.

Results

Despite challenges with the electronic system and data completeness, we found a significantly higher proportion of appointments attended for ANC (67% vs. 60%, adjusted OR (aOR) 1.90; 95% CI (1.36-2.66)) and child immunization (88 vs 85%; aOR 1.74; 95% CI 1.10-2.77) in intervention arm than control arm. The pooled odds ratio across all four attendance types was 1.64 (1.28-2.10). No intervention effect was seen considering delivery at the facility and any PNC attendance separately.

Conclusions

Demand-side financing incentives, such as CCTs, can improve attendance for health care appointments. However, attention needs to be paid to the technology used to incentivise women, the barriers that remain for delivery at facility and PNC visits, and encouraging women to attend ANC visits within the recommended WHO timeframe.

Trial registration

NCT03021070; clinicalTrials.gov

Strengths and limitations of this study

- Technical issues with the electronic system and at times low participation of health workers resulted in not all visits being registered, and only 26% of the payments triggered automatically.
- Manual payments needed to be triggered, which resulted in delays.
- This delay in payment could have diluted the impact of the intervention and prevented service utilization in the latter stages of pregnancy or after babies were born.
- Manual data abstraction from clinic registers and from the women's maternal clinic books was necessary and resulted in limited data on PNC and child immunization.
- The potential bias is however limited to a degree by our approach to analysis in which all outcomes are modelled simultaneously, in which we effectively use the ANC attendance data to predict (i.e., 'impute') the other outcomes.

For peer review only - http://bmjopen.bmj.com/site/about/guidelines.xhtml

Introduction

Every year an estimated 295,000 maternal deaths occur globally, with 99% occurring in low and middle-income countries, and almost two thirds in Africa[1]. In Sub-Sahara Africa, the maternal mortality rate was 542 deaths per 100,000 live births in 2017[1]. In Kenya, the maternal mortality rate in 2014 was 362 deaths per 100,000 live births[2], categorized as “high” according to the World Health Organization (WHO). Globally, an estimated 2.4 million infants die in the first month of life (40% of all deaths) and 1.6 million at age 1–11 months (25%)[3]. Sub-Saharan Africa has the highest neonatal mortality rate at a median of 27 deaths per 1,000 live births and the highest under-five mortality rate, with a median of 76 deaths per 1,000 live births[3]. In Kenya, the median infant mortality rate was 21 deaths per 1,000 live births and the median under-five mortality rate was 43 deaths per 1,000 live births[2], which are amongst the highest in the region.

Most maternal and neonatal deaths are avertible through the use of healthcare interventions that prevent or manage pregnancy-related complications such as postpartum haemorrhage and infectious diseases[4,5]. The importance of health service utilisation for maternal and child health outcomes has been extensively documented, including for antenatal care (ANC)[6–8], facility delivery[9, 10], postnatal care (PNC)[11] ,and across the continuum of care[12]. In Kenya, while 96% of women receive some form of ANC, less than three in five receive the four ANC visits that the WHO recommends[2]. Only one in five have their first ANC visit during the first trimester as recommended by the WHO[2], which would allow to monitor the health of both mother and child more effectively. Almost four in ten babies are delivered at home, and 62% of new-borns do not receive a PNC check-up in the first week after birth[2]. Almost half (47%) of mothers do not receive a PNC check-up in the first two days after birth[2]. Lower attendance at healthcare facilities could be due to a lack of skilled health workers, poverty, distance, lack of information, inadequate services and cultural practices[13]. Other research[14] reported costs charged for ANC visits, nurses’ behaviour, and the timing of the visits as the main barriers for attending ANC visits. In Kenya, a survey showed that financial barriers (costs of care for other children, food, new clothes), and lack of transport and distance to health care facilities were the main barriers[15].

Aside from programmes to improve the quality and reach of the service (supply side interventions), demand side financing interventions have been set up to incentivise women to attend visits. Examples of such interventions include mobile phone text message reminders for ANC visits in Zanzibar[16] and for PNC visits in Tanzania[17], as well as the use of conditional cash transfers (CCTs). In Kenya, a conditional cash transfer intervention in Vihiga County was found to increase facility delivery by 7.9 percentage points[18]. A recent study in Nigeria[19] found that payments for retention from ANC to PNC resulted in more women attending the visits (26% of women in the intervention arm compared to 12% in the control arm), leading to a 22% reduction in the stillbirth rate. Recent systematic reviews on the demand-side interventions for maternal care[20] and on CCTs[21–24] found increased utilisation of services, but not always better outcomes.

The Afya trial aims to test the effectiveness of a conditional cash transfer to retain women in the continuum of care, from their first ANC visit until their children reach 1 year of age in Siaya County, Kenya. The CCT aimed at tackling multiple barriers to care, as described in the trial’s protocol[25]: women would receive equal-sized cash transfers following a visit (ANC, delivery at facility, PNC visit and childhood immunization), as well as a reminder for their visit by text message and medical staff would be trained in the technology and incentivised for each woman they enrolled in the trial. Unlike a study in Nigeria[19], in Afya, the CCT was done through a card reader system rather than cash and, additionally, it initiated transfers for each individual visit, rather than being conditional on receiving an entire package of care. In this paper, we present the results of the impact of the Afya trial.

For peer review only - http://bmjopen.bmj.com/site/about/guidelines.xhtml

Methods

Study setting, design and randomisation

We conducted a cluster randomized controlled trial, with equal allocation to intervention and control arms, in Siaya county, Kenya. The units of randomisation were 48 Level 2 or 3 health facilities (Dispensaries and Health Centres, respectively). The randomisation of centres was stratified by sub-County and ensured equal allocation to study arms within each stratum without any overlap of catchment areas. The selection of trial facilities and randomisation were conducted simultaneously at a public meeting. The randomisation of centres was stratified by sub-County and ensured equal allocation to study arms within each stratum without any overlap of catchment areas, as described in detail in the trial protocol[25]. In summary, the implementing partner wrote the names of 60 shortlisted facilities on pieces of paper and folded them to hide the names, then included them in transparent boxes, one for each sub-county. Each subcounty had an (even) number of facilities to recruit to the trial proportional to subcounty size. The health management teams from each subcounty selected the pieces of paper, one by one. The first was allocated to intervention, second to control. However, before each facility was formally recruited, a check was made that the catchment area for the facility did not overlap with those on any facilities already recruited, and if it did then a replacement was selected.

Health facility staff determined whether a pregnant woman met the study eligibility criteria by administering screening questions at the end of her first ANC visit. All women meeting the criteria were eligible for recruitment during the study recruitment period. Criteria for enrolment were: women attending their first ANC visit; long-term resident of the catchment area served by the health facility (living in the area for at least 6 months); access to a mobile phone that belongs either to themselves or to a member of their household or person whom they trust. Individual consent was required for trial participation, and refusals were recorded.

Intervention

The intervention was a CCT payment for each facility appointment attended for ANC, delivery, postnatal care, and childhood immunization. Detailed definitions can be found in Appendix S1. For each scheduled health visit made following enrolment, women in the intervention arm received a cash transfer of KSH 450 (4.5 USD) on their mobile phones. Women at the control clinics were granted KSH 50 (0.5 USD) mobile phone airtime for each scheduled visit to encourage them to bring their clinic booklet to appointments. In both trial arms, women were issued with a trial card at recruitment. Payments to the women were triggered by tapping the card on a card reader, which also logged the visit in an online portal[26]. In the event of problems with the card reader, or if the woman did not bring her card to the appointment, payments could alternatively be processed manually. Manual payments were processed by the implementing partner if contacted by the woman (or facility) after verifying the appointment with the facility. Nurses were given KSH 400 (4 USD) per woman enrolled during the trial, and an additional KSH 100 (1USD) per woman enrolled at the end of the trial for their collaboration in the trial. These payments were transferred to the nurses electronically. Details of the intervention design are presented in the protocol[25].

Patient and Public involvement statement

Patients or the public were not involved in the design, conduct, reporting, or dissemination plans of the research. The assessment of the burden of the intervention on patients is reported in[27].

Ethics statement

Ethical approval was granted by Maseno University in Kenya (MSU/DRP/MUERC/00294/16).

For peer review only - http://bmjopen.bmj.com/site/about/guidelines.xhtml

Trial outcomes

The primary outcomes of the trial were: 1) attendance or missed attendance at each eligible ANC appointment after recruitment; 2) delivery at a health facility; 3) attendance for at least one PNC appointment 4-12 months after delivery; 4) attendance or missed attendance at each expected child immunization appointment. We define 'eligible visits' for the purpose of statistical analysis of the impact of the intervention within a clearly defined framework, but all scheduled pre- and post-natal clinic visits should have triggered a payment. The following secondary outcomes are also reported and analysed according to trial arm: 1) attendance of all eligible maternal, new-born and child health (MNCH) care visits, both prenatal and postnatal, for each woman; 2) the count of attended ANC and child immunisation clinic visits eligible for the primary outcome variables for each woman; 3) the total number of ANC, child immunisation and PNC clinic visits (without applying any eligibility criteria) for each woman; 4) gestational age (GA) at first ANC visit (and enrolment to study). The secondary outcomes of clinic visit counts and GA at first ANC visit were not listed in the trial protocol but were prespecified in the statistical analysis plan to aid interpretation of the study results. Some planned secondary outcomes are reported without formal statistical analysis because of low levels of data completeness, and there is no reporting of treatment referral because of very limited availability of data. Appendix S1 contains details of changes made to the data collection and analysis compared to the protocol, arising from data collection challenges.

Data collection

Data were collected throughout the trial from an electronic card reading system, and baseline and follow-up surveys. The electronic card reading system captured the enrolled women's phone number, expected delivery date (EDD), parity, the clinic she enrolled at, the visits she attended and the payments she received. The baseline survey, carried out by telephone following enrolment, collected socio-demographic data. We initially planned to conduct follow-up interviews with 50% of enrolled women at 6 and 12 months post-delivery to collect secondary outcomes. However, due to limited resources and lower than anticipated response rates after some 6-month interviews had been conducted, we adopted a pragmatic approach and conducted one follow-up survey at around 12 months after delivery.

Problems with the implementation of the technical system and periods of disengagement from clinic staff resulted in a large proportion of visits not being registered on the system. Therefore, data on women's visits were manually extracted from clinic health records after the trial was completed. Trial staff visited each facility, after arranging for trial participants to be invited to attend the facility with their maternal clinic book. Data on the primary outcomes of ANC, child immunisation and PNC visits and delivery at a healthcare facility were extracted from maternal clinic books. For women who did not attend the data extraction, data on the primary outcome of ANC visits were extracted from health facility registers, but data on other visits were not available from this source. Any missing payments were transferred to the participating women following the manual data extraction at the end of the trial.

Data on payments made to trial participants, whether triggered by the card reader or manually, were extracted from the trial portal. These data are reported in the process evaluation paper, along with details of the challenges with the electronic system[27].

Sample size

We analysed all primary outcomes jointly, to maximise power, aid interpretation and minimise testing (see Statistical Analysis). However, for our sample size calculation, we considered the power to detect

For peer review only - http://bmjopen.bmj.com/site/about/guidelines.xhtml

an effect of the intervention on one primary outcome, which we also assumed to be a binary indicator that all attendances were made, as this is simple and conservative in the power achieved. The expected prevalence of these indicators in the control arm ranged between 30 and 80%. In the absence of specific information on the likely intra cluster correlation (ICC) we considered a range between 0.005 (low) and 0.025 (moderate). Our planned sample size was 48 clusters (24 per arm) and an average cluster size of 150. At a low ICC, the design effect (DE) would be 1.745 and hence the effective sample size (ESS) would be 2,063 participants per study arm. At a moderate ICC, the DE would be 4.725 and hence the ESS 762 per arm. Power to detect absolute differences is lowest when the prevalence is 50% and highest when the prevalence is either high (towards 100%) or low (towards 0%). Here we considered the prevalence in the control arm to range between 50% ('worst-case scenario') and 80% ('best-case scenario'). We considered the standard 5% significance level. If the prevalence of the outcome is 50% in the control arm, the sample size provides 80% power to detect an improvement to 54.5% in the intervention arm if the ICC is low and 57.5% if the ICC is moderate. If the prevalence of the outcome is 80% in the control arm, the sample size provides 80% power to detect an improvement to 83.5% if the ICC is low and 85.5% if the ICC is moderate.

Data definitions and processing

For the purpose of statistical analysis, eligible ANC visits were defined based on the recorded 'next scheduled visit date' and noting that this was typically 4 weeks later each time. For each recorded visit starting with enrolment, we evaluated whether the next observed ANC visit was within ±2 weeks of the 'next scheduled visit date': recording a successful ANC visit if yes, but a missed visit if not. However, we did not count the visit as either successful or missed if the next observed visit was more than 2 weeks early compared to the next scheduled visit or delivery occurred within 2 weeks of a 'missed' scheduled visit. Whether that next visit is early, on time, or late, we assessed subsequent visit attendances in the same way. We created hypothetical scheduled visits every 4 weeks for any gaps in observations, judged according to the same criteria. The 'successful' and 'missed' appointments were then summed over all scheduled and hypothetical appointments for each woman.

For the primary outcomes of PNC visits, we defined a binary indicator of one or more PNC visit 4-12 months postpartum. This was used to provide a simple indicator of engagement with postnatal care for each woman, given that the appropriate number of PNC visits may differ between women. We defined child immunisation visits as the total number of visits recorded post-partum (excluding vaccination at delivery, but without other time restrictions) truncated at a maximum of four, since that is the typical number required for full immunisation.

Retention in the full continuum of antenatal, perinatal, and postnatal care (a secondary outcome) was defined as a binary indicator of attendance of all eligible ANC, child immunisation and PNC visits and delivery at a healthcare facility for each woman and is available for those women who brought their clinic book for data extraction.

Statistical analysis

The primary outcomes of attendance of eligible ANC and child immunisation appointments comprise repeat binary observations for each individual woman, whilst the primary outcomes of delivery at a health facility and PNC attendance between 4–12 months are each single binary variables for each woman. A summary odds ratio is presented as the main effect measure for the trial, estimated from a model that assumes the odds ratio is the same across the four primary outcomes. We also report separate effect estimates for each primary outcome. A mixed effects logistic regression model was used to jointly analyse the primary outcomes, which implicitly imputes the missing information on facility delivery, PNC and child immunisation visits based on ANC attendance for women who did not bring their maternal clinic book for data extraction. At the level of each woman correlated random

For peer review only - http://bmjopen.bmj.com/site/about/guidelines.xhtml

effects were specified for (1) attendance of ANC clinic visits and (2) grouped outcomes of delivery at a healthcare facility, attendance of vaccination visits and attendance of at least one PNC clinic visit at 4–12 months post-partum. For the clinic-level random effects, an unstructured covariance matrix was used with random effect terms for (1) ANC visits, (2) delivery at a healthcare facility and (3) PNC and child immunisation visits.

The secondary outcomes of counts of ANC clinic, PNC clinic and child immunisation visits were analysed using a multivariate Poisson mixed effects model, with random intercept terms at patient and the clinic levels. Marginal mean differences in counts between intervention groups were estimated. The secondary outcome of GA at enrolment to the trial was analysed using a linear mixed effects model, with random intercept term at clinic level. Retention in the full continuum of care was analysed using a logistic regression mixed effects model, with random intercept term at clinic level. As the completion rate of the follow-up survey was lower than expected, the secondary outcome data obtained is reported in a descriptive summary but not compared between trial arms.

Our main analyses are conducted as randomised (i.e., intention to treat) but a 'per-protocol' style sensitivity analysis of the primary outcomes was also prespecified. As there was not a clear division between clinics that did and did not achieve the intended payment schedule, the sensitivity analysis included the 12 intervention clinics and the 12 control clinics with the highest proportion of enrolled women with payment within 31 days of first ANC clinic visit.

All analyses were adjusted by the baseline maternal parity ('0' vs '≥1'), by the presence of any maternal medical conditions leading to classification of the pregnancy as high-risk (HIV with or without ART, diabetes, hypertension, malaria, each coded with separate indicator variables) and by the clinic-level randomisation stratification variable of sub-county. Adjusted effect measures are considered the primary effect measures though unadjusted effect estimates are also reported. The analysis followed a pre-specified analysis plan.

As recommended by the MRC framework for the evaluation of complex interventions[28], a detailed process and economic evaluation will be published in two forthcoming papers[27, 29].

Results

The trial was conducted in 24 intervention and 24 control clinics and enrolled a total of 2522 women at intervention clinics and 2949 at control clinics over a period from May 2017 to December 2019 (Figure 1). Only 11 eligible women declined enrolment at intervention clinics and 58 at control clinics. Enrolment stopped before the target sample size of 7,200 could be reached due to delays arising from the nurses' strike during which enrolment was paused at many clinics (see, e.g.,[30] who discussed the strike and its impacts on health care delivery), and as the trial was intended to run until 2018 initially. The vast majority (5,388 or 98.5%) of women had data on ANC attendances, but data from maternal clinic books on all primary outcomes were available in a minority of women (2,262/5,388, 42.0%). Socio-demographic characteristics for all enrolled women are presented in Table 1, with cluster-level summaries in Table S1. Baseline survey data were available in 4,313/5,471 (78.8%) women, and a summary of selected fields by arm is presented in Table 2. Table 1 and 2 demonstrate very good balance between arms.

Table 1: Baseline and pregnancy characteristics of the enrolled women included in the primary analysis, obtained from enrolment data, clinic book and clinic registry data

Variable	Control clinic, *n* (%) or median (IQR)	Intervention clinic, *n* (%) or median (IQR)

For peer review only - http://bmjopen.bmj.com/site/about/guidelines.xhtml

Baseline characteristics			
Age	Years (median (IQR) [*n*])	26 (22-31) [2738]	26 (22-31) [2349]
Parity	0	606 (21)	488 (20)
	1	698 (24)	591 (24)
	2	587 (20)	485 (19)
	≥3	1007 (35)	926 (37)
GA at enrolment	Weeks+days (median (IQR) [*n*])	22+4 (17+4 to 27+2) [2898]	22+3 (17+2 to 27+0) [2487]
HIV status	Negative	2481 (86)	2142 (86)
	Positive, on treatment	398 (14)	322 (13)
	Positive, not on treatment	19 (1)	26 (1)
Diabetes	No	2889 (99.7)	2482(99.7)
	Yes	9 (0.3)	8 (0.3)
Hypertension	No	2892 (99.8)	2485 (99.8)
	Yes	6 (0.2)	5 (0.2)
Malaria	No	2603 (90)	2164 (87)
	Yes	295 (10)	326 (13)
Total high-risk pregnancies	No	2245 (77)	1850 (74)
	Yes	653 (23)	640 (26)
**Pregnancy Characteristics**			
GA at delivery	Weeks+days (median (IQR) [*n*])	39+3 (37+0 to 41+1) [1004]	39+0 (36+4 to 41+0) [1206]

IQR, interquartile range. GA gestational age

Table 2: Sociodemographic characteristics of the enrolled women from baseline survey

**Variable**		**Control clinic, *n* (%)**	**Intervention clinic, *n* (%)**
Enrolled women	Total *n*	2949	2522
Baseline survey	Available	2233 (76)	2080 (82)
	Missing	716 (24)	442 (18)
Self-rated maternal health	Very good	4 (0.2)	3 (0.1)
	Good	771 (35)	718 (35)
	Moderate	1445 (65)	1351 (65)
	Bad	12 (0.5)	6 (0.3)
	Very Bad	0 (0)	0 (0)
	Missing*	1 (0.04)	2 (0.1)
Maternal education level	None or only literacy	15 (0.7)	17 (0.8)
	Primary incomplete	631 (28)	540 (26)
	Primary complete	860 (39)	796 (38)
	Secondary incomplete	352 (16)	343 (16)
	Secondary complete	292 (13)	292 (14)
	University/college	79 (4)	91 (4)
	Don't know/other/missing*	4 (0.2)	1 (0.05)
Mode of travel to facility for enrolment visit	Public transport, e.g. bus	727 (33)	718 (35)
	Mini bus taxi	0 (0)	0 (0)
	Metered /taxi	4 (0.2)	7 (0.3)
	Walking	1485 (67)	1341 (64)

For peer review only

	Car	1 (0.04)	1 (0.05)
	Other	16 (0.7)	11 (0.5)
	Missing*	0 (0)	2 (0.1)
Travel time to facility for enrolment visit	<1 hour	1444 (65)	1314 (63)
	1-2 hours	741 (33)	699 (34)
	2-3 hours	43 (2)	60 (3)
	>3 hours	4 (0.2)	5 (0.2)
	Missing*	1 (0.04)	2 (0.1)

*Of those women with baseline survey recorded.

Primary and secondary outcomes

The proportion of eligible ANC appointments attended was significantly higher in the intervention arm compared to control (67 vs. 60%; aOR 1.90; 95% CI 1.36-2.66). A smaller increase was also demonstrated in the proportion of eligible immunisation appointments attended (88 vs 85%; aOR 1.74; 95% CI 1.10-2.77). For the other primary outcomes reporting was similar between arms (Table 3). The pooled aOR for the intervention effect giving a summary measure across all primary outcomes was 1.64 (95%CI 1.28-2.10), p<0.001. The intervention effect on the number of eligible ANC attendances, expressed as an adjusted marginal change (Table 3), was an increase of 0.31 (0.15 to 0.47). The adjusted marginal change in eligible immunisation attendances was not significant (0.14, -0.12 to 0.41). Increases in attendances were seen for all visit types when the eligibility requirements defined for the primary analysis were removed, thereby considering all healthcare visits (including any unscheduled visits). The intervention had no effect on the timing of first ANC visit, the mean GA at enrolment was 22.2 weeks for intervention and 22.3 weeks for control, or quite a few weeks after the recommended first visit by the WHO[31] but consistent with other studies in low- and middle income countries[32]. Postnatal surveys at 5-18 months after delivery were completed by a minority of women, selected outcomes are reported by arm in Table 4. Maternal and perinatal mortality were not systematically recorded, but the available data on these outcomes are summarised in Appendix S2. The ICC was 0.028 for the primary outcome of ANC visits, 0.012 for delivery at a health facility, 0.087 for attendance of at least one eligible PNC visit and 0.011 for immunisation visits.

Table 3: Effect of conditional cash transfers on primary and secondary outcome measures

	**Control clinic**	**Intervention clinic**		
**Primary outcome measures**	***n/N* (%)**	***n/N* (%)**	**OR (95% CI)**	**aOR (95% CI); *P***
Attendance at eligible ANC clinic appointments (following scheduled visits)*	5,827/9,736 (60)	5,741/8,595 (67)	1.95 (1.39-2.72)	1.90 (1.36-2.66); *P*<0.001
Delivery at health facility†	945/1,027 (92)	1,115/1,238 (90)	0.59 (0.26-1.35)	0.58 (0.25-1.33); *P*=0.20
Attendance at one or more eligible PNC clinic appointment (4-12 months after delivery)‡	831/1,027 (81)	1,016/1,235 (82)	1.26 (0.75-2.12)	1.25 (0.74-2.10); *P*=0.40
Attendance at child immunisation appointments (capped at 4)†	3,498/4,108 (85)	4,353/4,952 (88)	1.76 (1.10-2.80)	1.74 (1.10-2.77); *P*=0.02
Pooled intervention estimate	—	—	1.69 (1.32-2.17)	1.64 (1.28-2.10); *P*<0.001

For peer review only - http://bmjopen.bmj.com/site/about/guidelines.xhtml

Secondary outcome measures	Control clinic	Intervention clinic	
	***n/N* (%)**	***n/N* (%)**	**aOR (95% CI)**
Attendance at all eligible ANC and PNC visits, child immunisation appointments and delivery at a healthcare facility (per woman)	480/1,027 (47)	632/1,235 (51)	1.14 (0.82 to 1.57)
	Mean (95% CI) ¶, Median (IQR)	***Mean (95% CI) ¶, Median (IQR)***	**Average difference in mean (95% CI) ¶**
**Visit counts (eligible for primary outcome)**			
Total attendances at eligible ANC clinic appointments (following scheduled visits)*	2.04 (1.93-2.14) 2 (0-3)	2.34 (2.22-2.47), 2 (1-3)	0.31 (0.15 to 0.47)
Total attendances at eligible child immunisation appointments (capped at 4)†	3.33 (3.14-3.52), 4 (3-4)	3.48 (3.29-3.67), 4 (4-4)	0.14 (-0.12 to 0.41)
**Visit counts (no eligibility criteria applied)**			
Total attendances at ANC clinic appointments‡	2.05 (1.93-2.18), 2 (0-3)	2.42 (2.27-2.57), 2 (1-4)	0.37 (0.18 to 0.56)
Total attendances at PNC clinic appointments†	4.17 (3.90-4.44), 4 (2-7)	4.76 (4.46-5.06), 5 (2-7)	0.58 (0.19 to 0.98)
Total attendances at child immunisation appointments†	3.64 (3.40-3.88), 4 (3-5)	4.00 (3.75-4.26), 4 (4-5)	0.36 (0.02 to 0.71)
	**Mean (95% CI)**	**Mean (95% CI)**	**aΔ (weeks) (95% CI)**
GA at enrolment (weeks)*	22.3 (21.9-22.7)	22.2 (21.8-22.6)	-0.1 (-0.6 to 0.5)

aΔ, adjusted difference in mean; aOR, adjusted odds ratio; OR, odds ratio.
*Available for all women included in primary analysis except three (with data from clinic book but no available EDD or ADD).
†Available for 2265/5388 women with data obtained from clinic books. ‡Available for all women included in primary analysis.
¶Marginal value derived from multivariate Poisson mixed effects model applied to visit counts, estimated over the baseline characteristics of all 5388 women with data for at least one of the primary outcomes.
‡Available for 2262 women with data obtained from clinic books and with available ADD or EDD. Available data for attendances and eligible appointments are summed over all women for the *n*/N values.

Table 4: Data from follow-up surveys completed 5-18 months after delivery

**Variable**		**Control clinic**	**Intervention clinic**
Enrolled women	Total *n*	2,949	2,522
Survey answers 5-18 months after delivery			
Survey	Available	626 (21)	993 (39)
	Missing	2,323 (79)	1,529 (61)
Time from delivery at survey (days)		379 (273-463)	375 (273-469)
Self-rated maternal health	Very good	70 (11)	185 (19)
	Good	144 (23)	190 (19)
	Moderate	37 (6)	42 (4)
	Bad	4 (0.6)	3 (0.3)
	Very Bad	0 (0)	0 (0)

For peer review only - http://bmjopen.bmj.com/site/about/guidelines.xhtml

	Not asked in survey included	359 (57)	548 (55)
	Missing*	12 (2)	25 (3)
Exclusive breastfeeding to 6 months	Yes	244 (39)	396 (40)
	No	11 (2)	24 (2)
	Not asked in survey included	359 (57)	548 (55)
	Missing*	12 (2)	25 (3)
Family planning advice at last clinic visit	Yes	539 (86)	864 (87)
	No	58 (9)	78 (8)
	Missing*	29 (5)	51 (5)
Current method to prevent pregnancy	None	94 (15)	137 (14)
	NA: pregnant	3 (0.5)	4 (0.4)
	Yes: contraceptive	433 (69)	723 (73)
	Yes: natural methods	57 (9)	49 (5)
	Yes: other	9 (1)	29 (3)
	Missing*	30 (5)	51 (5)

Data shown as *n*, *n* (%) or median (IQR). Data were available for some women for both the planned '6 month survey' and the planned '12 month survey' (although the actual timing was not necessarily as planned), and in these the latter was used if completed 5-18 months after delivery as it contained questions on self-rated maternal health and age at end of exclusive breastfeeding. *Of those women with relevant survey data available.

Sensitivity analysis

A sensitivity 'per protocol' analysis of the primary outcomes was prespecified to only include those clinics and periods for which payments were being processed. However, as the correct functioning of the payment systems did not follow clear temporal divisions, the sensitivity analysis was conducted including those intervention and control clinics with a proportion of women with prompt payment (within 31 days) of their first ANC visit above the median within each arm.

We evaluated prompt payment from first visit among the 4,156 women with at least one ANC visit included in the primary analysis. There was a prompt payment in 743/2,141 (34.7%) women in the control clinics and in 943/2,015 (46.8%) in the intervention clinics (Figure S1). Across control clinics, the median proportion with prompt payment for first visit was 32.3%, with interquartile range (IQR) 17.8% to 46.1% (Figure S2). Across intervention clinics, the median proportion with prompt payment was 42.4%, with IQR 27.0% to 50.4% (Figure S3).

In the sensitivity analysis, there was no observed positive effect of the implementation on ANC attendance, and the pooled estimate of the intervention effect was negative but not statistically significant (Table S2). As this finding was surprising given the main results, we investigated the cluster-level association between prompt payment and ANC attendance. This revealed a stronger correlation in the control clinics than in the intervention clinics (Figure S4). Consequently, in our sensitivity analysis in terms of ANC attendance rates, we were unexpectedly comparing high performing control clinics with typical intervention clinics, which explains the change to the intervention effect from the main analysis.

Discussion

In this paper, we evaluated the impact of a demand side financing intervention, using CCTs, on the retaining pregnant women in the continuum of care from their first ANC visit until their children reach 1 year of age in rural Kenya. Previous evaluations of CCT programmes in the Sub Saharan Africa region have focussed on either increasing ANC visits[33], institutional deliveries[18, 33] or PNC visits[34]. Two other studies in the region evaluated the impact of demand side financing on the retaining women in the continuum of care. For the continuum of care, one study evaluated the impact of a subsidized

For peer review only - http://bmjopen.bmj.com/site/about/guidelines.xhtml

reproductive health voucher programme and the introduction of free maternity services in government facilities on ANC visits, facility birth and PNC visits in Kenya[35]. Another study[36] examined the impact of a national CCT pilot programme on the continuum of care in rural Nigeria. To our knowledge, this is the first evaluation of the impact of CCTs on retaining pregnant women in the continuum of care in Kenya and provides crucial evidence to inform policy and practice related to demand side financing mechanisms for improving maternal and new-born health outcomes.

Our main finding suggests that the intervention led to a modest increase in ANC clinic attendance and child immunisation appointments. This is consistent with evidence on the impact of demand side financing programmes on ANC service utilization from the sub–Saharan African region but less so with the evidence on child immunization. For example, a study set in Kenya[35] found that a subsidized reproductive health voucher programme and free maternity services improved early initiation of ANC as well as continuous use of care amongst ANC attendees in government facilities. Others[33, 36] also found that CCT programmes increased ANC attendance in rural Kenya and Nigeria, respectively. This implies that demand side financing interventions, whether using CCTs or vouchers can increase ANC service utilization, though the size of the impact might vary. It is possible that the increase in ANC service utilization might be greater if financial incentives such as CCTs and vouchers are combined with other policy measures such as free maternity services. However, questions remain around the timing of the first ANC visit: according to the WHO recommendations, the first visit is scheduled between week 8 and 12 of the pregnancy[31]. In our trial, the results show that women (in both arms) attend their visit in week 22 on average. Late attendance for ANC visits was also found in other studies[32, 37, 38], and the consequences of that late first visit could require further investigation, or further research could be done in how to incentivise early attendance.

The Nigerian CCT programme did not find any impacts on neonatal immunization[36]. In Zimbabwe, little improvement was found in immunization amongst children under 5 years of age[34]. This is in contrast with our findings. We did not observe a significant impact on facility delivery, which is consistent with the findings from[36] but not with other studies[18, 39] that found a positive impact of CCTs on facility delivery. We did not find a clear intervention effect on the proportion of women with at least one PNC visit at 4-12 months, but women did have higher numbers of total PNC visits in the intervention clinics. This suggests further research into unpacking why financial incentives do not have as consistent an impact on facility delivery and PNC visits or child immunisation in comparison to the effect on ANC service utilization.

We faced two major and connected challenges related to the technical functioning of the card system and a delay in the transfer of payments, the latter being common for CCT programmes[40]. The touching in of the Afya card reader was intended to record the visit and automatically trigger payments to participants. However, only 26% of payments were triggered automatically (further details in[27]). The remaining transfers required involvement of the field implementation partner to manually record a visit and trigger a transfer. This caused several delays in payments being made to participants, often over months. Other challenges such as healthcare staff not tapping the cards to avoid conflicts with participants over delayed payments, reluctance of new staff at facilities to participate due to challenges with delayed transfers; the card reader being locked by the main staff member actively involved in intervention to avoid theft but limiting use by other healthcare staff contributed to the intervention not being implemented as intended. All these factors, linked to the technology and delay in payments could have diluted the impact of the intervention and prevented service utilization in the latter stages of pregnancy or after babies were born.

Our findings of modest increases in scheduled ANC and immunisation appointment attendance, need to be interpreted in the context of the implementation challenges described, noting that most of these are partly inherent to the intervention we evaluated given the resources available and the trial

For peer review only - http://bmjopen.bmj.com/site/about/guidelines.xhtml

context. Although implementation of the intervention could have been enhanced for example by either more frequent regular visits to facilities to support clinic staff or by incentivising clinic staff more to process payments throughout a woman's care, these models may be unsustainable at scale. We saw evidence of a modest intervention effect on counts of all attendance types other than delivery when lifting the eligibility requirements for the analysis and thereby potentially including some unscheduled visits and visits additional to the expected maximum required. These findings may reflect in part an increase in visits made without serious health concerns where the cash transfer is a major motivation, though unscheduled visits were not eligible for payments. We conducted a per-protocol sensitivity analysis, but this was ultimately unhelpful since it led to an unfair comparison between arms, and so we are unable to quantify the intervention effects that might be expected had implementation been more complete.

The collection of outcome data as originally planned was unfeasible and, whilst it is a real strength that we managed to collect ANC attendance data for almost all trial participants, it is a limitation that we managed to collect data on the other primary outcomes for only a minority of women and less commonly in the control arm. The potential bias is however limited to a degree by our approach to analysis in which all outcomes are modelled simultaneously, in which we effectively use the ANC attendance data to predict (i.e., 'impute') the other outcomes. Although the telephone follow-up surveys proved challenging these data are not central to the analyses and interpretation presented here. We did not recruit to our original sample size target but obtained outcomes from all clusters.

Conclusions

This trial has demonstrated modest benefits from a CCT intervention, that was affected by technical and other implementation challenges. Further research is needed to address how to design a more robust process for registering attendances and ensuring rapid payment of CCTs to ensure women have confidence in receipt of CCTs for future attendances. This could impact incentivise women to attend visits earlier in their pregnancy as well.

Author contribution

FV was the Principal Investigator, leading study implementation from May 2018 to June 2020. OS, TP, and AC were the trial statisticians, with AC leading the design of the data analysis methods and interpretation of the research findings, and OS undertaking the analysis. AO was the Trial Coordinator, leading field implementation under supervision of AM. SD, JS, NB, HHB, TP led specific components of the trial such as the process evaluation and the economic evaluation and contributed to the overall research methods and design. The entire team contributed to the interpretation of the research findings and the writing of the paper. All authors read and approved the final manuscript.

Funding statements

The authors thank the Bill and Melinda Gates Foundation for the funding for this project (grant number: OPP 1142564) and the Swedish International Development Cooperation Agency for providing co-financing (grant number: not applicable). The funders were not involved in the design, implementation, data collection, analysis, writing and decision to submit the paper for publication.

Contributor statements

The authors thank the following people for their contribution to the trial: Caroline Ochieng, Alie Eleveld, Geordan Shannon, Stacey Noel, Matthew Fielding, Sangoro Onyango and Sarah Odera.

For peer review only - http://bmjopen.bmj.com/site/about/guidelines.xhtml

Competing interest

The authors declare no competing interests, aside from AC who is associate editor of Sexually Transmitted Infections.

Data Availability Statement

De-identified data may be made available upon request to researchers who provide a scientifically and methodologically sound research proposal and obtain ethical approval for their planned analysis. Proposals should be submitted to the corresponding author.

Figure 1 legend

n values refer to women and n_c to clinics. EDD expected date of delivery, ADD actual date of delivery.

References

1. WHO. TRENDS IN MATERNAL MORTALITY: 2000 TO 2017. Estimates by WHO, UNICEF, UNFPA, World Bank Group and the United Nations Population Division [Internet]. Geneva; 2019. Available from: https://www.unfpa.org/sites/default/files/pub-pdf/Maternal_mortality_report.pdf

2. Kenya National Bureau of Statistics. Kenya Demographic and Health Survey 2014 - Key Indicators [Internet]. Nairobi, Kenya; 2015 Mar. Available from: https://www.dhsprogram.com/pubs/pdf/sr227/sr227.pdf

3. UNICEF. Neo-natal mortality and under-five mortality [Internet]. 2020 Sep. Available from: https://data.unicef.org/topic/child-survival/under-five-mortality/#:~:text=Sub%2DSaharan%20Africa%20continues%20to,deaths%20per%201%2C000%20live%20births.

4. Say L, Chou D, Gemmill A, Tunçalp Ö, Moller A-B, Daniels J, et al. Global causes of maternal death: a WHO systematic analysis. Lancet Glob Health. 2014 Jun;2(6):e323–33.

5. UN IGME. Levels & Trends in Child Mortality. Report 2018 [Internet]. UNICEF; 2018. Available from: https://childmortality.org/reports

6. Campbell OM, Graham WJ. Strategies for reducing maternal mortality: getting on with what works. The Lancet. 2006 Oct;368(9543):1284–99.

7. Carroli G, Rooney C, Villar J. How effective is antenatal care in preventing maternal mortality and serious morbidity? An overview of the evidence. Paediatr Perinat Epidemiol. 2001 Jan;15(s1):1–42.

8. Barber SL, Gertler PJ. Empowering women: how Mexico's conditional cash transfer programme raised prenatal care quality and birth weight. J Dev Eff. 2010 Apr 13;2(1):51–73.

9. Okeke EN, Chari AV. Health care at birth and infant mortality: Evidence from nighttime deliveries in Nigeria. Soc Sci Med. 2018 Jan;196:86–95.

10. Daysal NM, Trandafir M, van Ewijk R. Saving Lives at Birth: The Impact of Home Births on Infant Outcomes. Am Econ J Appl Econ. 2015 Jul;7(3):28–50.

For peer review only - http://bmjopen.bmj.com/site/about/guidelines.xhtml

11. Warren C, Mwangi A, Oweya E, Kamunya R, Koskei N. Safeguarding maternal and newborn health: improving the quality of postnatal care in Kenya. Int J Qual Health Care. 2010 Feb 1;22(1):24–30.

12. Kerber KJ, de Graft-Johnson JE, Bhutta ZA, Okong P, Starrs A, Lawn JE. Continuum of care for maternal, newborn, and child health: from slogan to service delivery. The Lancet. 2007 Oct;370(9595):1358–69.

13. WHO. Factsheet Maternal Mortality [Internet]. 2018 Feb. Available from: https://www.who.int/news-room/fact-sheets/detail/maternal-mortality

14. Pell C, Meñaca A, Were F, Afrah NA, Chatio S, Manda-Taylor L, et al. Factors Affecting Antenatal Care Attendance: Results from Qualitative Studies in Ghana, Kenya and Malawi. PLOS ONE. 2013 Jan 15;8(1):e53747.

15. Kenya National Bureau of Statistics (KNBS), ICF Macro. Kenya Demographic and Health Survey 2008-09 [Internet]. Calverton, Maryland; 2010. Available from: https://dhsprogram.com/pubs/pdf/fr229/fr229.pdf

16. Lund S, Nielsen BB, Hemed M, Boas IM, Said A, Said K, et al. Mobile phones improve antenatal care attendance in Zanzibar: a cluster randomized controlled trial. BMC Pregnancy Childbirth. 2014 Jan 17;14(1):29.

17. Kebede AS, Ajayi IO, Arowojolu AO. Effect of enhanced reminders on postnatal clinic attendance in Addis Ababa, Ethiopia: a cluster randomized controlled trial. Glob Health Action. 2019 May 24;12(1):1609297.

18. Grépin KA, Habyarimana J, Jack W. Cash on delivery: Results of a randomized experiment to promote maternal health care in Kenya. J Health Econ. 2019 May 1;65:15–30.

19. Okeke EN, Abubakar IS. Healthcare at the beginning of life and child survival: Evidence from a cash transfer experiment in Nigeria. J Dev Econ. 2020 Mar 1;143:102426.

20. Hurst TE, Semrau K, Patna M, Gawande A, Hirschhorn LR. Demand-side interventions for maternal care: evidence of more use, not better outcomes. BMC Pregnancy Childbirth. 2015 Nov 13;15:297.

21. Ekezie CC, Lamont K, Bhattacharya S. Are Cash Transfer Programs Effective in Improving Maternal and Child Health in Sub-Saharan Africa? A Systematic Review of Randomized Controlled Trials. J Glob Health Columbia Univ [Internet]. 2017 May 21 [cited 2020 Nov 27];7(1). Available from: https://journals.library.columbia.edu/index.php/jgh/article/view/5012

22. Glassman A, Duran D, Fleisher L, Singer D, Sturke R, Angeles G, et al. Impact of conditional cash transfers on maternal and newborn health. J Health Popul Nutr. 2013 Dec;31(4 SUPPL.2):S48–66.

23. Hunter BM, Harrison S, Portela A, Bick D. The effects of cash transfers and vouchers on the use and quality of maternity care services: A systematic review. PLOS ONE. 2017 Mar 22;12(3):e0173068.

For peer review only - http://bmjopen.bmj.com/site/about/guidelines.xhtml

24. Lagarde M, Haines A, Palmer N. Conditional cash transfers for improving uptake of health interventions in low- and middle-income countries: a systematic review. JAMA. 2007 Oct 24;298(16):1900–10.

25. Ochieng CA, Haghparast-Bidgoli H, Batura N, Odhiambo A, Shannon G, Copas A, et al. Conditional cash transfers to retain rural Kenyan women in the continuum of care during pregnancy, birth and the postnatal period: protocol for a cluster randomized controlled trial. Trials. 2019 Mar 1;20(1):152.

26. Fielding M, Ochieng C, Odero B. From paper to data: taking medical health records into the future. 2016;7.

27. Dickin S, Vanhuyse F, Odhiambo A, Stirrup O, Liera C, Copas A, et al. Afya health credit incentive: a process evaluation. forthcoming.

28. Craig P, Dieppe P, Macintyre S, Michie S, Nazareth I, Petticrew M. Developing and evaluating complex interventions [Internet]. London, UK: Medical Research Council; 2019. Available from: https://mrc.ukri.org/documents/pdf/complex-interventions-guidance/

29. Palmer T, Batura N, Skordis J, Stirrup O, Copas A, Dickin S, et al. Cost-effectiveness of conditional cash transfers to retain women in the continuum of care during pregnancy, birth and the postnatal period: an economic evaluation of the Afya trial in Kenya. In 2021.

30. Waithaka D, Kagwanja N, Nzinga J, Tsofa B, Leli H, Mataza C, et al. Prolonged health worker strikes in Kenya- perspectives and experiences of frontline health managers and local communities in Kilifi County. Int J Equity Health. 2020 Feb 10;19(1):23.

31. WHO. WHO recommendations on antenatal care for a positive pregnancy experience. WHO; 2016.

32. Sarker BK, Rahman M, Rahman T, Rahman T, Khalil JJ, Hasan M, et al. Status of the WHO recommended timing and frequency of antenatal care visits in Northern Bangladesh. PLOS ONE. 2020 Nov 5;15(11):e0241185.

33. Kahn C, Iraguha M, Baganizi M, Kolenic GE, Paccione GA, Tejani N. Cash Transfers to Increase Antenatal Care Utilization in Kisoro, Uganda: A Pilot Study. Afr J Reprod Health. 2015 Sep;19(3):144–50.

34. Robertson L, Mushati P, Eaton JW, Dumba L, Mavise G, Makoni J, et al. Effects of unconditional and conditional cash transfers on child health and development in Zimbabwe: a cluster-randomised trial. The Lancet. 2013 Apr 13;381(9874):1283–92.

35. Dennis ML, Benova L, Abuya T, Quartagno M, Bellows B, Campbell OMR. Initiation and continuity of maternal healthcare: examining the role of vouchers and user-fee removal on maternal health service use in Kenya. Health Policy Plan. 2019 Mar 1;34(2):120–31.

36. Okoli U, Morris L, Oshin A, Pate MA, Aigbe C, Muhammad A. Conditional cash transfer schemes in Nigeria: potential gains for maternal and child health service uptake in a national pilot programme. BMC Pregnancy Childbirth. 2014 Dec 12;14(1):408.

37. Gebresilassie B, Belete T, Tilahun W, Berhane B, Gebresilassie S. Timing of first antenatal care attendance and associated factors among pregnant women in public health institutions of

For peer review only - http://bmjopen.bmj.com/site/about/guidelines.xhtml

Axum town, Tigray, Ethiopia, 2017: a mixed design study. BMC Pregnancy Childbirth. 2019 Sep 18;19(1):340.

38. Jiwani SS, Amouzou-Aguirre A, Carvajal L, Chou D, Keita Y, Moran AC, et al. Timing and number of antenatal care contacts in low and middle-income countries: Analysis in the Countdown to 2030 priority countries. J Glob Health. 2020;10(1):010502.

39. Powell-Jackson T, Mazumdar S, Mills A. Financial incentives in health: New evidence from India's Janani Suraksha Yojana. J Health Econ. 2015 Sep 1;43:154–69.

40. NGEC. Participation of vulnerable populations in their own programme: the cash transfers in Kenya [Internet]. 2014. Available from: https://www.ngeckenya.org/Downloads/cash-transfer-programme-vulnerable-groups-kenya.pdf

For peer review only

For peer review only - http://bmjopen.bmj.com/site/about/guidelines.xhtml

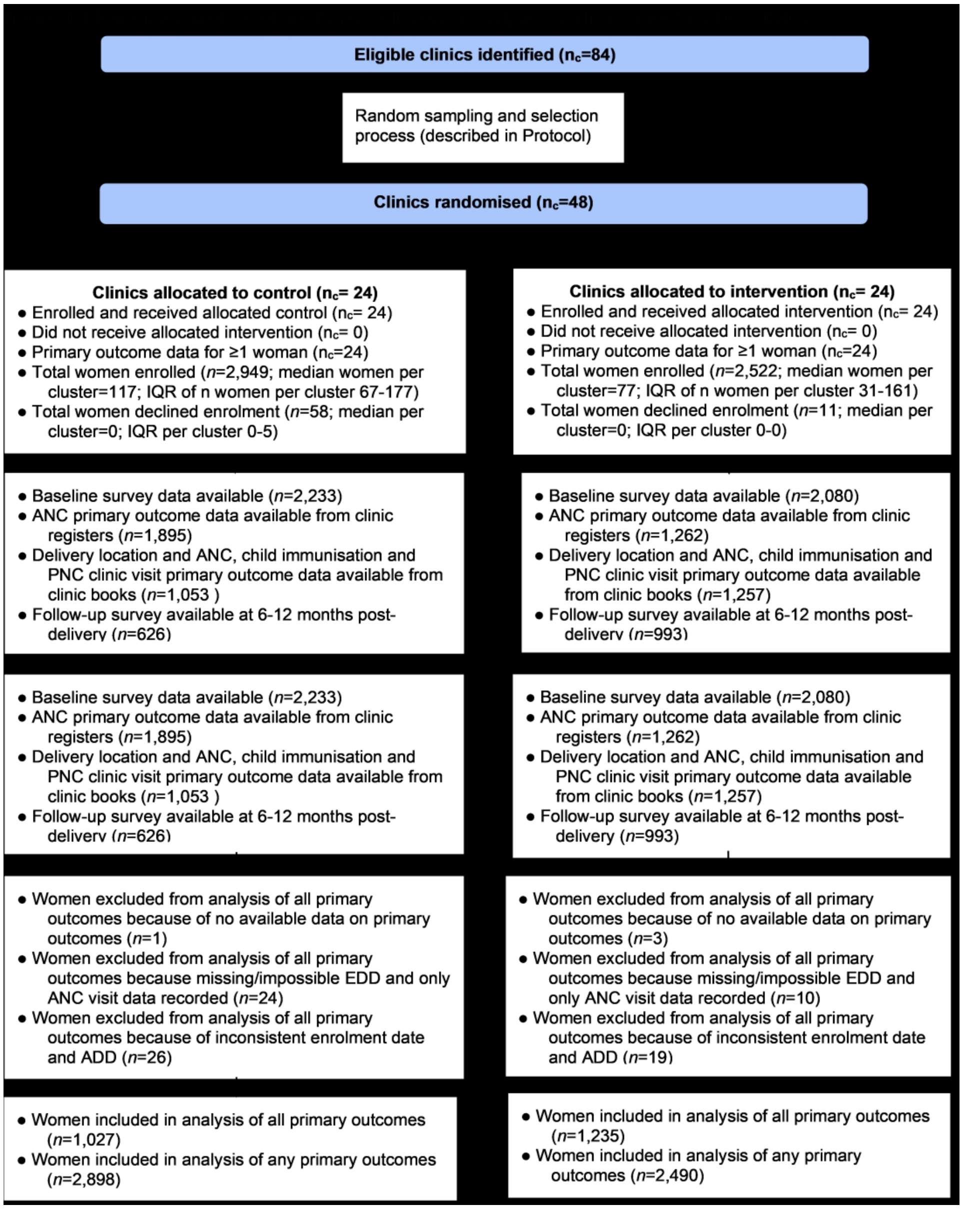

159x199mm (600 x 600 DPI)

Supplementary material

Appendix S1 – Differences from published protocol in data collection and analysis

This summary of differences between the trial protocol and the data collection and analysis that were conducted is closely based on that in the statistical analysis plan (SAP) for the study, which was written prior to unblinding and analysis of data by the trial statistician.

The published Protocol for the study stated that the primary outcomes for the study would be derived by combining data from manual extraction of clinic health records and from the electronic card reading system used to process the trial payments. However, due to technical issues in implementation of the card reading system, it was not possible to extract outcome data from this source. As such, outcome data were also obtained from the personal clinic books of women participating in the trial. Specifically, participants were invited to attend the clinic shortly after the end of the trial with their clinic book for collection of the primary outcomes. For women who however did not attend the clinic for that purpose, data were extracted from the clinic register. Only very limited data on any treatment referrals beyond routine visits were collected and as such this primary outcome (5 of 5 in the Protocol) was dropped from the planned analysis. In addition, there were only data available for the primary outcomes of delivery at a healthcare facility and child immunisation and maternal PNC visits for those women with clinic book available for data extraction (only antenatal visits were extracted from clinic registers).

It was planned that data for the secondary outcomes would be derived from clinic records, the electronic card reading system and a series of four follow-up telephone interviews (after enrolment in all women; 2 weeks after delivery in all women who do not give birth in a facility; 6 months after EDD in 50% of women; 12 months after delivery in the same 50% of women). However, as well as issues with the card reader system, the interviews at 6 and 12 months after delivery were combined into one survey which was primarily delivered towards 12 months after delivery, after some 6-month interviews had already been conducted. The following secondary outcomes were dropped as data were not collected: mother's perception of infant health at 6 and 12 months post-delivery, screening and control of infections for mothers and foetus/baby during pregnancy and postnatal periods.

Perinatal and maternal mortality were recorded if a woman (or family member) attended with their clinic book for data extraction, but completeness of the reporting has not been verified. The remaining outcomes were collected as part of the 6–12-month survey but the survey completion rate was ultimately relatively low, and as such, we have reported available data for the specified secondary outcomes at 6-12 months but have not carried out any formal statistical analyses. The completion rate of the baseline survey was high, but not sufficiently high to use these data to adjust our analyses for maternal characteristics such as socioeconomic factors.

Additional secondary outcomes of the counts of ANC, child immunisation and PNC clinic visits (both with and without applying any eligibility criteria regarding the timing of visits or maximum for each type) per woman were analysed and reported; this was not listed in the original Protocol, where the focus was on the primary outcomes of the *proportion* of each appointment type attended. Analysing the total counts in each case also allows for capture of the potential impact of earlier commencement of ANC care, and creates model outputs useful for health economic analysis. A further additional secondary outcome of GA at enrolment to the trial was also added, to evaluate whether the trial intervention encouraged earlier engagement with antenatal care (whereas the primary outcomes only relate to events following enrolment of each woman). This outcome was available for a large majority of women enrolled in the study.

For peer review only - http://bmjopen.bmj.com/site/about/guidelines.xhtml

The published Protocol stated that the analysis of primary outcomes would use logistic regression for binary outcomes and ordinal regression for ordinal outcomes, with a single pooled effect estimate for the intervention across these outcomes estimated using independence estimating equations. However, since delivery and postnatal outcome data were missing in a substantial proportion of participants, we planned to allow for dependence (i.e., correlation) between each of the outcome variables for each woman using structured random effects models. To facilitate this, visit counts for each woman were analysed as repeated binary observations rather than as ordinal variables.

Appendix S2 – Summary of available mortality data

These data have been obtained from both free-text notes in the study visit records and from information collected in the telephone surveys. It is therefore difficult to gauge the level of ascertainment of these adverse outcomes and the level of completeness will also depend on engagement with care and follow-up, which differed between the control and intervention groups.

Among the 2,949 women enrolled into the control arm, there was one record of intrauterine death of the foetus, one record of a stillbirth, 17 records of neonatal deaths (immediately or up to 1 week following delivery), 33 records of infant deaths up to 18 months after delivery and no records of maternal deaths.

Among the 2,522 women enrolled into the intervention arm, there were two records of intrauterine deaths of the foetus, three records of stillbirth, 23 records of neonatal deaths (immediately or up to 1 week following delivery), 45 records of infant deaths up to 18 months after delivery and two records of maternal deaths. The maternal deaths were both recorded at data extraction from maternal clinic books and appear to have occurred within a year of delivery, but not in the immediate neonatal period.

For peer review only

For peer review only - http://bmjopen.bmj.com/site/about/guidelines.xhtml

Additional Tables

Table S1: Cluster-level summaries of the enrolled women included in the primary analysis

Variable	Control clinic	Intervention clinic	ICC
N women per cluster in analysis	113 (67-174;21-301)	77 (31-158, 17-313)	—
**Baseline characteristics**			
Median age	26 (25-27, 22-29)	26 (25-27, 23-30)	—
Proportion nulliparous	0.22 (0.16-0.28, 0.004-0.37)	0.19 (0.10-0.26, 0-0.38)	—
Median GA at enrolment (days)	158 (151-166, 136-179)	153 (149-159, 139-183)	—
Proportion high-risk pregnancies	0.20 (0.11-0.24, 0.06-1)	0.17 (0.14-0.38, 0.06-0.94)	—
**Primary outcomes**			
Mean proportion attendance at eligible ANC clinic appointments (following scheduled visits)	0.61 (0.54-0.67, 0.48-0.75)	0.70 (0.65-0.75, 0.50-0.82)	0.028*
Proportion delivery at health facility	0.92 (0.89-0.96, 0.83-1)	0.90 (0.86-0.94,0.71-1)	0.012†
Proportion attendance eligible PNC clinic appointment (at least one 4-12mo)	0.85 (0.76-0.90, 0.49-1)	0.85 (0.77-0.90, 0.47-0.94)	0.087†
Mean proportion attendance at child immunisation appointments (capped at 4)	0.85 (0.79-0.90, 0.71-1)	0.89 (0.86-0.91, 0.76-0.98)	0.011*

Data shown as median (interquartile range, range) by cluster.
ICC values calculated as $(var(b_i))/(var(b_i)+var(e_{ij}))$ from a linear mixed model with adjustment for intervention and subcounty, where $var(b_i)$ is cluster-level random intercept variance and $var(e_{ij})$ is the residual variance. *Linear mixed model fitted to overall proportion of visits attended as outcome for each woman. †Linear mixed model fitted to binary outcome data (i.e., 0 for no visit and 1 for attendance).

For peer review only

Table S2: Effect of conditional cash transfers on primary outcome measures for the sensitivity analysis only including the 12 intervention clinics and the 12 control clinics with the highest proportion of enrolled women with payment within 31 days of first ANC clinic visit.

	**Control clinic**	**Intervention clinic**		
**Primary outcome measures**	***n/N*** **(%)**	***n/N*** **(%)**	**OR (95% CI)**	**aOR (95% CI);** ***P***
Attendance at eligible ANC clinic appointments (following scheduled visits)*	3491/5335 (65)	3720/5775 (64)	0.78 (0.55-1.11)	0.80 (0.55-1.17); *P*=0.25
Delivery at health facility†	575/624 (92)	754/838 (90)	0.31 (0.11-0.88)	0.32 (0.11-0.92); *P*=0.04
Attendance at eligible PNC clinic appointment (at least one 4-12mo)‡	503/624 (81)	680/835 (81)	0.88 (0.45-1.74)	0.92 (0.47-1.82); *P*=0.81
Attendance at child immunisation appointments (capped at 4)†	2109/2496 (84)	2960/3352 (88)	1.65 (0.89-3.05)	1.72 (0.93-3.18); *P*=0.08
Pooled intervention estimate	—	—	0.89 (0.65-1.22)	0.92 (0.66-1.27); *P*=0.60

aOR, adjusted odds ratio; OR, odds ratio. *Available for all women included in primary analysis except three (with data from clinic book but no available EDD or ADD). †Available for 1462/3279 women with data obtained from clinic books. ‡Available for 1459 women with data obtained from clinic books and with available ADD or EDD. Available data for attendances and eligible appointments are summed over all women for the *n*/N values.

For peer review only - http://bmjopen.bmj.com/site/about/guidelines.xhtml

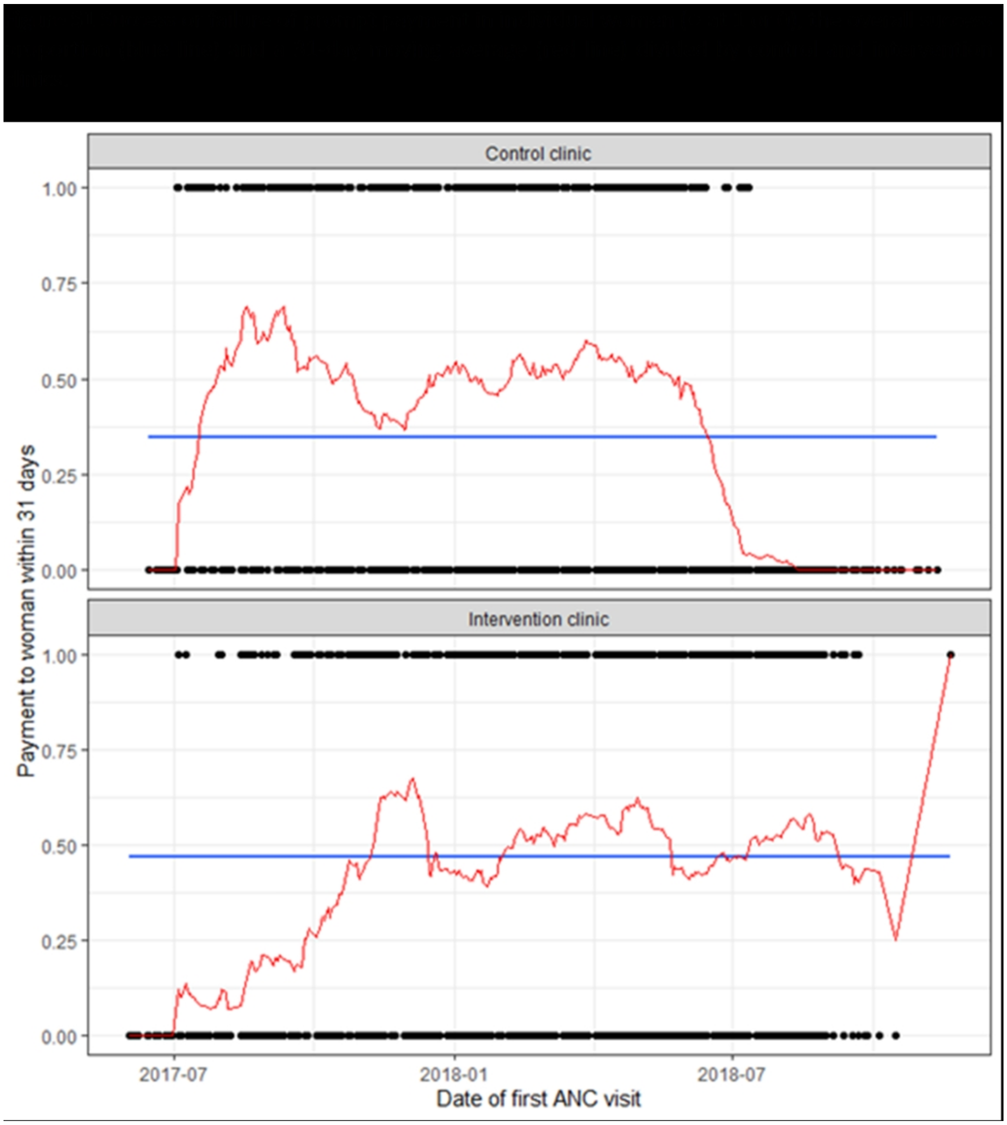

159x177mm (600 x 600 DPI)

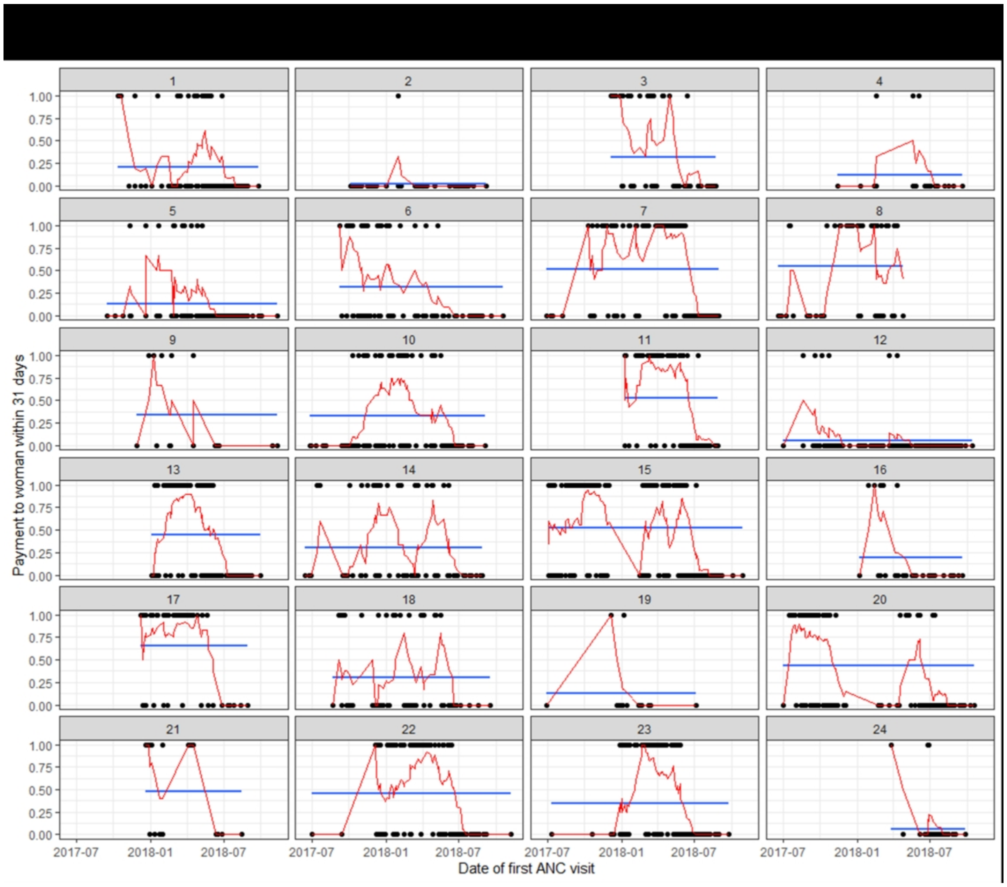

159x139mm (600 x 600 DPI)

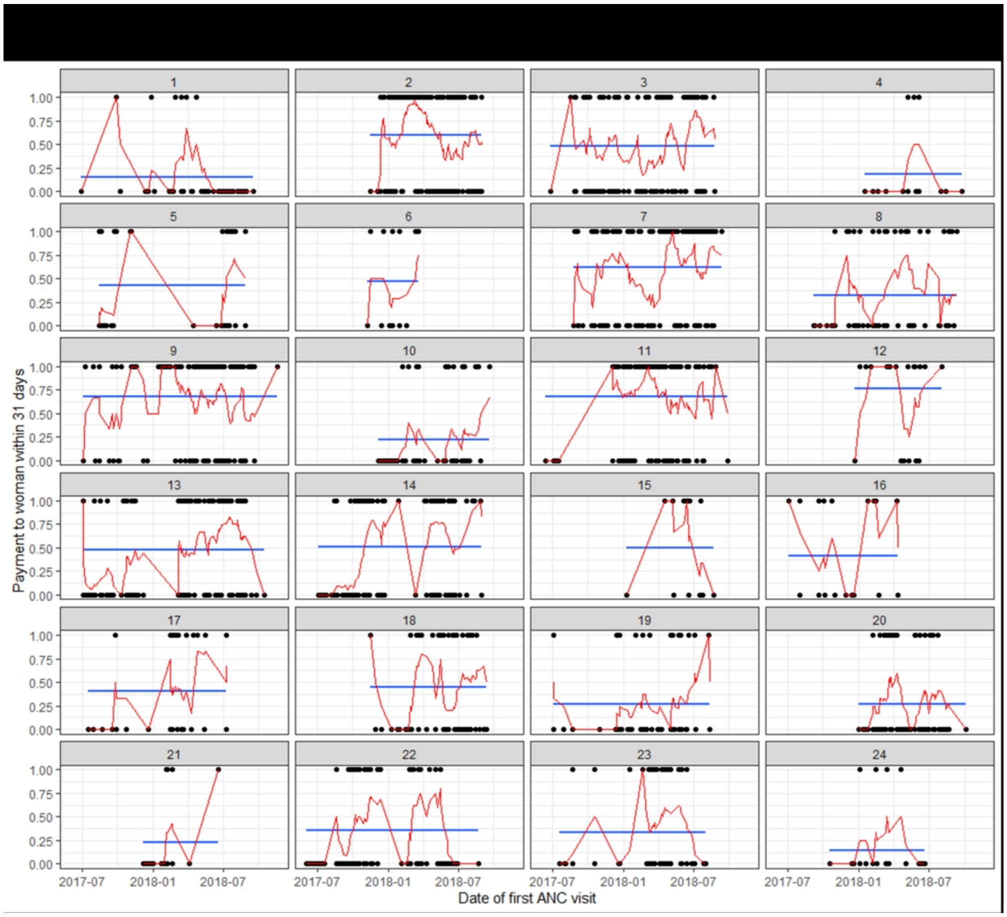

159x144mm (600 x 600 DPI)

[redacted]

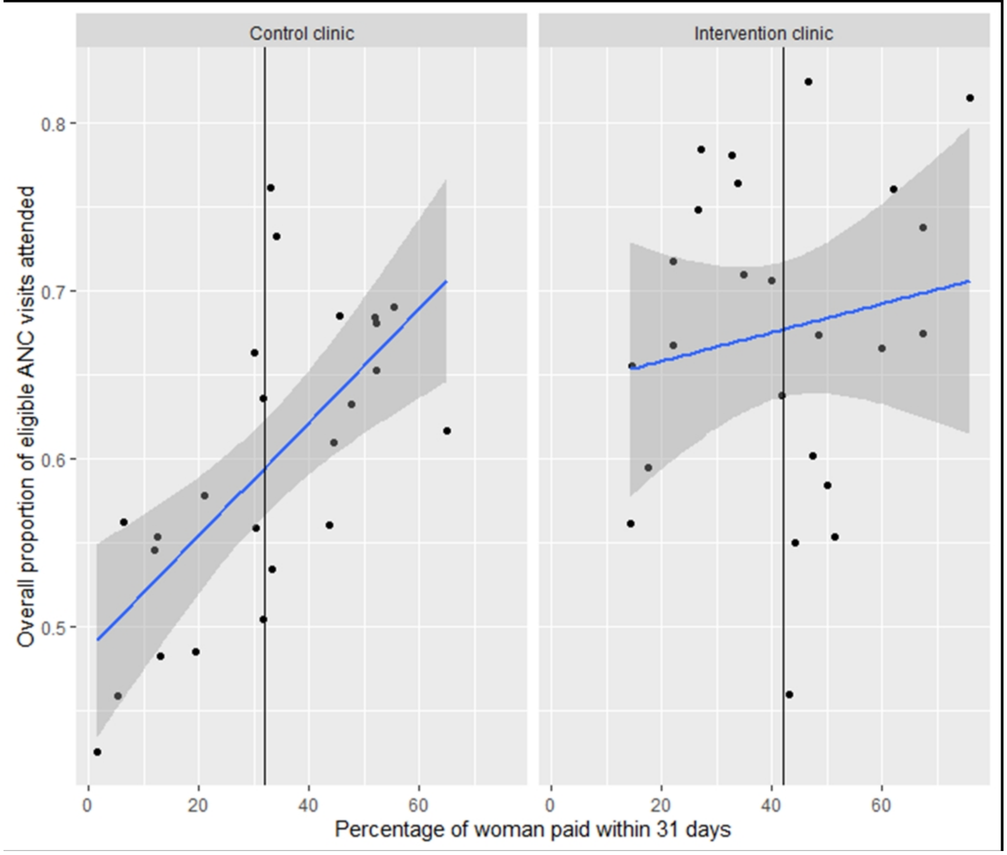

159x163mm (600 x 600 DPI)

CONSORT 2010 checklist of information to include when reporting a randomised trial*

Section/Topic	Item No	Checklist item	Reported on page No
**Title and abstract**			
	1a	Identification as a randomised trial in the title	2
	1b	Structured summary of trial design, methods, results, and conclusions (for specific guidance see CONSORT for abstracts)	2
**Introduction**			
Background and objectives	2a	Scientific background and explanation of rationale	4
	2b	Specific objectives or hypotheses	4
**Methods**			
Trial design	3a	Description of trial design (such as parallel, factorial) including allocation ratio	5
	3b	Important changes to methods after trial commencement (such as eligibility criteria), with reasons	Supplementary material p.1
Participants	4a	Eligibility criteria for participants	5
	4b	Settings and locations where the data were collected	5
Interventions	5	The interventions for each group with sufficient details to allow replication, including how and when they were actually administered	5
Outcomes	6a	Completely defined pre-specified primary and secondary outcome measures, including how and when they were assessed	5-6
	6b	Any changes to trial outcomes after the trial commenced, with reasons	6
Sample size	7a	How sample size was determined	6-7
	7b	When applicable, explanation of any interim analyses and stopping guidelines	8
Randomisation:			
Sequence generation	8a	Method used to generate the random allocation sequence	5
	8b	Type of randomisation; details of any restriction (such as blocking and block size)	5
Allocation concealment mechanism	9	Mechanism used to implement the random allocation sequence (such as sequentially numbered containers), describing any steps taken to conceal the sequence until interventions were assigned	5
Implementation	10	Who generated the random allocation sequence, who enrolled participants, and who assigned participants to interventions	5

For peer review only

Blinding	11a	If done, who was blinded after assignment to interventions (for example, participants, care providers, those assessing outcomes) and how	
	11b	If relevant, description of the similarity of interventions	
Statistical methods	12a	Statistical methods used to compare groups for primary and secondary outcomes	7-8
	12b	Methods for additional analyses, such as subgroup analyses and adjusted analyses	7-8; 13-14
**Results**			
Participant flow (a diagram is strongly recommended)	13a	For each group, the numbers of participants who were randomly assigned, received intended treatment, and were analysed for the primary outcome	8-9
	13b	For each group, losses and exclusions after randomisation, together with reasons	8-9
Recruitment	14a	Dates defining the periods of recruitment and follow-up	8
	14b	Why the trial ended or was stopped	8
Baseline data	15	A table showing baseline demographic and clinical characteristics for each group	10-11
Numbers analysed	16	For each group, number of participants (denominator) included in each analysis and whether the analysis was by original assigned groups	9
Outcomes and estimation	17a	For each primary and secondary outcome, results for each group, and the estimated effect size and its precision (such as 95% confidence interval)	11-12
	17b	For binary outcomes, presentation of both absolute and relative effect sizes is recommended	11-12
Ancillary analyses	18	Results of any other analyses performed, including subgroup analyses and adjusted analyses, distinguishing pre-specified from exploratory	13-14
Harms	19	All important harms or unintended effects in each group (for specific guidance see CONSORT for harms)	13-14
**Discussion**			
Limitations	20	Trial limitations, addressing sources of potential bias, imprecision, and, if relevant, multiplicity of analyses	14-15
Generalisability	21	Generalisability (external validity, applicability) of the trial findings	14-15
Interpretation	22	Interpretation consistent with results, balancing benefits and harms, and considering other relevant evidence	14-15
**Other information**			5
Registration	23	Registration number and name of trial registry	
Protocol	24	Where the full trial protocol can be accessed, if available	4
Funding	25	Sources of funding and other support (such as supply of drugs), role of funders	15

*We strongly recommend reading this statement in conjunction with the CONSORT 2010 Explanation and Elaboration for important clarifications on all the items. If relevant, we also recommend reading CONSORT extensions for cluster randomised trials, non-inferiority and equivalence trials, non-pharmacological treatments, herbal interventions, and pragmatic trials. Additional extensions are forthcoming: for those and for up to date references relevant to this checklist, see www.consort-statement.org.

For peer review only

For peer review only - http://bmjopen.bmj.com/site/about/guidelines.xhtml

Fedra Vanhuyse
Stockholm Environment Institute
Stockholm, Sweden

27 July 2021

Dear Adrian,

We hereby submit a manuscript entitled "Conditional cash transfers to retain women in the continuum of care in Kenya: Evaluating the Afya credits incentive for improved maternal and child health" for consideration in the journal BMJ Open as an original research article. We confirm that this work is original and has not been published elsewhere, nor is it currently under consideration for publication elsewhere.

This paper is, to our knowledge, one of the few trials that have assessed conditional cash transfers in the continuum of care (antenatal care visits, delivery at facility, postnatal care visits and immunization). It was carried out in Kenya from 2017 to 2019, and the results show that, despite challenges with the payment system (electronic transfers) and nurses' strikes, the conditional cash transfers had a positive effect on antenatal care visits and immunization. No intervention effect was seen for delivery at facility and postnatal attendance. Another finding was that there was little difference in the mean gestational age at enrolment of women in the intervention arm and the control arm (week 22-23), or ten - fourteen weeks after the first antenatal visit recommended by the WHO.

We selected your journal given its focus on global health issues, policy, and multidisciplinary research, which is at the core of our paper and research. Our word count is however slightly over the 4,000 words, as we feel all sections in our paper contain valuable contributions.

We have no conflicts of interest to disclose, aside from Andrew Copas who is associate editor of Sexually Transmitted Infections. All authors agree with its submission to the journal BMJ Open an original research article.

Please address all correspondence concerning this manuscript to me at fedra.vanhuyse@sei.org.

Thank you for your consideration of this manuscript.

Kind regards,

Fedra Vanhuyse

For peer review only

BMJ Open

Effectiveness of conditional cash transfers (Afya credits incentive) to retain women in the continuum of care during pregnancy, birth and the postnatal period in Kenya: a cluster randomised trial

Journal:	*BMJ Open*
Manuscript ID	bmjopen-2021-055921.R1
Article Type:	Original research
Date Submitted by the Author:	08-Nov-2021

Complete List of Authors:	Vanhuyse, Fedra; Stockholm Environment Institute,<br>Stirrup , Oliver ; University College London, Institute for Global Health<br>Odhiambo, Aloyce; Safe Water and AIDS Project<br>Palmer, Tom; University College London, Institute for Global Health<br>Dickin, Sarah; Stockholm Environment Institute<br>Skordis-Worrall, Jolene; UCL, Institute for Global Health<br>Batura, Neha; University College London, Institute for Global Health<br>Haghparast-Bidgoli, Hassan; University College London, Institute for Global Health<br>Mwaki, Alex; Safe Water and Aids Project (SWAP)<br>Copas, Andrew; University College London, Institute for Global Health

**Primary Subject Heading**:	Global health
Secondary Subject Heading:	Health services research
Keywords:	PUBLIC HEALTH, Community child health < PAEDIATRICS, Maternal medicine < OBSTETRICS

SCHOLARONE™ Manuscripts

I, the Submitting Author has the right to grant and does grant on behalf of all authors of the Work (as defined in the below author licence), an exclusive licence and/or a non-exclusive licence for contributions from authors who are: i) UK Crown employees; ii) where BMJ has agreed a CC-BY licence shall apply, and/or iii) in accordance with the terms applicable for US Federal Government officers or employees acting as part of their official duties; on a worldwide, perpetual, irrevocable, royalty-free basis to BMJ Publishing Group Ltd ("BMJ") its licensees and where the relevant Journal is co-owned by BMJ to the co-owners of the Journal, to publish the Work in this journal and any other BMJ products and to exploit all rights, as set out in our licence.

The Submitting Author accepts and understands that any supply made under these terms is made by BMJ to the Submitting Author unless you are acting as an employee on behalf of your employer or a postgraduate student of an affiliated institution which is paying any applicable article publishing charge ("APC") for Open Access articles. Where the Submitting Author wishes to make the Work available on an Open Access basis (and intends to pay the relevant APC), the terms of reuse of such Open Access shall be governed by a Creative Commons licence – details of these licences and which Creative Commons licence will apply to this Work are set out in our licence referred to above.

Other than as permitted in any relevant BMJ Author's Self Archiving Policies, I confirm this Work has not been accepted for publication elsewhere, is not being considered for publication elsewhere and does not duplicate material already published. I confirm all authors consent to publication of this Work and authorise the granting of this licence.

For peer review only

Title: Effectiveness of conditional cash transfers (Afya credits incentive) to retain women in the continuum of care during pregnancy, birth and the postnatal period in Kenya: a cluster randomised trial

Corresponding author: Fedra Vanhuyse, Stockholm Environment Institute, Linnégatan 87D, Box 24218, 104 51 Stockholm, Sweden.

Email: fedra.vanhuyse@sei.org

Authors: Fedra Vanhuyse*[1], Oliver Stirrup[2], Aloyce Odhiambo[3], Tom Palmer[2], Sarah Dickin[1], Jolene Skordis[2], Neha Batura[2], Hassan Haghparast-Bidgoli[2], Alex Mwaki[3] and Andrew Copas[2]

[1] Stockholm Environment Institute, Linnégatan 87D, Box 24218, 104 51 Stockholm, Sweden.

[2] Institute for Global Health, University College London, London, UK.

[3] Safe Water and AIDS Project (SWAP), P.O. Box 3323, Kisumu 40100, Kenya.

Key words: public health, community child health, maternal medicine

Word count: 4,840

For peer review only - http://bmjopen.bmj.com/site/about/guidelines.xhtml

Abstract

Objectives

Given high maternal and child mortality rates, we assessed the impact of Conditional Cash Transfers (CCTs) to retain women in the continuum of care (antenatal care (ANC), delivery at facility, postnatal care (PNC) and child immunization).

Design

We conducted an unblinded 1:1 cluster-randomized controlled trial.

Setting

48 health facilities in Siaya County, Kenya were randomized.

Participants

2922 women were recruited to the control and 2522 to the intervention arm.

Interventions

An electronic system recorded attendance and triggered payments to the participant's mobile for the intervention arm (4.5 USD), and phone credit for the control arm (0.5 USD). Eligibility criteria were resident in the catchment area and access to a mobile phone.

Primary outcomes

Primary outcomes were any ANC, delivery, any PNC between 4- 12 months after delivery, childhood immunization, and referral attendance to other facilities for ANC or PNC. Given problems with the electronic system, primary outcomes were obtained from maternal clinic books if participants brought them to data extraction meetings (1257 (50%) of intervention and 1053 (36%) control arm participants).

Results

We found a significantly higher proportion of appointments attended for ANC (67% vs. 60%, adjusted OR (aOR) 1.90; 95% CI 1.36-2.66) and child immunization (88 vs 85%; aOR 1.74; 95% CI 1.10-2.77) in intervention than control arm. No intervention effect was seen considering delivery at the facility (90 vs 92%; aOR 0.58; 95% CI 0.25-1.33) and any PNC attendance (82 vs 81%; aOR 1.25; 95% CI 0.74-2.10) separately. The pooled odds ratio across all attendance types was 1.64 (1.28-2.10).

Conclusions

Demand-side financing incentives, such as CCTs, can improve attendance for appointments. However, attention needs to be paid to the technology, the barriers that remain for delivery at facility and PNC visits and encouraging women to attend ANC visits within the recommended WHO timeframe.

Trial registration

NCT03021070; clinicalTrials.gov

For peer review only

Strengths and limitations of this study

- Technical issues with the electronic system and at times low participation of health workers resulted in many visits not being registered, and only 26% of the payments triggered automatically.
- Manual payments needed to be triggered, which resulted in delays.
- This delay in payment could have diluted the impact of the intervention and prevented service utilization in the latter stages of pregnancy or after babies were born.
- Manual data abstraction from clinic registers and from the women's maternal clinic books was necessary. This resulted in near complete data for ANC attendance but limited data on facility delivery, PNC and child immunization and no data on referral attendance.
- The potential bias is however limited to a degree by our approach to analysis in which all outcomes are modelled simultaneously.

For peer review only - http://bmjopen.bmj.com/site/about/guidelines.xhtml

Introduction

Every year an estimated 295,000 maternal deaths occur globally, with 99% occurring in low and middle-income countries, and almost two thirds in Africa[1]. In Sub-Sahara Africa, the maternal mortality rate was 542 deaths per 100,000 live births in 2017[1]. In Kenya, the maternal mortality rate in 2014 was 362 deaths per 100,000 live births[2], categorized as "high" according to the World Health Organization (WHO). Globally, an estimated 2.4 million infants die in the first month of life (40% of all deaths) and 1.6 million at age 1–11 months (25%)[3]. Sub-Saharan Africa has the highest neonatal mortality rate at a median of 27 deaths per 1,000 live births and the highest under-five mortality rate, with a median of 76 deaths per 1,000 live births[3]. In Kenya, the median infant mortality rate was 21 deaths per 1,000 live births and the median under-five mortality rate was 43 deaths per 1,000 live births[2], which are amongst the highest in the region.

Most maternal and neonatal deaths are avertible through the use of healthcare interventions that prevent or manage pregnancy-related complications such as postpartum haemorrhage and infectious diseases[4,5]. The importance of health service utilisation for maternal and child health outcomes has been extensively documented, including for antenatal care (ANC)[6–8], facility delivery[9, 10], postnatal care (PNC)[11] ,and across the continuum of care[12]. In Kenya, while 96% of women receive some form of ANC, less than three in five receive the four ANC visits that the WHO recommends[2]. Only one in five have their first ANC visit during the first trimester as recommended by the WHO[2], which would allow to monitor the health of both mother and child more effectively. Almost four in ten babies are delivered at home, and 62% of new-borns do not receive a PNC check-up in the first week after birth[2]. Almost half (47%) of mothers do not receive a PNC check-up in the first two days after birth[2]. Lower attendance at healthcare facilities could be due to a lack of skilled health workers, poverty, distance, lack of information, inadequate services and cultural practices[13]. Other research[14] reported costs charged for ANC visits, nurses' behaviour, and the timing of the visits as the main barriers for attending ANC visits. In Kenya, a survey showed that financial barriers (costs of care for other children, food, new clothes), and lack of transport and distance to health care facilities were the main barriers[15].

Aside from programmes to improve the quality and reach of the service (supply side interventions), demand side financing interventions have been set up to incentivise women to attend visits. Examples of such interventions include mobile phone text message reminders for ANC visits in Zanzibar[16] and for PNC visits in Tanzania[17], as well as the use of conditional cash transfers (CCTs). In Kenya, a conditional cash transfer intervention in Vihiga County was found to increase facility delivery by 7.9 percentage points[18]. A recent study in Nigeria[19] found that payments for retention from ANC to PNC resulted in more women attending the visits (26% of women in the intervention arm compared to 12% in the control arm), leading to a 22% reduction in the stillbirth rate. Recent systematic reviews on the demand-side interventions for maternal care[20] and on CCTs[21–24] found increased utilisation of services, but not always better outcomes.

The Afya trial aims to test the effectiveness of a conditional cash transfer to retain women in the continuum of care, from their first ANC visit until their children reach 1 year of age in Siaya County, Kenya. The CCT aimed at tackling multiple barriers to care, as described in the trial's protocol[25]: women would receive equal-sized cash transfers following a visit (ANC, delivery at facility, PNC visit and childhood immunization), as well as a reminder for their visit by text message and medical staff would be trained in the technology and incentivised for each woman they enrolled in the trial. Unlike a study in Nigeria[19], in Afya, the CCT was done through a card reader system rather than cash and, additionally, it initiated transfers for each individual visit, rather than being conditional on receiving an entire package of care. In this paper, we present the results of the impact of the Afya trial.

For peer review only - http://bmjopen.bmj.com/site/about/guidelines.xhtml

Methods

Study setting, design and randomisation

We conducted a cluster randomized controlled trial, with equal allocation to intervention and control arms, in Siaya county, Kenya. The units of randomisation were Level 2 or 3 health facilities (Dispensaries and Health Centres, respectively). The randomisation of facilities was stratified by the six sub-Counties and ensured equal allocation to study arms within each stratum without any overlap of catchment areas,, as described in detail in the trial protocol[25]. In summary, at a public forum with the county government early 2016, the implementing partner wrote the names of 60 shortlisted facilities on pieces of paper and folded them to hide the names, then included them in transparent boxes, one for each sub-county. Each subcounty had an (even) number of facilities to recruit to the trial proportional to subcounty size. The health management teams from each subcounty selected the pieces of paper, one by one. The first was allocated to intervention, second to control. For each selected facility, county officials from the selected subcounty mapped the location and catchment area of the facility on a large map of the county. If a subsequently selected facility had an overlapping catchment area with a previously selected facility, the newly selected facility was rejected, and another drawn to take its place. This process continued until 48 facilities were selected and allocated for the trial.

Health facility staff determined whether a pregnant woman met the study eligibility criteria by administering screening questions at the end of her first ANC visit, with the screening questions provided in the trial protocol [25]. All women meeting the criteria were eligible for recruitment during the study recruitment period. Criteria for enrolment were women attending their first ANC visit; long-term resident of the catchment area served by the health facility (living in the area for at least 6 months); access to a mobile phone that belongs either to themselves or to a member of their household or person whom they trust. The criterium on residence provided additional assurance that women went to the facility within their catchment area, thereby reducing contamination with other facilities. Oral informed consent was asked in the local language, and then written down on the participant's enrolment form. Refusals were recorded.

Intervention

The intervention was a CCT payment for each facility appointment attended for ANC, delivery, postnatal care, and childhood immunization. Detailed definitions can be found in Appendix S1. For each scheduled health visit made following enrolment, women in the intervention arm received a cash transfer of KSH 450 (4.5 USD) on their mobile phones. Women at the control clinics were granted KSH 50 (0.5 USD) mobile phone airtime for each scheduled visit to encourage them to bring their clinic booklet to appointments. In both trial arms, women were issued with a trial card at recruitment and at all facilities there was a card reader, which provided the connection between the trial card and an online portal which stored participants' data on visits and payments. Payments to the women were triggered by tapping the card on a card reader, which also logged the visit in an online portal[26]. In the event of problems with the card reader, or if the woman did not bring her card to the appointment, payments could alternatively be processed manually by contacting the implementing partner: once the visit was verified with the facility, the implementing partner entered the visit data in the portal, which would then trigger a payment as well Nurses were given KSH 400 (4 USD) per woman enrolled during the trial, and an additional KSH 100 (1USD) per woman enrolled at the end of the trial for their collaboration in the trial. These payments were transferred to the nurses electronically. Details of the intervention design are presented in the protocol[25].

For peer review only - http://bmjopen.bmj.com/site/about/guidelines.xhtml

Patient and Public involvement statement

Patients or the public were not involved in the design, conduct, reporting, or dissemination plans of the research. The assessment of the burden of the intervention on patients is reported in[27].

Ethics statement

Ethical approval was granted by Maseno University in Kenya (MSU/DRP/MUERC/00294/16).

Trial outcomes

The primary outcomes were: 1) attendance or missed attendance at each eligible ANC appointment after recruitment; 2) delivery at a health facility; 3) attendance for at least one PNC appointment between 4 and 12 months after delivery; 4) attendance or missed attendance at each expected child immunization appointment; 5) attendance at referrals to other facilities for ANC, PNC or child immunization. We define 'eligible visits' for the purpose of statistical analysis of the impact of the intervention within a clearly defined framework, but all scheduled pre- and post-natal clinic visits should have triggered a payment. The PNC attendance outcome described in the protocol was the 'proportion of required postnatal visits honoured after recruitment into the study'. However, we have used a simplified outcome here because the required appointment schedule was not recorded for each patient. We also restricted attendances to the period 4-12 months after delivery because on blinded review of the available data, prior to writing the statistical analysis plan (SAP), very few visits prior to 4 months post-delivery were coded as PNC. The vast majority of visits prior to 4 months were recorded as vaccination appointments. though it is likely some women also received PNC. Vaccinations over the 12 months after delivery were recorded. The details of vaccinations given were not collected, and so the vaccination outcome is based purely on the number of recorded visits from an expected number of four.

The following secondary outcomes are also reported and analysed according to trial arm: 1) attendance of all eligible maternal, new-born and child health (MNCH) care visits, both prenatal and postnatal, for each woman; 2) the count of attended ANC and child immunisation clinic visits eligible for the primary outcome variables for each woman; 3) the total number of ANC, child immunisation and PNC clinic visits (without applying any eligibility criteria) for each woman; 4) gestational age (GA) at first ANC visit (and enrolment to study). The secondary outcomes of clinic visit counts and GA at first ANC visit were not listed in the trial protocol but were prespecified in the SAP to aid interpretation of the study results.

The primary outcome of attendance at referrals to other facilities for ANC, PNC or child immunization is not reported because of very limited data. The following planned secondary outcomes are reported without formal statistical analysis because of low levels of data completeness: maternal and neonatal mortality, self-rated wellness, exclusive breastfeeding, and contraceptive use. The following secondary outcomes were dropped because of lack of available data: timeliness of health visits (recorded visits could not consistently be matched up to scheduled dates), and infection screening. These changes were specified in the SAP. Appendix S1 contains further details of changes made to the data collection and analysis compared to the protocol, arising from data collection challenges.

Data collection

Data were collected throughout the trial from an electronic card reading system, and baseline and follow-up surveys. The electronic card reading system captured the enrolled women's phone number, expected delivery date (EDD), parity, the clinic she enrolled at, the visits she attended and the payments she received. The baseline survey, carried out by telephone following enrolment, collected socio-demographic data. We initially planned to conduct follow-up interviews with 50% of enrolled

For peer review only - http://bmjopen.bmj.com/site/about/guidelines.xhtml

women at 6 and 12 months post-delivery to collect secondary outcomes. However, due to limited resources and lower than anticipated response rates after some 6-month interviews had been conducted, we adopted a pragmatic approach and conducted one follow-up survey at around 12 months after delivery.

Problems with the implementation of the technical system and periods of disengagement from clinic staff resulted in a large proportion of visits not being registered on the system. Therefore, data on women's visits were manually extracted from clinic health records after the trial was completed. Trial staff visited each facility, after arranging for trial participants to be invited to attend the facility with their maternal clinic book. Data on the primary outcomes of ANC, child immunisation and PNC visits and delivery at a healthcare facility were extracted from maternal clinic books. The books very rarely contained records of any referral visits. For women who did not attend the data extraction, data on the primary outcome of ANC visits were extracted from health facility registers, but data on other visits were not available from this source. Any missing payments were transferred to the participating women following the manual data extraction at the end of the trial.

Data on payments made to trial participants, whether triggered by the card reader or manually, were extracted from the trial portal. These data are reported in the process evaluation paper, along with details of the challenges with the electronic system[27].

Sample size

We analysed all primary outcomes jointly, to maximise power, aid interpretation and minimise testing (see Statistical Analysis). However, for our sample size calculation, we considered the power to detect an effect of the intervention on one primary outcome, which we also assumed to be a binary indicator that all attendances were made, as this is simple and conservative in the power achieved. The expected prevalence of these indicators in the control arm ranged between 30 and 80%. In the absence of specific information on the likely intra cluster correlation (ICC) we considered a range between 0.005 (low) and 0.025 (moderate). Our planned sample size was 48 clusters covering the catchment areas of selected level 2 and 3 health facilities (24 per arm) and an average cluster size of 150 participants. At a low ICC, the design effect (DE) would be 1.745 and hence the effective sample size (ESS) would be 2,063 participants per study arm. At a moderate ICC, the DE would be 4.725 and hence the ESS 762 per arm. Power to detect absolute differences is lowest when the prevalence is 50% and highest when the prevalence is either high (towards 100%) or low (towards 0%). Here we considered the prevalence in the control arm to range between 50% ('worst-case scenario') and 80% ('best-case scenario'). We considered the standard 5% significance level. If the prevalence of the outcome is 50% in the control arm, the sample size provides 80% power to detect an improvement to 54.5% in the intervention arm if the ICC is low and 57.5% if the ICC is moderate. If the prevalence of the outcome is 80% in the control arm, the sample size provides 80% power to detect an improvement to 83.5% if the ICC is low and 85.5% if the ICC is moderate.

Data definitions and processing

For the purpose of statistical analysis, eligible ANC visits were defined based on the recorded 'next scheduled visit date' and noting that this was typically 4 weeks later each time. For each recorded visit starting with enrolment, we evaluated whether the next observed ANC visit was within ±2 weeks of the 'next scheduled visit date': recording a successful ANC visit if yes, but a missed visit if not. However, we did not count the visit as either successful or missed if the next observed visit was more than 2 weeks early compared to the next scheduled visit or delivery occurred within 2 weeks of a 'missed' scheduled visit. Whether that next visit is early, on time, or late, we assessed subsequent visit attendances in the same way. We created hypothetical scheduled visits every 4 weeks for any gaps in

For peer review only - http://bmjopen.bmj.com/site/about/guidelines.xhtml

observations, judged according to the same criteria. The 'successful' and 'missed' appointments were then summed over all scheduled and hypothetical appointments for each woman.

For the primary outcomes of PNC visits, we defined a binary indicator of one or more PNC visit 4-12 months postpartum. This was used to provide a simple indicator of engagement with postnatal care for each woman, given that the appropriate number of PNC visits may differ between women. We defined child immunisation visits as the total number of visits recorded post-partum (excluding vaccination at delivery, but without other time restrictions) truncated at a maximum of four, since that is the typical number required for full immunisation.

Retention in the full continuum of antenatal, perinatal, and postnatal care (a secondary outcome) was defined as a binary indicator of attendance of all eligible ANC, child immunisation and PNC visits and delivery at a healthcare facility for each woman and is available for those women who brought their clinic book for data extraction.

Statistical analysis

The primary outcomes of attendance of eligible ANC and child immunisation appointments comprise repeat binary observations for each individual woman, whilst the primary outcomes of delivery at a health facility and PNC attendance between 4–12 months are each single binary variables for each woman. A summary odds ratio is presented as the main effect measure for the trial, estimated from a model that assumes the odds ratio is the same across the four primary outcomes. We also report separate effect estimates for each primary outcome. A mixed effects logistic regression model was used to jointly analyse the observed primary outcomes. This approach assumes that, given their ANC attendances (recorded for nearly all women), whether a woman did or didn't bring their clinic book for extraction of the other outcomes was unrelated to the values of those outcomes. At the level of each woman correlated random effects were specified for (1) attendance of ANC clinic visits and (2) grouped outcomes of delivery at a healthcare facility, attendance of vaccination visits and attendance of at least one PNC clinic visit at 4–12 months post-partum. Clinic-level random effect terms were defined for (1) ANC visits, (2) delivery at a healthcare facility and (3) PNC and child immunisation visits, with unrestricted correlations between these.

The secondary outcomes of counts of ANC clinic, PNC clinic and child immunisation visits were analysed using a multivariate Poisson mixed effects model, with random intercept terms at patient and the clinic levels. Marginal mean differences in counts between intervention groups were estimated. The secondary outcome of GA at enrolment to the trial was analysed using a linear mixed effects model, with random intercept term at clinic level. Retention in the full continuum of care was analysed using a logistic regression mixed effects model, with random intercept term at clinic level. As the completion rate of the follow-up survey was lower than expected, the secondary outcome data obtained is reported in a descriptive summary but not compared between trial arms.

Our main analyses are conducted as randomised (i.e., intention to treat) but a 'per-protocol' style sensitivity analysis of the primary outcomes was also prespecified. As there was not a clear division between clinics that did and did not achieve the intended payment schedule, the sensitivity analysis included the 12 intervention clinics and the 12 control clinics with the highest proportion of enrolled women with payment within 31 days of first ANC clinic visit.

All analyses were adjusted by the baseline maternal parity ('0' vs '≥1'), by the presence of any maternal medical conditions leading to classification of the pregnancy as high-risk (HIV with or without ART, diabetes, hypertension, malaria, each coded with separate indicator variables) and by the clinic-level variable of sub-county. Adjusted effect measures are considered the primary effect measures though unadjusted effect estimates are also reported. The analysis followed a pre-specified statistical analysis

For peer review only - http://bmjopen.bmj.com/site/about/guidelines.xhtml

plan, which was finalised after data collection but prior to any unblinded analysis. Selection of maternal characteristics as adjustment variables was based on their inclusion in the core enrolment dataset, the associated absence of missing data for these items and their potential to predict the outcomes of interest. Sub-county was included as an adjustment variable because of its use as a stratification factor in the randomisation process for the study.

As recommended by the MRC framework for the evaluation of complex interventions[28], a detailed process and economic evaluation will be published in two forthcoming papers[27, 29].

Results

The trial was conducted in 24 intervention and 24 control clinics and enrolled a total of 2522 women at intervention clinics and 2949 at control clinics over a period from May 2017 to December 2019 (Figure 1). Only 11 eligible women declined enrolment at intervention clinics and 58 at control clinics. Based on the background data on ANC attendance in the study region in 2015, it was expected that each of the 48 facilities would recruit 150 participants into the trial, meeting the target sample size of 7,200 eligible women during the trial period. However, enrolment stopped before the target sample size of 7,200 could be reached due to delays arising from the nurses' strike during which enrolment was paused at many clinics (see, e.g.,[30] who discussed the strike and its impacts on health care delivery), and as the trial was intended to run until 2018 initially. The vast majority (5,388 or 98.5%) of women had data on ANC attendances, but data from maternal clinic books on all primary outcomes were available in a minority of women (2,262/5,388, 42.0%). Socio-demographic characteristics for all enrolled women are presented in Table 1, with cluster-level summaries in Table S1. Baseline survey data were available in 4,313/5,471 (78.8%) women, and a summary of selected fields by arm is presented in Table 2. Table 1 and 2 demonstrate very good balance between arms.

Table 1: Baseline and pregnancy characteristics of the enrolled women included in the primary analysis, obtained from enrolment data, clinic book and clinic registry data

**Variable**		**Control clinic, *n* (%) or median (IQR)**	**Intervention clinic, *n* (%) or median (IQR)**
**Baseline characteristics**			
Age	Years (median (IQR) [*n*])	26 (22-31) [2738]	26 (22-31) [2349]
Parity	0	606 (21)	488 (20)
	1	698 (24)	591 (24)
	2	587 (20)	485 (19)
	≥3	1007 (35)	926 (37)
GA at enrolment	Weeks+days (median (IQR) [*n*])	22+4 (17+4 to 27+2) [2898]	22+3 (17+2 to 27+0) [2487]
HIV status	Negative	2481 (86)	2142 (86)
	Positive, on treatment	398 (14)	322 (13)
	Positive, not on treatment	19 (1)	26 (1)
Diabetes	No	2889 (99.7)	2482(99.7)
	Yes	9 (0.3)	8 (0.3)
Hypertension	No	2892 (99.8)	2485 (99.8)
	Yes	6 (0.2)	5 (0.2)
Malaria	No	2603 (90)	2164 (87)
	Yes	295 (10)	326 (13)
Total high-risk	No	2245 (77)	1850 (74)

For peer review only - http://bmjopen.bmj.com/site/about/guidelines.xhtml

pregnancies	Yes	653 (23)	640 (26)
**Pregnancy Characteristics**			
GA at delivery	Weeks+days (median (IQR) [*n*])	39+3 (37+0 to 41+1) [1004]	39+0 (36+4 to 41+0) [1206]

IQR, interquartile range. GA gestational age

Table 2: Sociodemographic characteristics of the enrolled women from baseline survey

**Variable**		**Control clinic, *n* (%)**	**Intervention clinic, *n* (%)**
Enrolled women	Total *n*	2949	2522
Baseline survey	Available	2233 (76)	2080 (82)
	Missing	716 (24)	442 (18)
Self-rated maternal health	Very good	4 (0.2)	3 (0.1)
	Good	771 (35)	718 (35)
	Moderate	1445 (65)	1351 (65)
	Bad	12 (0.5)	6 (0.3)
	Very Bad	0 (0)	0 (0)
	Missing*	1 (0.04)	2 (0.1)
Maternal education level	None or only literacy	15 (0.7)	17 (0.8)
	Primary incomplete	631 (28)	540 (26)
	Primary complete	860 (39)	796 (38)
	Secondary incomplete	352 (16)	343 (16)
	Secondary complete	292 (13)	292 (14)
	University/college	79 (4)	91 (4)
	Don’t know/other/missing*	4 (0.2)	1 (0.05)
Mode of travel to facility for enrolment visit	Public transport, e.g. bus	727 (33)	718 (35)
	Mini bus taxi	0 (0)	0 (0)
	Metered /taxi	4 (0.2)	7 (0.3)
	Walking	1485 (67)	1341 (64)
	Car	1 (0.04)	1 (0.05)
	Other	16 (0.7)	11 (0.5)
	Missing*	0 (0)	2 (0.1)
Travel time to facility for enrolment visit	<1 hour	1444 (65)	1314 (63)
	1-2 hours	741 (33)	699 (34)
	2-3 hours	43 (2)	60 (3)
	>3 hours	4 (0.2)	5 (0.2)
	Missing*	1 (0.04)	2 (0.1)

*Of those women with baseline survey recorded.

Primary and secondary outcomes

The proportion of eligible ANC appointments attended was significantly higher in the intervention arm compared to control (67 vs. 60%; aOR 1.90; 95% CI 1.36-2.66). A smaller increase was also demonstrated in the proportion of eligible immunisation appointments attended (88 vs 85%; aOR 1.74; 95% CI 1.10-2.77). For the other primary outcomes reporting was similar between arms (Table 3). The pooled aOR for the intervention effect giving a summary measure across all primary outcomes was 1.64 (95%CI 1.28-2.10), p<0.001. The intervention effect on the number of eligible ANC attendances, expressed as an adjusted marginal change (Table 3), was an increase of 0.31 (0.15 to 0.47). The adjusted marginal change in eligible immunisation attendances was not significant (0.14, -

For peer review only - http://bmjopen.bmj.com/site/about/guidelines.xhtml

0.12 to 0.41). Increases in attendances were seen for all visit types when the eligibility requirements defined for the primary analysis were removed, thereby considering all healthcare visits (including any unscheduled visits). The intervention had no effect on the timing of first ANC visit, the mean GA at enrolment was 22.2 weeks for intervention and 22.3 weeks for control, or quite a few weeks after the recommended first visit by the WHO[31] but consistent with other studies in low- and middle-income countries[32]. Postnatal surveys at 5-18 months after delivery were completed by a minority of women, selected outcomes are reported by arm in Table 4. Maternal and perinatal mortality were not systematically recorded, but the available data on these outcomes are summarised in Appendix S2. The ICC was 0.028 for the primary outcome of ANC visits, 0.012 for delivery at a health facility, 0.087 for attendance of at least one eligible PNC visit and 0.011 for immunisation visits.

Table 3: Effect of conditional cash transfers on primary and secondary outcome measures

	**Control clinic**	**Intervention clinic**		
**Primary outcome measures**	***n/N* (%)**	***n/N* (%)**	**OR (95% CI)**	**aOR (95% CI); *P***
Attendance at eligible ANC clinic appointments (following scheduled visits)*	5,827/9,736 (60)	5,741/8,595 (67)	1.95 (1.39-2.72)	1.90 (1.36-2.66); *P*<0.001
Delivery at health facility†	945/1,027 (92)	1,115/1,238 (90)	0.59 (0.26-1.35)	0.58 (0.25-1.33); *P*=0.20
Attendance at one or more eligible PNC clinic appointment (4-12 months after delivery)‡	831/1,027 (81)	1,016/1,235 (82)	1.26 (0.75-2.12)	1.25 (0.74-2.10); *P*=0.40
Attendance at child immunisation appointments (capped at 4)†	3,498/4,108 (85)	4,353/4,952 (88)	1.76 (1.10-2.80)	1.74 (1.10-2.77); *P*=0.02
Pooled intervention estimate	—	—	1.69 (1.32-2.17)	1.64 (1.28-2.10); *P*<0.001
**Secondary outcome measures**	**Control clinic**	**Intervention clinic**		
	***n/N* (%)**	***n/N* (%)**		**aOR (95% CI)**
Attendance at all eligible ANC and PNC visits, child immunisation appointments and delivery at a healthcare facility (per woman)	480/1,027 (47)	632/1,235 (51)		1.14 (0.82 to 1.57)
	Mean (95% CI) ¶, Median (IQR)	***Mean (95% CI) ¶, Median (IQR)***		**Average difference in mean (95% CI) ¶**
**Visit counts (eligible for primary outcome)**				
Total attendances at eligible ANC clinic appointments (following scheduled visits)*	2.04 (1.93-2.14) 2 (0-3)	2.34 (2.22-2.47), 2 (1-3)		0.31 (0.15 to 0.47)
Total attendances at eligible child immunisation appointments (capped at 4)†	3.33 (3.14-3.52), 4 (3-4)	3.48 (3.29-3.67), 4 (4-4)		0.14 (-0.12 to 0.41)

For peer review only - http://bmjopen.bmj.com/site/about/guidelines.xhtml

Visit counts (no eligibility criteria applied)			
Total attendances at ANC clinic appointments‡	2.05 (1.93-2.18), 2 (0-3)	2.42 (2.27-2.57), 2 (1-4)	0.37 (0.18 to 0.56)
Total attendances at PNC clinic appointments†	4.17 (3.90-4.44), 4 (2-7)	4.76 (4.46-5.06), 5 (2-7)	0.58 (0.19 to 0.98)
Total attendances at child immunisation appointments†	3.64 (3.40-3.88), 4 (3-5)	4.00 (3.75-4.26), 4 (4-5)	0.36 (0.02 to 0.71)
	**Mean (95% CI)**	**Mean (95% CI)**	**aΔ (weeks) (95% CI)**
GA at enrolment (weeks)*	22.3 (21.9-22.7)	22.2 (21.8-22.6)	-0.1 (-0.6 to 0.5)

aΔ, adjusted difference in mean; aOR, adjusted odds ratio; OR, odds ratio.
*Available for all women included in primary analysis except three (with data from clinic book but no available EDD or ADD).
†Available for 2265/5388 women with data obtained from clinic books. ‡Available for all women included in primary analysis.
¶Marginal value derived from multivariate Poisson mixed effects model applied to visit counts, estimated over the baseline characteristics of all 5388 women with data for at least one of the primary outcomes.
‡Available for 2262 women with data obtained from clinic books and with available ADD or EDD. Available data for attendances and eligible appointments are summed over all women for the *n*/N values.

Table 4: Data from follow-up surveys completed 5-18 months after delivery

**Variable**		**Control clinic**	**Intervention clinic**
Enrolled women	Total *n*	2,949	2,522
Survey answers 5-18 months after delivery			
Survey	Available	626 (21)	993 (39)
	Missing	2,323 (79)	1,529 (61)
Time from delivery at survey (days)		379 (273-463)	375 (273-469)
Self-rated maternal health	Very good	70 (11)	185 (19)
	Good	144 (23)	190 (19)
	Moderate	37 (6)	42 (4)
	Bad	4 (0.6)	3 (0.3)
	Very Bad	0 (0)	0 (0)
	Not asked in survey included	359 (57)	548 (55)
	Missing*	12 (2)	25 (3)
Exclusive breastfeeding to 6 months	Yes	244 (39)	396 (40)
	No	11 (2)	24 (2)
	Not asked in survey included	359 (57)	548 (55)
	Missing*	12 (2)	25 (3)
Family planning advice at last clinic visit	Yes	539 (86)	864 (87)
	No	58 (9)	78 (8)
	Missing*	29 (5)	51 (5)
Current method to prevent pregnancy	None	94 (15)	137 (14)
	NA: pregnant	3 (0.5)	4 (0.4)
	Yes: contraceptive	433 (69)	723 (73)
	Yes: natural methods	57 (9)	49 (5)
	Yes: other	9 (1)	29 (3)
	Missing*	30 (5)	51 (5)

Data shown as *n*, *n* (%) or median (IQR). Data were available for some women for both the planned '6 month survey' and the planned '12 month survey' (although the actual timing was not necessarily as planned), and in these the latter was used if completed 5-18 months after delivery as it contained questions on self-rated maternal health and age at end of exclusive breastfeeding. *Of those women with relevant survey data available.

For peer review only - http://bmjopen.bmj.com/site/about/guidelines.xhtml

Sensitivity analysis

A sensitivity 'per protocol' analysis of the primary outcomes was prespecified to only include those clinics and periods for which payments were being processed. However, as the correct functioning of the payment systems did not follow clear temporal divisions, the sensitivity analysis was conducted including those intervention and control clinics with a proportion of women with prompt payment (within 31 days) of their first ANC visit above the median within each arm.

We evaluated prompt payment from first visit among the 4,156 women with at least one ANC visit included in the primary analysis. There was a prompt payment in 743/2,141 (34.7%) women in the control clinics and in 943/2,015 (46.8%) in the intervention clinics (Figure S1). Across control clinics, the median proportion with prompt payment for first visit was 32.3%, with interquartile range (IQR) 17.8% to 46.1% (Figure S2). Across intervention clinics, the median proportion with prompt payment was 42.4%, with IQR 27.0% to 50.4% (Figure S3).

In the sensitivity analysis, there was no observed positive effect of the implementation on ANC attendance, and the pooled estimate of the intervention effect was negative but not statistically significant (Table S2). As this finding was surprising given the main results, we investigated the cluster-level association between prompt payment and ANC attendance. This revealed a stronger correlation in the control clinics than in the intervention clinics (Figure S4). Consequently, in our sensitivity analysis in terms of ANC attendance rates, we were unexpectedly comparing high performing control clinics with typical intervention clinics, which explains the change to the intervention effect from the main analysis.

Discussion

In this paper, we evaluated the impact of a demand side financing intervention, using CCTs, on the retaining pregnant women in the continuum of care from their first ANC visit until their children reach 1 year of age in rural Kenya. Previous evaluations of CCT programmes in the Sub Saharan Africa region have focussed on either increasing ANC visits[33], institutional deliveries[18, 33] or PNC visits[34]. Two other studies in the region evaluated the impact of demand side financing on the retaining women in the continuum of care. For the continuum of care, one study evaluated the impact of a subsidized reproductive health voucher programme and the introduction of free maternity services in government facilities on ANC visits, facility birth and PNC visits in Kenya[35]. Another study[36] examined the impact of a national CCT pilot programme on the continuum of care in rural Nigeria. To our knowledge, this is the first evaluation of the impact of CCTs on retaining pregnant women in the continuum of care in Kenya and provides crucial evidence to inform policy and practice related to demand side financing mechanisms for improving maternal and new-born health outcomes.

Increased ANC clinic attendance and child immunization appointments

Our main finding suggests that the intervention led to a modest increase in ANC clinic attendance and child immunisation appointments. This is consistent with evidence on the impact of demand side financing programmes on ANC service utilization from the sub–Saharan African region but less so with the evidence on child immunization. For example, a study set in Kenya[35] found that a subsidized reproductive health voucher programme and free maternity services improved early initiation of ANC as well as continuous use of care amongst ANC attendees in government facilities. Others[33, 36] also found that CCT programmes increased ANC attendance in rural Kenya and Nigeria, respectively. This implies that demand side financing interventions, whether using CCTs or vouchers can increase ANC service utilization, though the size of the impact might vary. It is possible that the increase in ANC

For peer review only - http://bmjopen.bmj.com/site/about/guidelines.xhtml

service utilization might be greater if financial incentives such as CCTs and vouchers are combined with other policy measures such as free maternity services.

As this was a cluster RCT, it was possible that knowledge of the incentives spread in the community and women could have attend earlier to collect more incentives. However, the results show that women (in both arms) attended their visit in week 22 on average. According to the WHO recommendations, the first ANC visit should be scheduled between week 8 and 12 of the pregnancy[31]. Late attendance for ANC visits was also found in other studies[32, 37, 38], and the consequences of that late first visit require further investigation, and further research could be done in how to incentivise early attendance.

Limited effect on facility delivery and PNC visits

The Nigerian CCT programme did not find any impacts on neonatal immunization[36]. In Zimbabwe, little improvement was found in immunization amongst children under 5 years of age[34]. This is in contrast with our findings. We did not observe a significant impact on facility delivery, which is consistent with the findings from[36] but not with other studies[18, 39] that found a positive impact of CCTs on facility delivery. We did not find a clear intervention effect on the proportion of women with at least one PNC visit at 4-12 months, but women did have higher numbers of total PNC visits in the intervention clinics. This suggests further research into unpacking why financial incentives do not have as consistent an impact on facility delivery and PNC visits or child immunisation in comparison to the effect on ANC service utilization.

Challenges with the trial

We faced two major and connected challenges related to the technical functioning of the card system and a delay in the transfer of payments, the latter being common for CCT programmes[40]. The touching in of the Afya card reader was intended to record the visit and automatically trigger payments to participants. However, only 26% of payments were triggered automatically (further details in[27]). The remaining transfers required involvement of the field implementation partner to manually record a visit and trigger a transfer. This caused several delays in payments being made to participants, often over months. Other challenges such as healthcare staff not tapping the cards to avoid conflicts with participants over delayed payments, reluctance of new staff at facilities to participate due to challenges with delayed transfers; the card reader being locked by the main staff member actively involved in intervention to avoid theft but limiting use by other healthcare staff contributed to the intervention not being implemented as intended. All these factors, linked to the technology and delay in payments could have diluted the impact of the intervention and prevented service utilization in the latter stages of pregnancy or after babies were born.

Our findings of modest increases in scheduled ANC and immunisation appointment attendance, need to be interpreted in the context of the implementation challenges described, noting that most of these are partly inherent to the intervention we evaluated given the resources available and the trial context. Although implementation of the intervention could have been enhanced for example by either more frequent regular visits to facilities to support clinic staff or by incentivising clinic staff more to process payments throughout a woman's care, these models may be unsustainable at scale. We saw evidence of a modest intervention effect on counts of all attendance types other than delivery when lifting the eligibility requirements for the analysis and thereby potentially including some unscheduled visits and visits additional to the expected maximum required. These findings may reflect in part an increase in visits made without serious health concerns where the cash transfer is a major motivation, though unscheduled visits were not eligible for payments. We conducted a per-protocol sensitivity analysis, but this was ultimately unhelpful since it led to an unfair comparison between

For peer review only - http://bmjopen.bmj.com/site/about/guidelines.xhtml

arms, and so we are unable to quantify the intervention effects that might be expected had implementation been more complete.

Our randomisation process was based on selecting 48 facilities to participate from a shortlist of 60 and simultaneously randomising these to intervention or control. Where the catchment area for a selected facility was found to overlap with a previously selected facility it was replaced. Although we believe the randomisation process was implemented objectively, we acknowledge that there could have been some subjectivity in deciding whether catchment areas overlapped and since the allocation to intervention or control was already revealed at this point it is theoretically possible that bias was introduced.

The collection of outcome data as originally planned was unfeasible and, whilst it is a real strength that we managed to collect ANC attendance data for almost all trial participants, it is a limitation that we managed to collect data on the other primary outcomes for only a minority of women and less commonly in the control arm, and no data on attendance for referrals. The potential bias is however limited to a degree by our approach to analysis in which all outcomes are modelled simultaneously. Although the telephone follow-up surveys proved challenging these data are not central to the analyses and interpretation presented here. We did not recruit to our original sample size target but obtained outcomes from all clusters.

Conclusions

This trial has demonstrated modest benefits from a CCT intervention, that was affected by technical and other implementation challenges. Further research is needed to address how to design a more robust process for registering attendances and ensuring rapid payment of CCTs to ensure women have confidence in receipt of CCTs for future attendances. This could impact incentivise women to attend visits earlier in their pregnancy as well.

Author contribution

FV was the Principal Investigator, leading study implementation from May 2018 to June 2020. OS, TP, and AC were the trial statisticians, with AC leading the design of the data analysis methods and interpretation of the research findings, and OS undertaking the analysis. AO was the Trial Coordinator, leading field implementation under supervision of AM. SD, JS, NB, HHB, TP led specific components of the trial such as the process evaluation and the economic evaluation and contributed to the overall research methods and design. The entire team contributed to the interpretation of the research findings and the writing of the paper. All authors read and approved the final manuscript.

Funding statements

The authors thank the Bill and Melinda Gates Foundation for the funding for this project (grant number: OPP 1142564) and the Swedish International Development Cooperation Agency for providing co-financing (grant number: not applicable). The funders were not involved in the design, implementation, data collection, analysis, writing and decision to submit the paper for publication.

Acknowledgements

The authors thank the following people for their contribution to the trial: Caroline Ochieng, Alie Eleveld, Geordan Shannon, Stacey Noel, Matthew Fielding, Sangoro Onyango and Sarah Odera.

For peer review only - http://bmjopen.bmj.com/site/about/guidelines.xhtml

Competing interest

The authors declare no competing interests, aside from AC who is associate editor of Sexually Transmitted Infections.

Data Availability Statement

De-identified data may be made available upon request to researchers who provide a scientifically and methodologically sound research proposal and obtain ethical approval for their planned analysis. Proposals should be submitted to the corresponding author. Data that can be shared includes number of visits made, type of visit, arm, and date of the visit. Data dictionaries can be made available, as well as the study protocol and the statistical analysis plan. Data are available now.

Figure legend

Figure 1. Flow diagram of enrolment and inclusion in analyses by clinic randomization status. n values refer to women and n_c to clinics. EDD expected date of delivery, ADD actual date of delivery.

For peer review only

For peer review only - http://bmjopen.bmj.com/site/about/guidelines.xhtml

References

1. WHO. TRENDS IN MATERNAL MORTALITY: 2000 TO 2017. Estimates by WHO, UNICEF, UNFPA, World Bank Group and the United Nations Population Division [Internet]. Geneva; 2019. Available from: https://www.unfpa.org/sites/default/files/pub-pdf/Maternal_mortality_report.pdf

2. Kenya National Bureau of Statistics. Kenya Demographic and Health Survey 2014 - Key Indicators [Internet]. Nairobi, Kenya; 2015 Mar. Available from: https://www.dhsprogram.com/pubs/pdf/sr227/sr227.pdf

3. UNICEF. Neo-natal mortality and under-five mortality [Internet]. 2020 Sep. Available from: https://data.unicef.org/topic/child-survival/under-five-mortality/#:~:text=Sub%2DSaharan%20Africa%20continues%20to,deaths%20per%201%2C000%20live%20births.

4. Say L, Chou D, Gemmill A, Tunçalp Ö, Moller A-B, Daniels J, et al. Global causes of maternal death: a WHO systematic analysis. Lancet Glob Health. 2014 Jun;2(6):e323–33.

5. UN IGME. Levels & Trends in Child Mortality. Report 2018 [Internet]. UNICEF; 2018. Available from: https://childmortality.org/reports

6. Campbell OM, Graham WJ. Strategies for reducing maternal mortality: getting on with what works. The Lancet. 2006 Oct;368(9543):1284–99.

7. Carroli G, Rooney C, Villar J. How effective is antenatal care in preventing maternal mortality and serious morbidity? An overview of the evidence. Paediatr Perinat Epidemiol. 2001 Jan;15(s1):1–42.

8. Barber SL, Gertler PJ. Empowering women: how Mexico's conditional cash transfer programme raised prenatal care quality and birth weight. J Dev Eff. 2010 Apr 13;2(1):51–73.

9. Okeke EN, Chari AV. Health care at birth and infant mortality: Evidence from nighttime deliveries in Nigeria. Soc Sci Med. 2018 Jan;196:86–95.

10. Daysal NM, Trandafir M, van Ewijk R. Saving Lives at Birth: The Impact of Home Births on Infant Outcomes. Am Econ J Appl Econ. 2015 Jul;7(3):28–50.

11. Warren C, Mwangi A, Oweya E, Kamunya R, Koskei N. Safeguarding maternal and newborn health: improving the quality of postnatal care in Kenya. Int J Qual Health Care. 2010 Feb 1;22(1):24–30.

12. Kerber KJ, de Graft-Johnson JE, Bhutta ZA, Okong P, Starrs A, Lawn JE. Continuum of care for maternal, newborn, and child health: from slogan to service delivery. The Lancet. 2007 Oct;370(9595):1358–69.

13. WHO. Factsheet Maternal Mortality [Internet]. 2018 Feb. Available from: https://www.who.int/news-room/fact-sheets/detail/maternal-mortality

14. Pell C, Meñaca A, Were F, Afrah NA, Chatio S, Manda-Taylor L, et al. Factors Affecting Antenatal Care Attendance: Results from Qualitative Studies in Ghana, Kenya and Malawi. PLOS ONE. 2013 Jan 15;8(1):e53747.

For peer review only - http://bmjopen.bmj.com/site/about/guidelines.xhtml

15. Kenya National Bureau of Statistics (KNBS), ICF Macro. Kenya Demographic and Health Survey 2008-09 [Internet]. Calverton, Maryland; 2010. Available from: https://dhsprogram.com/pubs/pdf/fr229/fr229.pdf

16. Lund S, Nielsen BB, Hemed M, Boas IM, Said A, Said K, et al. Mobile phones improve antenatal care attendance in Zanzibar: a cluster randomized controlled trial. BMC Pregnancy Childbirth. 2014 Jan 17;14(1):29.

17. Kebede AS, Ajayi IO, Arowojolu AO. Effect of enhanced reminders on postnatal clinic attendance in Addis Ababa, Ethiopia: a cluster randomized controlled trial. Glob Health Action. 2019 May 24;12(1):1609297.

18. Grépin KA, Habyarimana J, Jack W. Cash on delivery: Results of a randomized experiment to promote maternal health care in Kenya. J Health Econ. 2019 May 1;65:15–30.

19. Okeke EN, Abubakar IS. Healthcare at the beginning of life and child survival: Evidence from a cash transfer experiment in Nigeria. J Dev Econ. 2020 Mar 1;143:102426.

20. Hurst TE, Semrau K, Patna M, Gawande A, Hirschhorn LR. Demand-side interventions for maternal care: evidence of more use, not better outcomes. BMC Pregnancy Childbirth. 2015 Nov 13;15:297.

21. Ekezie CC, Lamont K, Bhattacharya S. Are Cash Transfer Programs Effective in Improving Maternal and Child Health in Sub-Saharan Africa? A Systematic Review of Randomized Controlled Trials. J Glob Health Columbia Univ [Internet]. 2017 May 21 [cited 2020 Nov 27];7(1). Available from: https://journals.library.columbia.edu/index.php/jgh/article/view/5012

22. Glassman A, Duran D, Fleisher L, Singer D, Sturke R, Angeles G, et al. Impact of conditional cash transfers on maternal and newborn health. J Health Popul Nutr. 2013 Dec;31(4 SUPPL.2):S48–66.

23. Hunter BM, Harrison S, Portela A, Bick D. The effects of cash transfers and vouchers on the use and quality of maternity care services: A systematic review. PLOS ONE. 2017 Mar 22;12(3):e0173068.

24. Lagarde M, Haines A, Palmer N. Conditional cash transfers for improving uptake of health interventions in low- and middle-income countries: a systematic review. JAMA. 2007 Oct 24;298(16):1900–10.

25. Ochieng CA, Haghparast-Bidgoli H, Batura N, Odhiambo A, Shannon G, Copas A, et al. Conditional cash transfers to retain rural Kenyan women in the continuum of care during pregnancy, birth and the postnatal period: protocol for a cluster randomized controlled trial. Trials. 2019 Mar 1;20(1):152.

26. Fielding M, Ochieng C, Odero B. From paper to data: taking medical health records into the future. 2016;7.

27. Dickin S, Vanhuyse F, Odhiambo A, Stirrup O, Liera C, Copas A, et al. Afya health credit incentive: a process evaluation. forthcoming.

For peer review only

28. Craig P, Dieppe P, Macintyre S, Michie S, Nazareth I, Petticrew M. Developing and evaluating complex interventions [Internet]. London, UK: Medical Research Council; 2019. Available from: https://mrc.ukri.org/documents/pdf/complex-interventions-guidance/

29. Palmer T, Batura N, Skordis J, Stirrup O, Copas A, Dickin S, et al. Cost-effectiveness of conditional cash transfers to retain women in the continuum of care during pregnancy, birth and the postnatal period: an economic evaluation of the Afya trial in Kenya. In 2021.

30. Waithaka D, Kagwanja N, Nzinga J, Tsofa B, Leli H, Mataza C, et al. Prolonged health worker strikes in Kenya- perspectives and experiences of frontline health managers and local communities in Kilifi County. Int J Equity Health. 2020 Feb 10;19(1):23.

31. WHO. WHO recommendations on antenatal care for a positive pregnancy experience. WHO; 2016.

32. Sarker BK, Rahman M, Rahman T, Rahman T, Khalil JJ, Hasan M, et al. Status of the WHO recommended timing and frequency of antenatal care visits in Northern Bangladesh. PLOS ONE. 2020 Nov 5;15(11):e0241185.

33. Kahn C, Iraguha M, Baganizi M, Kolenic GE, Paccione GA, Tejani N. Cash Transfers to Increase Antenatal Care Utilization in Kisoro, Uganda: A Pilot Study. Afr J Reprod Health. 2015 Sep;19(3):144–50.

34. Robertson L, Mushati P, Eaton JW, Dumba L, Mavise G, Makoni J, et al. Effects of unconditional and conditional cash transfers on child health and development in Zimbabwe: a cluster-randomised trial. The Lancet. 2013 Apr 13;381(9874):1283–92.

35. Dennis ML, Benova L, Abuya T, Quartagno M, Bellows B, Campbell OMR. Initiation and continuity of maternal healthcare: examining the role of vouchers and user-fee removal on maternal health service use in Kenya. Health Policy Plan. 2019 Mar 1;34(2):120–31.

36. Okoli U, Morris L, Oshin A, Pate MA, Aigbe C, Muhammad A. Conditional cash transfer schemes in Nigeria: potential gains for maternal and child health service uptake in a national pilot programme. BMC Pregnancy Childbirth. 2014 Dec 12;14(1):408.

37. Gebresilassie B, Belete T, Tilahun W, Berhane B, Gebresilassie S. Timing of first antenatal care attendance and associated factors among pregnant women in public health institutions of Axum town, Tigray, Ethiopia, 2017: a mixed design study. BMC Pregnancy Childbirth. 2019 Sep 18;19(1):340.

38. Jiwani SS, Amouzou-Aguirre A, Carvajal L, Chou D, Keita Y, Moran AC, et al. Timing and number of antenatal care contacts in low and middle-income countries: Analysis in the Countdown to 2030 priority countries. J Glob Health. 2020;10(1):010502.

39. Powell-Jackson T, Mazumdar S, Mills A. Financial incentives in health: New evidence from India's Janani Suraksha Yojana. J Health Econ. 2015 Sep 1;43:154–69.

40. NGEC. Participation of vulnerable populations in their own programme: the cash transfers in Kenya [Internet]. 2014. Available from: https://www.ngeckenya.org/Downloads/cash-transfer-programme-vulnerable-groups-kenya.pdf

For peer review only

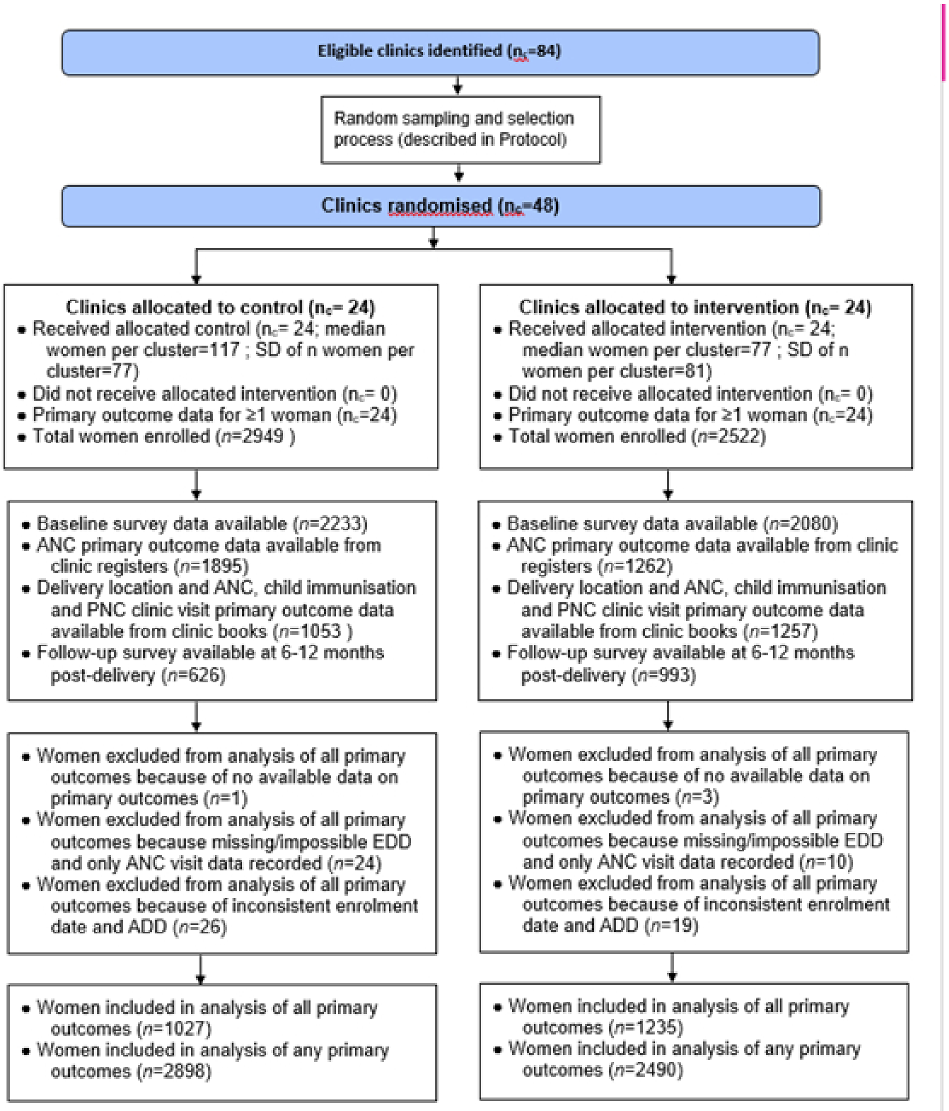

Flow diagram of enrolment and inclusion in analyses by clinic randomization status. n values refer to women and nc to clinics. EDD expected date of delivery, ADD actual date of delivery.

155x171mm (96 x 96 DPI)

Supplementary material

Appendix S1 – Differences from published protocol in data collection and analysis

This summary of differences between the trial protocol and the data collection and analysis that were conducted is closely based on that in the statistical analysis plan (SAP) for the study, which was written prior to unblinding and analysis of data by the trial statistician.

The published Protocol for the study stated that the primary outcomes for the study would be derived by combining data from manual extraction of clinic health records and from the electronic card reading system used to process the trial payments. However, due to technical issues in implementation of the card reading system, it was not possible to extract outcome data from this source. As such, outcome data were also obtained from the personal clinic books of women participating in the trial. Specifically, participants were invited to attend the clinic shortly after the end of the trial with their clinic book for collection of the primary outcomes. For women who however did not attend the clinic for that purpose, data were extracted from the clinic register. Only very limited data on any treatment referrals beyond routine visits were collected and as such this primary outcome (5 of 5 in the Protocol) was dropped from the planned analysis. In addition, there were only data available for the primary outcomes of delivery at a healthcare facility and child immunisation and maternal PNC visits for those women with clinic book available for data extraction (only antenatal visits were extracted from clinic registers).

It was planned that data for the secondary outcomes would be derived from clinic records, the electronic card reading system and a series of four follow-up telephone interviews (after enrolment in all women; 2 weeks after delivery in all women who do not give birth in a facility; 6 months after EDD in 50% of women; 12 months after delivery in the same 50% of women). However, as well as issues with the card reader system, the interviews at 6 and 12 months after delivery were combined into one survey which was primarily delivered towards 12 months after delivery, after some 6-month interviews had already been conducted. The following secondary outcomes were dropped as data were not collected: mother's perception of infant health at 6 and 12 months post-delivery, screening and control of infections for mothers and foetus/baby during pregnancy and postnatal periods.

Perinatal and maternal mortality were recorded if a woman (or family member) attended with their clinic book for data extraction, but completeness of the reporting has not been verified. The remaining outcomes were collected as part of the 6–12-month survey but the survey completion rate was ultimately relatively low, and as such, we have reported available data for the specified secondary outcomes at 6-12 months but have not carried out any formal statistical analyses. The completion rate of the baseline survey was high, but not sufficiently high to use these data to adjust our analyses for maternal characteristics such as socioeconomic factors.

Additional secondary outcomes of the counts of ANC, child immunisation and PNC clinic visits (both with and without applying any eligibility criteria regarding the timing of visits or maximum for each type) per woman were analysed and reported; this was not listed in the original Protocol, where the focus was on the primary outcomes of the *proportion* of each appointment type attended. Analysing the total counts in each case also allows for capture of the potential impact of earlier commencement of ANC care, and creates model outputs useful for health economic analysis. A further additional secondary outcome of GA at enrolment to the trial was also added, to evaluate whether the trial intervention encouraged earlier engagement with antenatal care (whereas the primary outcomes only relate to events following enrolment of each woman). This outcome was available for a large majority of women enrolled in the study.

For peer review only - http://bmjopen.bmj.com/site/about/guidelines.xhtml

The published Protocol stated that the analysis of primary outcomes would use logistic regression for binary outcomes and ordinal regression for ordinal outcomes, with a single pooled effect estimate for the intervention across these outcomes estimated using independence estimating equations. However, since delivery and postnatal outcome data were missing in a substantial proportion of participants, we planned to allow for dependence (i.e., correlation) between each of the outcome variables for each woman using structured random effects models. To facilitate this, visit counts for each woman were analysed as repeated binary observations rather than as ordinal variables.

Appendix S2 – Summary of available mortality data

These data have been obtained from both free-text notes in the study visit records and from information collected in the telephone surveys. It is therefore difficult to gauge the level of ascertainment of these adverse outcomes and the level of completeness will also depend on engagement with care and follow-up, which differed between the control and intervention groups.

Among the 2,949 women enrolled into the control arm, there was one record of intrauterine death of the foetus, one record of a stillbirth, 17 records of neonatal deaths (immediately or up to 1 week following delivery), 33 records of infant deaths up to 18 months after delivery and no records of maternal deaths.

Among the 2,522 women enrolled into the intervention arm, there were two records of intrauterine deaths of the foetus, three records of stillbirth, 23 records of neonatal deaths (immediately or up to 1 week following delivery), 45 records of infant deaths up to 18 months after delivery and two records of maternal deaths. The maternal deaths were both recorded at data extraction from maternal clinic books and appear to have occurred within a year of delivery, but not in the immediate neonatal period.

For peer review only

Additional Tables

Table S1: Cluster-level summaries of the enrolled women included in the primary analysis

Variable	Control clinic	Intervention clinic	ICC
N women per cluster in analysis	113 (67-174;21-301)	77 (31-158, 17-313)	—
**Baseline characteristics**			
Median age	26 (25-27, 22-29)	26 (25-27, 23-30)	—
Proportion nulliparous	0.22 (0.16-0.28, 0.004-0.37)	0.19 (0.10-0.26, 0-0.38)	—
Median GA at enrolment (days)	158 (151-166, 136-179)	153 (149-159, 139-183)	—
Proportion high-risk pregnancies	0.20 (0.11-0.24, 0.06-1)	0.17 (0.14-0.38, 0.06-0.94)	—
**Primary outcomes**			
Mean proportion attendance at eligible ANC clinic appointments (following scheduled visits)	0.61 (0.54-0.67, 0.48-0.75)	0.70 (0.65-0.75, 0.50-0.82)	0.028*
Proportion delivery at health facility	0.92 (0.89-0.96, 0.83-1)	0.90 (0.86-0.94,0.71-1)	0.012†
Proportion attendance eligible PNC clinic appointment (at least one 4-12mo)	0.85 (0.76-0.90, 0.49-1)	0.85 (0.77-0.90, 0.47-0.94)	0.087†
Mean proportion attendance at child immunisation appointments (capped at 4)	0.85 (0.79-0.90, 0.71-1)	0.89 (0.86-0.91, 0.76-0.98)	0.011*

Data shown as median (interquartile range, range) by cluster.
ICC values calculated as $(var(b_i))/(var(b_i)+var(e_{ij}))$ from a linear mixed model with adjustment for intervention and subcounty, where $var(b_i)$ is cluster-level random intercept variance and $var(e_{ij})$ is the residual variance. *Linear mixed model fitted to overall proportion of visits attended as outcome for each woman. †Linear mixed model fitted to binary outcome data (i.e., 0 for no visit and 1 for attendance).

For peer review only

For peer review only - http://bmjopen.bmj.com/site/about/guidelines.xhtml

Table S2: Effect of conditional cash transfers on primary outcome measures for the sensitivity analysis only including the 12 intervention clinics and the 12 control clinics with the highest proportion of enrolled women with payment within 31 days of first ANC clinic visit.

	**Control clinic**	**Intervention clinic**		
**Primary outcome measures**	***n/N*** **(%)**	***n/N*** **(%)**	**OR (95% CI)**	**aOR (95% CI);** ***P***
Attendance at eligible ANC clinic appointments (following scheduled visits)*	3491/5335 (65)	3720/5775 (64)	0.78 (0.55-1.11)	0.80 (0.55-1.17); *P*=0.25
Delivery at health facility†	575/624 (92)	754/838 (90)	0.31 (0.11-0.88)	0.32 (0.11-0.92); *P*=0.04
Attendance at eligible PNC clinic appointment (at least one 4-12mo)‡	503/624 (81)	680/835 (81)	0.88 (0.45-1.74)	0.92 (0.47-1.82); *P*=0.81
Attendance at child immunisation appointments (capped at 4)†	2109/2496 (84)	2960/3352 (88)	1.65 (0.89-3.05)	1.72 (0.93-3.18); *P*=0.08
Pooled intervention estimate	—	—	0.89 (0.65-1.22)	0.92 (0.66-1.27); *P*=0.60

aOR, adjusted odds ratio; OR, odds ratio. *Available for all women included in primary analysis except three (with data from clinic book but no available EDD or ADD). †Available for 1462/3279 women with data obtained from clinic books. ‡Available for 1459 women with data obtained from clinic books and with available ADD or EDD. Available data for attendances and eligible appointments are summed over all women for the *n*/N values.

For peer review only - http://bmjopen.bmj.com/site/about/guidelines.xhtml

Figure S1 Success or failure of prompt payment in individual women (● at 1 or 0), the overall success proportion (blue line) and a 31-day moving average (red line) divided by control and intervention clinics.

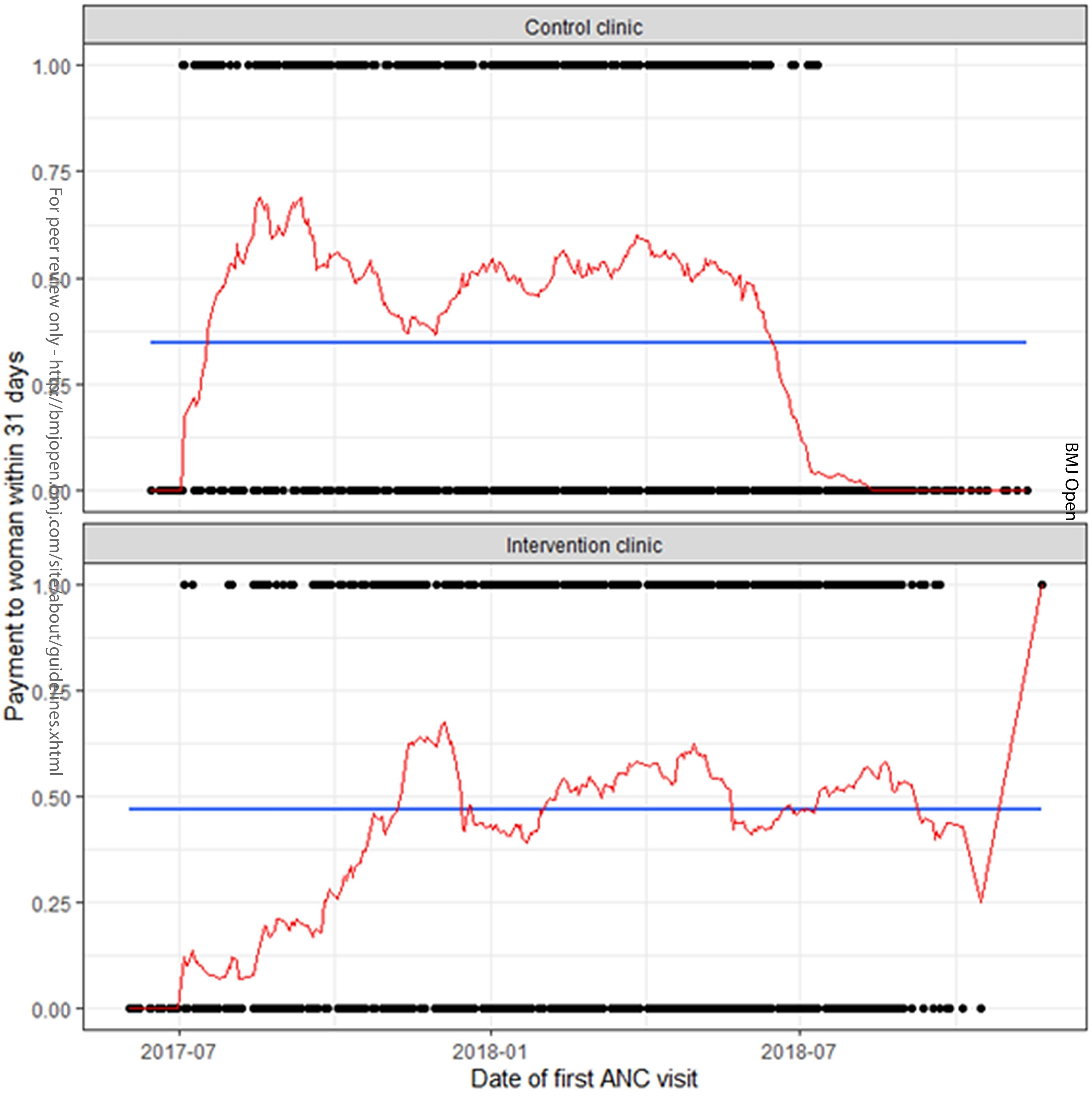

For peer review only - http://bmjopen.bmj.com/site/about/guidelines.xhtml

Figure S2 Success or failure of prompt payment in individual women (● at 1 or 0), the overall success proportion (blue line) and a 31-day moving average (red line) in individual control clinics.

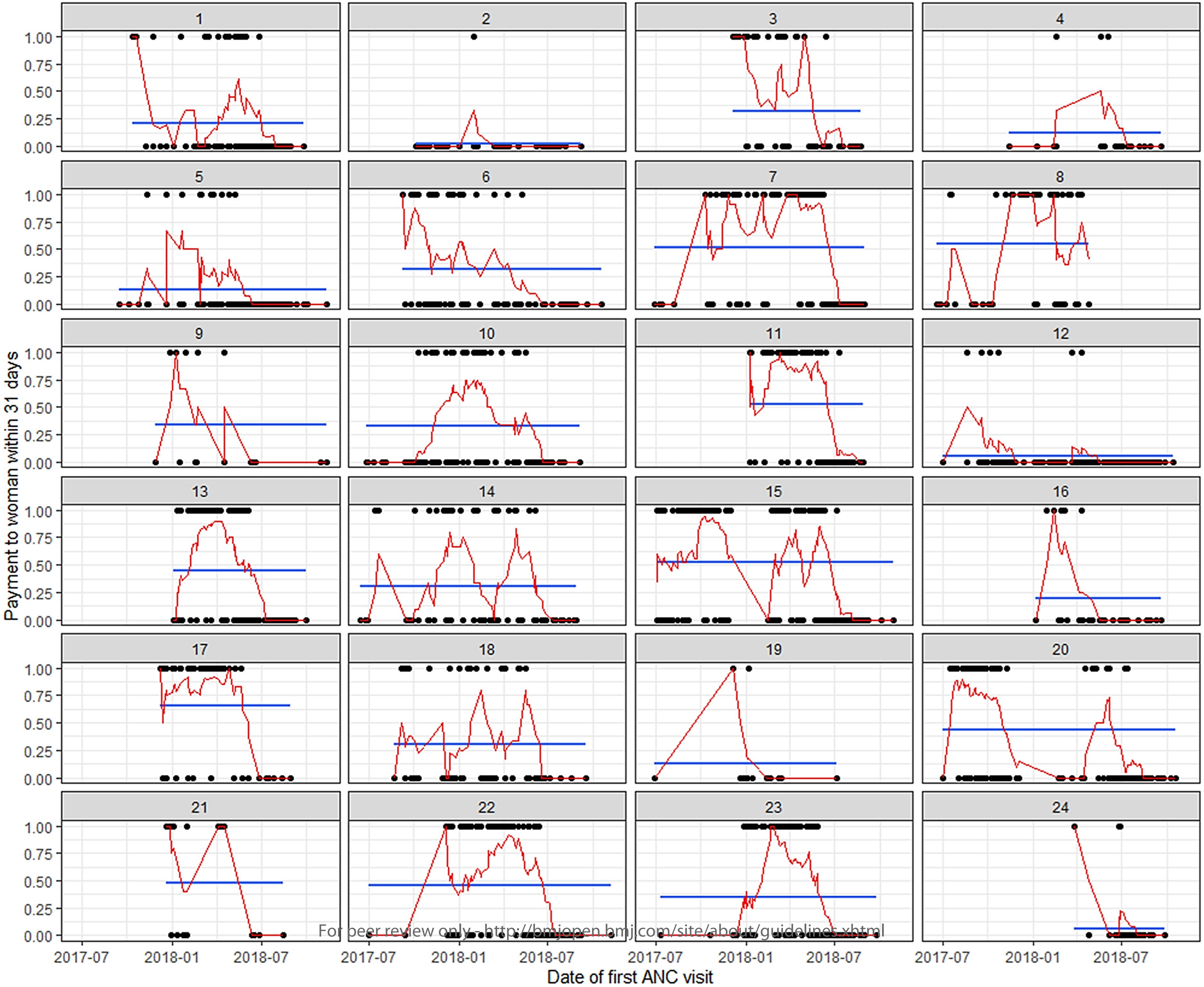

Figure S3 Success or failure of prompt payment in individual women (● at 1 or 0), the overall success proportion (blue line) and a 31-day moving average (red line) in individual intervention clinics.

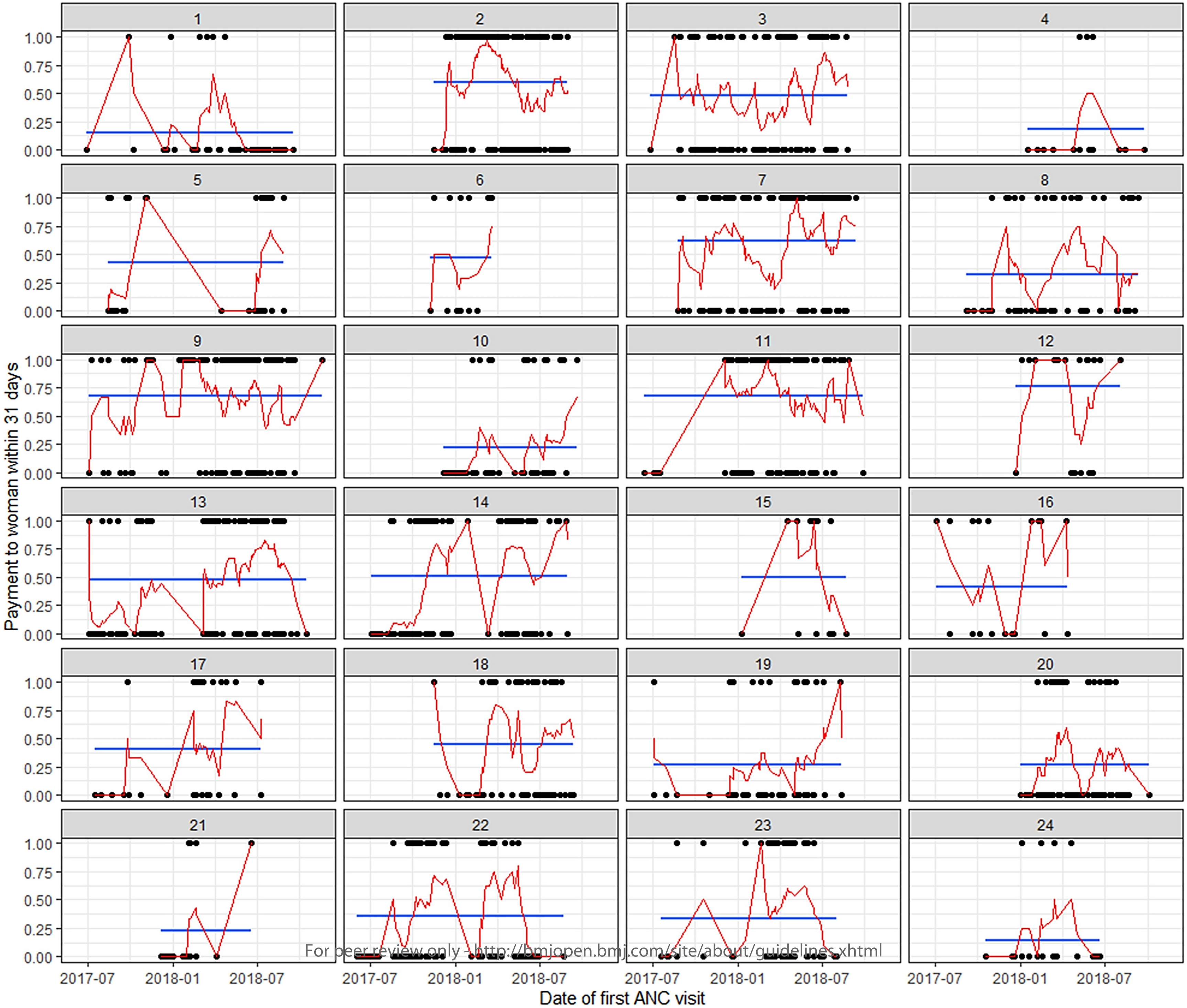

For peer review only - http://bmjopen.bmj.com/site/about/guidelines.xhtml

Figure S4 Exploratory analysis of the clinic-level relationship between proportion of women paid within 31 days of their first ANC clinic visit and the overall attendance proportion for eligible ANC visits. The graph is divided according to control and intervention clinics, with separate linear regression line (blue, with shaded 95%CI) and median percentage women with prompt payment (vertical black line) for each group.

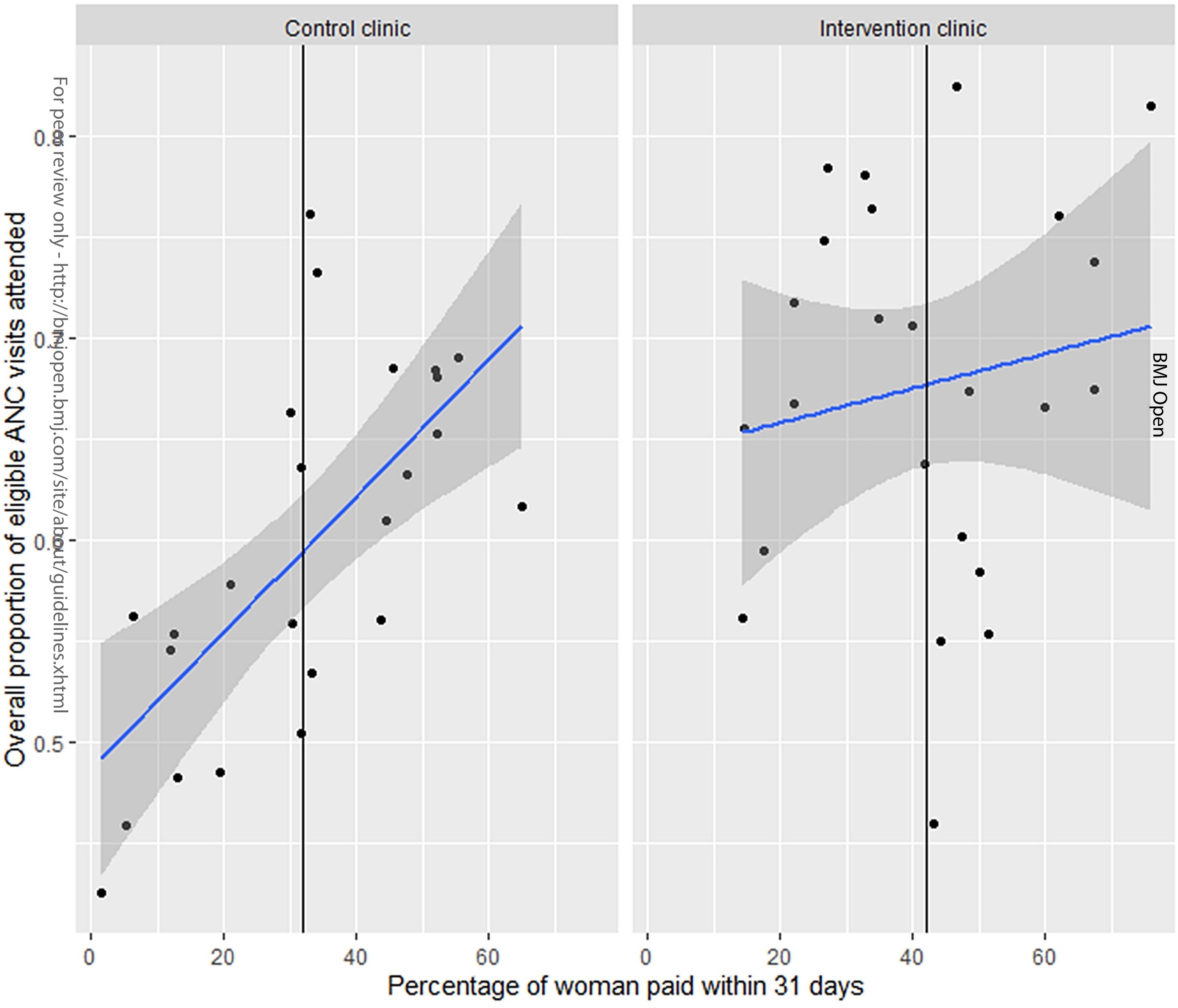

For peer review only - http://bmjopen.bmj.com/site/about/guidelines.xhtml

CONSORT 2010 checklist of information to include when reporting a randomised trial*

Section/Topic	Item No	Checklist item	Reported on page No
**Title and abstract**			
	1a	Identification as a randomised trial in the title	2
	1b	Structured summary of trial design, methods, results, and conclusions (for specific guidance see CONSORT for abstracts)	2
**Introduction**			
Background and objectives	2a	Scientific background and explanation of rationale	4
	2b	Specific objectives or hypotheses	4
**Methods**			
Trial design	3a	Description of trial design (such as parallel, factorial) including allocation ratio	5
	3b	Important changes to methods after trial commencement (such as eligibility criteria), with reasons	Supplementary material p.1
Participants	4a	Eligibility criteria for participants	5
	4b	Settings and locations where the data were collected	5
Interventions	5	The interventions for each group with sufficient details to allow replication, including how and when they were actually administered	5
Outcomes	6a	Completely defined pre-specified primary and secondary outcome measures, including how and when they were assessed	5-6
	6b	Any changes to trial outcomes after the trial commenced, with reasons	6
Sample size	7a	How sample size was determined	6-7
	7b	When applicable, explanation of any interim analyses and stopping guidelines	8
Randomisation:			
Sequence generation	8a	Method used to generate the random allocation sequence	5
	8b	Type of randomisation; details of any restriction (such as blocking and block size)	5
Allocation concealment mechanism	9	Mechanism used to implement the random allocation sequence (such as sequentially numbered containers), describing any steps taken to conceal the sequence until interventions were assigned	5
Implementation	10	Who generated the random allocation sequence, who enrolled participants, and who assigned participants to interventions	5

For peer review only

Blinding	11a	If done, who was blinded after assignment to interventions (for example, participants, care providers, those assessing outcomes) and how	
	11b	If relevant, description of the similarity of interventions	
Statistical methods	12a	Statistical methods used to compare groups for primary and secondary outcomes	7-8
	12b	Methods for additional analyses, such as subgroup analyses and adjusted analyses	7-8; 13-14
**Results**			
Participant flow (a diagram is strongly recommended)	13a	For each group, the numbers of participants who were randomly assigned, received intended treatment, and were analysed for the primary outcome	8-9
	13b	For each group, losses and exclusions after randomisation, together with reasons	8-9
Recruitment	14a	Dates defining the periods of recruitment and follow-up	8
	14b	Why the trial ended or was stopped	8
Baseline data	15	A table showing baseline demographic and clinical characteristics for each group	10-11
Numbers analysed	16	For each group, number of participants (denominator) included in each analysis and whether the analysis was by original assigned groups	9
Outcomes and estimation	17a	For each primary and secondary outcome, results for each group, and the estimated effect size and its precision (such as 95% confidence interval)	11-12
	17b	For binary outcomes, presentation of both absolute and relative effect sizes is recommended	11-12
Ancillary analyses	18	Results of any other analyses performed, including subgroup analyses and adjusted analyses, distinguishing pre-specified from exploratory	13-14
Harms	19	All important harms or unintended effects in each group (for specific guidance see CONSORT for harms)	13-14
**Discussion**			
Limitations	20	Trial limitations, addressing sources of potential bias, imprecision, and, if relevant, multiplicity of analyses	14-15
Generalisability	21	Generalisability (external validity, applicability) of the trial findings	14-15
Interpretation	22	Interpretation consistent with results, balancing benefits and harms, and considering other relevant evidence	14-15
**Other information**			5
Registration	23	Registration number and name of trial registry	
Protocol	24	Where the full trial protocol can be accessed, if available	4
Funding	25	Sources of funding and other support (such as supply of drugs), role of funders	15

*We strongly recommend reading this statement in conjunction with the CONSORT 2010 Explanation and Elaboration for important clarifications on all the items. If relevant, we also recommend reading CONSORT extensions for cluster randomised trials, non-inferiority and equivalence trials, non-pharmacological treatments, herbal interventions, and pragmatic trials. Additional extensions are forthcoming: for those and for up to date references relevant to this checklist, see www.consort-statement.org.

For peer review only - http://bmjopen.bmj.com/site/about/guidelines.xhtml

BMJ Open

Effectiveness of conditional cash transfers (Afya credits incentive) to retain women in the continuum of care during pregnancy, birth and the postnatal period in Kenya: a cluster randomised trial

Journal:	*BMJ Open*
Manuscript ID	bmjopen-2021-055921.R2
Article Type:	Original research
Date Submitted by the Author:	07-Dec-2021
Complete List of Authors:	Vanhuyse, Fedra; Stockholm Environment Institute,<br>Stirrup , Oliver ; University College London, Institute for Global Health<br>Odhiambo, Aloyce; Safe Water and AIDS Project<br>Palmer, Tom; University College London, Institute for Global Health<br>Dickin, Sarah; Stockholm Environment Institute<br>Skordis-Worrall, Jolene; UCL, Institute for Global Health<br>Batura, Neha; University College London, Institute for Global Health<br>Haghparast-Bidgoli, Hassan; University College London, Institute for Global Health<br>Mwaki, Alex; Safe Water and Aids Project (SWAP)<br>Copas, Andrew; University College London, Institute for Global Health
**Primary Subject Heading**:	Global health
Secondary Subject Heading:	Health services research
Keywords:	PUBLIC HEALTH, Community child health < PAEDIATRICS, Maternal medicine < OBSTETRICS

SCHOLARONE™ Manuscripts

I, the Submitting Author has the right to grant and does grant on behalf of all authors of the Work (as defined in the below author licence), an exclusive licence and/or a non-exclusive licence for contributions from authors who are: i) UK Crown employees; ii) where BMJ has agreed a CC-BY licence shall apply, and/or iii) in accordance with the terms applicable for US Federal Government officers or employees acting as part of their official duties; on a worldwide, perpetual, irrevocable, royalty-free basis to BMJ Publishing Group Ltd ("BMJ") its licensees and where the relevant Journal is co-owned by BMJ to the co-owners of the Journal, to publish the Work in this journal and any other BMJ products and to exploit all rights, as set out in our licence.

The Submitting Author accepts and understands that any supply made under these terms is made by BMJ to the Submitting Author unless you are acting as an employee on behalf of your employer or a postgraduate student of an affiliated institution which is paying any applicable article publishing charge ("APC") for Open Access articles. Where the Submitting Author wishes to make the Work available on an Open Access basis (and intends to pay the relevant APC), the terms of reuse of such Open Access shall be governed by a Creative Commons licence – details of these licences and which Creative Commons licence will apply to this Work are set out in our licence referred to above.

Other than as permitted in any relevant BMJ Author's Self Archiving Policies, I confirm this Work has not been accepted for publication elsewhere, is not being considered for publication elsewhere and does not duplicate material already published. I confirm all authors consent to publication of this Work and authorise the granting of this licence.

For peer review only

Title: Effectiveness of conditional cash transfers (Afya credits incentive) to retain women in the continuum of care during pregnancy, birth and the postnatal period in Kenya: a cluster randomised trial

Corresponding author: Fedra Vanhuyse, Stockholm Environment Institute, Linnégatan 87D, Box 24218, 104 51 Stockholm, Sweden.

Email: fedra.vanhuyse@sei.org

Authors: Fedra Vanhuyse*[1], Oliver Stirrup[2], Aloyce Odhiambo[3], Tom Palmer[2], Sarah Dickin[1], Jolene Skordis[2], Neha Batura[2], Hassan Haghparast-Bidgoli[2], Alex Mwaki[3] and Andrew Copas[2]

[1] Stockholm Environment Institute, Linnégatan 87D, Box 24218, 104 51 Stockholm, Sweden.

[2] Institute for Global Health, University College London, London, UK.

[3] Safe Water and AIDS Project (SWAP), P.O. Box 3323, Kisumu 40100, Kenya.

Key words: public health, community child health, maternal medicine

Word count: 4,840

For peer review only

Abstract

Objectives
Given high maternal and child mortality rates, we assessed the impact of Conditional Cash Transfers (CCTs) to retain women in the continuum of care (antenatal care (ANC), delivery at facility, postnatal care (PNC) and child immunization).
Design
We conducted an unblinded 1:1 cluster-randomized controlled trial.
Setting
48 health facilities in Siaya County, Kenya were randomized. The trial ran from May 2017 to December 2019.
Participants
2922 women were recruited to the control and 2522 to the intervention arm.
Interventions
An electronic system recorded attendance and triggered payments to the participant's mobile for the intervention arm (4.5 USD), and phone credit for the control arm (0.5 USD). Eligibility criteria were resident in the catchment area and access to a mobile phone.
Primary outcomes
Primary outcomes were any ANC, delivery, any PNC between 4- 12 months after delivery, childhood immunization, and referral attendance to other facilities for ANC or PNC. Given problems with the electronic system, primary outcomes were obtained from maternal clinic books if participants brought them to data extraction meetings (1257 (50%) of intervention and 1053 (36%) control arm participants). Attendance at referrals to other facilities is not reported because of limited data.
Results
We found a significantly higher proportion of appointments attended for ANC (67% vs. 60%, adjusted OR (aOR) 1.90; 95% CI 1.36-2.66) and child immunization (88 vs 85%; aOR 1.74; 95% CI 1.10-2.77) in intervention than control arm. No intervention effect was seen considering delivery at the facility (90 vs 92%; aOR 0.58; 95% CI 0.25-1.33) and any PNC attendance (82 vs 81%; aOR 1.25; 95% CI 0.74-2.10) separately. The pooled odds ratio across all attendance types was 1.64 (1.28-2.10).
Conclusions
Demand-side financing incentives, such as CCTs, can improve attendance for appointments. However, attention needs to be paid to the technology, the barriers that remain for delivery at facility and PNC visits and encouraging women to attend ANC visits within the recommended WHO timeframe.
Trial registration
NCT03021070; clinicalTrials.gov

Strengths and limitations of this study

- Technical issues with the electronic system and at times low participation of health workers resulted in many visits not being registered, and only 26% of the payments triggered automatically.
- Manual payments needed to be triggered, which resulted in delays.
- This delay in payment could have diluted the impact of the intervention and prevented service utilization in the latter stages of pregnancy or after babies were born.
- As manual data abstraction from clinic registers and from the women's maternal clinic books was necessary, we obtained near complete data for ANC attendance but limited data on facility delivery, PNC and child immunization and no data on referral attendance.
- The potential bias is however limited to a degree by our approach to analysis in which all outcomes are modelled simultaneously.

For peer review only

Introduction

Every year an estimated 295,000 maternal deaths occur globally, with 99% occurring in low and middle-income countries, and almost two thirds in Africa[1]. In Sub-Sahara Africa, the maternal mortality rate was 542 deaths per 100,000 live births in 2017[1]. In Kenya, the maternal mortality rate in 2014 was 362 deaths per 100,000 live births[2], categorized as "high" according to the World Health Organization (WHO). Globally, an estimated 2.4 million infants die in the first month of life (40% of all deaths) and 1.6 million at age 1–11 months (25%)[3]. Sub-Saharan Africa has the highest neonatal mortality rate at a median of 27 deaths per 1,000 live births and the highest under-five mortality rate, with a median of 76 deaths per 1,000 live births[3]. In Kenya, the median infant mortality rate was 21 deaths per 1,000 live births and the median under-five mortality rate was 43 deaths per 1,000 live births[2], which are amongst the highest in the region.

Most maternal and neonatal deaths are avertible through the use of healthcare interventions that prevent or manage pregnancy-related complications such as postpartum haemorrhage and infectious diseases[4,5]. The importance of health service utilisation for maternal and child health outcomes has been extensively documented, including for antenatal care (ANC)[6–8], facility delivery[9, 10], postnatal care (PNC)[11] ,and across the continuum of care[12]. In Kenya, while 96% of women receive some form of ANC, less than three in five receive the four ANC visits that the WHO recommends[2]. Only one in five have their first ANC visit during the first trimester as recommended by the WHO[2], which would allow to monitor the health of both mother and child more effectively. Almost four in ten babies are delivered at home, and 62% of new-borns do not receive a PNC check-up in the first week after birth[2]. Almost half (47%) of mothers do not receive a PNC check-up in the first two days after birth[2]. Lower attendance at healthcare facilities could be due to a lack of skilled health workers, poverty, distance, lack of information, inadequate services and cultural practices[13]. Other research[14] reported costs charged for ANC visits, nurses' behaviour, and the timing of the visits as the main barriers for attending ANC visits. In Kenya, a survey showed that financial barriers (costs of care for other children, food, new clothes), and lack of transport and distance to health care facilities were the main barriers[15].

Aside from programmes to improve the quality and reach of the service (supply side interventions), demand side financing interventions have been set up to incentivise women to attend visits. Examples of such interventions include mobile phone text message reminders for ANC visits in Zanzibar[16] and for PNC visits in Tanzania[17], as well as the use of conditional cash transfers (CCTs). In Kenya, a conditional cash transfer intervention in Vihiga County was found to increase facility delivery by 7.9 percentage points[18]. A recent study in Nigeria[19] found that payments for retention from ANC to PNC resulted in more women attending the visits (26% of women in the intervention arm compared to 12% in the control arm), leading to a 22% reduction in the stillbirth rate. Recent systematic reviews on the demand-side interventions for maternal care[20] and on CCTs[21–24] found increased utilisation of services, but not always better outcomes.

The Afya trial aims to test the effectiveness of a conditional cash transfer to retain women in the continuum of care, from their first ANC visit until their children reach 1 year of age in Siaya County, Kenya. The CCT aimed at tackling multiple barriers to care, as described in the trial's protocol[25]: women would receive equal-sized cash transfers following a visit (ANC, delivery at facility, PNC visit and childhood immunization), as well as a reminder for their visit by text message and medical staff would be trained in the technology and incentivised for each woman they enrolled in the trial. Unlike a study in Nigeria[19], in Afya, the CCT was done through a card reader system rather than cash and, additionally, it initiated transfers for each individual visit, rather than being conditional on receiving an entire package of care. In this paper, we present the results of the impact of the Afya trial.

For peer review only - http://bmjopen.bmj.com/site/about/guidelines.xhtml

Methods

Study setting, design and randomisation

We conducted a cluster randomized controlled trial, with equal allocation to intervention and control arms, in Siaya county, Kenya. The units of randomisation were Level 2 or 3 health facilities (Dispensaries and Health Centres, respectively). The randomisation of facilities was stratified by the six sub-Counties and ensured equal allocation to study arms within each stratum without any overlap of catchment areas, as described in detail in the trial protocol[25]. In summary, at a public forum with the county government early 2016, the implementing partner wrote the names of 60 shortlisted facilities on pieces of paper and folded them to hide the names, then included them in transparent boxes, one for each sub-county. Each subcounty had an (even) number of facilities to recruit to the trial proportional to subcounty size. The health management teams from each subcounty selected the pieces of paper, one by one. The first was allocated to intervention, second to control. For each selected facility, county officials from the selected subcounty mapped the location and catchment area of the facility on a large map of the county. If a subsequently selected facility had an overlapping catchment area with a previously selected facility, the newly selected facility was rejected, and another drawn to take its place. This process continued until 48 facilities were selected and allocated for the trial.

Health facility staff determined whether a pregnant woman met the study eligibility criteria by administering screening questions at the end of her first ANC visit, with the screening questions provided in the trial protocol [25]. All women meeting the criteria were eligible for recruitment during the study recruitment period. Criteria for enrolment were women attending their first ANC visit; long-term resident of the catchment area served by the health facility (living in the area for at least 6 months); access to a mobile phone that belongs either to themselves or to a member of their household or person whom they trust. The criterium on residence provided additional assurance that women went to the facility within their catchment area, thereby reducing contamination with other facilities. Oral informed consent was asked in the local language, and then written down on the participant's enrolment form. Refusals were recorded.

Intervention

The intervention was a CCT payment for each facility appointment attended for ANC, delivery, postnatal care, and childhood immunization. Detailed definitions can be found in Appendix S1. For each scheduled health visit made following enrolment, women in the intervention arm received a cash transfer of KSH 450 (4.5 USD) on their mobile phones. Women at the control clinics were granted KSH 50 (0.5 USD) mobile phone airtime for each scheduled visit to encourage them to bring their clinic booklet to appointments. In both trial arms, women were issued with a trial card at recruitment and at all facilities there was a card reader, which provided the connection between the trial card and an online portal which stored participants' data on visits and payments. Payments to the women were triggered by tapping the card on a card reader, which also logged the visit in an online portal[26]. In the event of problems with the card reader, or if the woman did not bring her card to the appointment, payments could alternatively be processed manually by contacting the implementing partner: once the visit was verified with the facility, the implementing partner entered the visit data in the portal, which would then trigger a payment as well Nurses were given KSH 400 (4 USD) per woman enrolled during the trial, and an additional KSH 100 (1USD) per woman enrolled at the end of the trial for their collaboration in the trial. These payments were transferred to the nurses electronically. Details of the intervention design are presented in the protocol[25].

For peer review only - http://bmjopen.bmj.com/site/about/guidelines.xhtml

Patient and Public involvement statement

Patients or the public were not involved in the design, conduct, reporting, or dissemination plans of the research. The assessment of the burden of the intervention on patients is reported in[27].

Ethics statement

Ethical approval was granted by Maseno University in Kenya (MSU/DRP/MUERC/00294/16).

Trial outcomes

The primary outcomes were: 1) attendance or missed attendance at each eligible ANC appointment after recruitment; 2) delivery at a health facility; 3) attendance for at least one PNC appointment between 4 and 12 months after delivery; 4) attendance or missed attendance at each expected child immunization appointment; 5) attendance at referrals to other facilities for ANC, PNC or child immunization. We define 'eligible visits' for the purpose of statistical analysis of the impact of the intervention within a clearly defined framework, but all scheduled pre- and post-natal clinic visits should have triggered a payment. The PNC attendance outcome described in the protocol was the 'proportion of required postnatal visits honoured after recruitment into the study'. However, we have used a simplified outcome here because the required appointment schedule was not recorded for each patient. We also restricted attendances to the period 4-12 months after delivery because on blinded review of the available data, prior to writing the statistical analysis plan (SAP), very few visits prior to 4 months post-delivery were coded as PNC. The vast majority of visits prior to 4 months were recorded as vaccination appointments. though it is likely some women also received PNC. Vaccinations over the 12 months after delivery were recorded. The details of vaccinations given were not collected, and so the vaccination outcome is based purely on the number of recorded visits from an expected number of four.

The following secondary outcomes are also reported and analysed according to trial arm: 1) attendance of all eligible maternal, new-born and child health (MNCH) care visits, both prenatal and postnatal, for each woman; 2) the count of attended ANC and child immunisation clinic visits eligible for the primary outcome variables for each woman; 3) the total number of ANC, child immunisation and PNC clinic visits (without applying any eligibility criteria) for each woman; 4) gestational age (GA) at first ANC visit (and enrolment to study). The secondary outcomes of clinic visit counts and GA at first ANC visit were not listed in the trial protocol but were prespecified in the SAP to aid interpretation of the study results.

The primary outcome of attendance at referrals to other facilities for ANC, PNC or child immunization is not reported because of very limited data. The following planned secondary outcomes are reported without formal statistical analysis because of low levels of data completeness: maternal and neonatal mortality, self-rated wellness, exclusive breastfeeding, and contraceptive use. The following secondary outcomes were dropped because of lack of available data: timeliness of health visits (recorded visits could not consistently be matched up to scheduled dates), and infection screening. These changes were specified in the SAP. Appendix S1 contains further details of changes made to the data collection and analysis compared to the protocol, arising from data collection challenges.

Data collection

Data were collected throughout the trial from an electronic card reading system, and baseline and follow-up surveys. The electronic card reading system captured the enrolled women's phone number, expected delivery date (EDD), parity, the clinic she enrolled at, the visits she attended and the payments she received. The baseline survey, carried out by telephone following enrolment, collected socio-demographic data. We initially planned to conduct follow-up interviews with 50% of enrolled

For peer review only - http://bmjopen.bmj.com/site/about/guidelines.xhtml

women at 6 and 12 months post-delivery to collect secondary outcomes. However, due to limited resources and lower than anticipated response rates after some 6-month interviews had been conducted, we adopted a pragmatic approach and conducted one follow-up survey at around 12 months after delivery.

Problems with the implementation of the technical system and periods of disengagement from clinic staff resulted in a large proportion of visits not being registered on the system. Therefore, data on women's visits were manually extracted from clinic health records after the trial was completed. Trial staff visited each facility, after arranging for trial participants to be invited to attend the facility with their maternal clinic book. Data on the primary outcomes of ANC, child immunisation and PNC visits and delivery at a healthcare facility were extracted from maternal clinic books. The books very rarely contained records of any referral visits. For women who did not attend the data extraction, data on the primary outcome of ANC visits were extracted from health facility registers, but data on other visits were not available from this source. Any missing payments were transferred to the participating women following the manual data extraction at the end of the trial.

Data on payments made to trial participants, whether triggered by the card reader or manually, were extracted from the trial portal. These data are reported in the process evaluation paper, along with details of the challenges with the electronic system[27].

Sample size

We analysed all primary outcomes jointly, to maximise power, aid interpretation and minimise testing (see Statistical Analysis). However, for our sample size calculation, we considered the power to detect an effect of the intervention on one primary outcome, which we also assumed to be a binary indicator that all attendances were made, as this is simple and conservative in the power achieved. The expected prevalence of these indicators in the control arm ranged between 30 and 80%. In the absence of specific information on the likely intra cluster correlation (ICC) we considered a range between 0.005 (low) and 0.025 (moderate). Our planned sample size was 48 clusters covering the catchment areas of selected level 2 and 3 health facilities (24 per arm) and an average cluster size of 150 participants. At a low ICC, the design effect (DE) would be 1.745 and hence the effective sample size (ESS) would be 2,063 participants per study arm. At a moderate ICC, the DE would be 4.725 and hence the ESS 762 per arm. Power to detect absolute differences is lowest when the prevalence is 50% and highest when the prevalence is either high (towards 100%) or low (towards 0%). Here we considered the prevalence in the control arm to range between 50% ('worst-case scenario') and 80% ('best-case scenario'). We considered the standard 5% significance level. If the prevalence of the outcome is 50% in the control arm, the sample size provides 80% power to detect an improvement to 54.5% in the intervention arm if the ICC is low and 57.5% if the ICC is moderate. If the prevalence of the outcome is 80% in the control arm, the sample size provides 80% power to detect an improvement to 83.5% if the ICC is low and 85.5% if the ICC is moderate.

Data definitions and processing

For the purpose of statistical analysis, eligible ANC visits were defined based on the recorded 'next scheduled visit date' and noting that this was typically 4 weeks later each time. For each recorded visit starting with enrolment, we evaluated whether the next observed ANC visit was within ±2 weeks of the 'next scheduled visit date': recording a successful ANC visit if yes, but a missed visit if not. However, we did not count the visit as either successful or missed if the next observed visit was more than 2 weeks early compared to the next scheduled visit or delivery occurred within 2 weeks of a 'missed' scheduled visit. Whether that next visit is early, on time, or late, we assessed subsequent visit attendances in the same way. We created hypothetical scheduled visits every 4 weeks for any gaps in

For peer review only - http://bmjopen.bmj.com/site/about/guidelines.xhtml

observations, judged according to the same criteria. The 'successful' and 'missed' appointments were then summed over all scheduled and hypothetical appointments for each woman.

For the primary outcomes of PNC visits, we defined a binary indicator of one or more PNC visit 4-12 months postpartum. This was used to provide a simple indicator of engagement with postnatal care for each woman, given that the appropriate number of PNC visits may differ between women. We defined child immunisation visits as the total number of visits recorded post-partum (excluding vaccination at delivery, but without other time restrictions) truncated at a maximum of four, since that is the typical number required for full immunisation.

Retention in the full continuum of antenatal, perinatal, and postnatal care (a secondary outcome) was defined as a binary indicator of attendance of all eligible ANC, child immunisation and PNC visits and delivery at a healthcare facility for each woman and is available for those women who brought their clinic book for data extraction.

Statistical analysis

The primary outcomes of attendance of eligible ANC and child immunisation appointments comprise repeat binary observations for each individual woman, whilst the primary outcomes of delivery at a health facility and PNC attendance between 4–12 months are each single binary variables for each woman. A summary odds ratio is presented as the main effect measure for the trial, estimated from a model that assumes the odds ratio is the same across the four primary outcomes. We also report separate effect estimates for each primary outcome. A mixed effects logistic regression model was used to jointly analyse the observed primary outcomes. This approach assumes that, given their ANC attendances (recorded for nearly all women), whether a woman did or didn't bring their clinic book for extraction of the other outcomes was unrelated to the values of those outcomes. At the level of each woman correlated random effects were specified for (1) attendance of ANC clinic visits and (2) grouped outcomes of delivery at a healthcare facility, attendance of vaccination visits and attendance of at least one PNC clinic visit at 4–12 months post-partum. Clinic-level random effect terms were defined for (1) ANC visits, (2) delivery at a healthcare facility and (3) PNC and child immunisation visits, with unrestricted correlations between these.

The secondary outcomes of counts of ANC clinic, PNC clinic and child immunisation visits were analysed using a multivariate Poisson mixed effects model, with random intercept terms at patient and the clinic levels. Marginal mean differences in counts between intervention groups were estimated. The secondary outcome of GA at enrolment to the trial was analysed using a linear mixed effects model, with random intercept term at clinic level. Retention in the full continuum of care was analysed using a logistic regression mixed effects model, with random intercept term at clinic level. As the completion rate of the follow-up survey was lower than expected, the secondary outcome data obtained is reported in a descriptive summary but not compared between trial arms.

Our main analyses are conducted as randomised (i.e., intention to treat) but a 'per-protocol' style sensitivity analysis of the primary outcomes was also prespecified. As there was not a clear division between clinics that did and did not achieve the intended payment schedule, the sensitivity analysis included the 12 intervention clinics and the 12 control clinics with the highest proportion of enrolled women with payment within 31 days of first ANC clinic visit.

All analyses were adjusted by the baseline maternal parity ('0' vs '≥1'), by the presence of any maternal medical conditions leading to classification of the pregnancy as high-risk (HIV with or without ART, diabetes, hypertension, malaria, each coded with separate indicator variables) and by the clinic-level variable of sub-county. Adjusted effect measures are considered the primary effect measures though unadjusted effect estimates are also reported. The analysis followed a pre-specified statistical analysis

For peer review only - http://bmjopen.bmj.com/site/about/guidelines.xhtml

plan, which was finalised after data collection but prior to any unblinded analysis. Selection of maternal characteristics as adjustment variables was based on their inclusion in the core enrolment dataset, the associated absence of missing data for these items and their potential to predict the outcomes of interest. Sub-county was included as an adjustment variable because of its use as a stratification factor in the randomisation process for the study.

As recommended by the MRC framework for the evaluation of complex interventions[28], a detailed process and economic evaluation will be published in two forthcoming papers[27, 29].

Results

The trial was conducted in 24 intervention and 24 control clinics and enrolled a total of 2522 women at intervention clinics and 2949 at control clinics over a period from May 2017 to December 2019 (Figure 1). Only 11 eligible women declined enrolment at intervention clinics and 58 at control clinics. Based on the background data on ANC attendance in the study region in 2015, it was expected that each of the 48 facilities would recruit 150 participants into the trial, meeting the target sample size of 7,200 eligible women during the trial period. However, enrolment stopped before the target sample size of 7,200 could be reached due to delays arising from the nurses' strike during which enrolment was paused at many clinics (see, e.g.,[30] who discussed the strike and its impacts on health care delivery), and as the trial was intended to run until 2018 initially. The vast majority (5,388 or 98.5%) of women had data on ANC attendances, but data from maternal clinic books on all primary outcomes were available in a minority of women (2,262/5,388, 42.0%). Socio-demographic characteristics for all enrolled women are presented in Table 1, with cluster-level summaries in Table S1. Baseline survey data were available in 4,313/5,471 (78.8%) women, and a summary of selected fields by arm is presented in Table 2. Table 1 and 2 demonstrate very good balance between arms.

Table 1: Baseline and pregnancy characteristics of the enrolled women included in the primary analysis, obtained from enrolment data, clinic book and clinic registry data

**Variable**		**Control clinic, *n* (%) or median (IQR)**	**Intervention clinic, *n* (%) or median (IQR)**
**Baseline characteristics**			
Age	Years (median (IQR) [*n*])	26 (22-31) [2738]	26 (22-31) [2349]
Parity	0	606 (21)	488 (20)
	1	698 (24)	591 (24)
	2	587 (20)	485 (19)
	⩾3	1007 (35)	926 (37)
GA at enrolment	Weeks+days (median (IQR) [*n*])	22+4 (17+4 to 27+2) [2898]	22+3 (17+2 to 27+0) [2487]
HIV status	Negative	2481 (86)	2142 (86)
	Positive, on treatment	398 (14)	322 (13)
	Positive, not on treatment	19 (1)	26 (1)
Diabetes	No	2889 (99.7)	2482(99.7)
	Yes	9 (0.3)	8 (0.3)
Hypertension	No	2892 (99.8)	2485 (99.8)
	Yes	6 (0.2)	5 (0.2)
Malaria	No	2603 (90)	2164 (87)
	Yes	295 (10)	326 (13)
Total high-risk	No	2245 (77)	1850 (74)

For peer review only - http://bmjopen.bmj.com/site/about/guidelines.xhtml

pregnancies	Yes	653 (23)	640 (26)
**Pregnancy Characteristics**			
GA at delivery	Weeks+days (median (IQR) [*n*])	39+3 (37+0 to 41+1) [1004]	39+0 (36+4 to 41+0) [1206]

IQR, interquartile range. GA gestational age

Table 2: Sociodemographic characteristics of the enrolled women from baseline survey

**Variable**		**Control clinic, *n* (%)**	**Intervention clinic, *n* (%)**
Enrolled women	Total *n*	2949	2522
Baseline survey	Available	2233 (76)	2080 (82)
	Missing	716 (24)	442 (18)
Self-rated maternal health	Very good	4 (0.2)	3 (0.1)
	Good	771 (35)	718 (35)
	Moderate	1445 (65)	1351 (65)
	Bad	12 (0.5)	6 (0.3)
	Very Bad	0 (0)	0 (0)
	Missing*	1 (0.04)	2 (0.1)
Maternal education level	None or only literacy	15 (0.7)	17 (0.8)
	Primary incomplete	631 (28)	540 (26)
	Primary complete	860 (39)	796 (38)
	Secondary incomplete	352 (16)	343 (16)
	Secondary complete	292 (13)	292 (14)
	University/college	79 (4)	91 (4)
	Don't know/other/missing*	4 (0.2)	1 (0.05)
Mode of travel to facility for enrolment visit	Public transport, e.g. bus	727 (33)	718 (35)
	Mini bus taxi	0 (0)	0 (0)
	Metered /taxi	4 (0.2)	7 (0.3)
	Walking	1485 (67)	1341 (64)
	Car	1 (0.04)	1 (0.05)
	Other	16 (0.7)	11 (0.5)
	Missing*	0 (0)	2 (0.1)
Travel time to facility for enrolment visit	<1 hour	1444 (65)	1314 (63)
	1-2 hours	741 (33)	699 (34)
	2-3 hours	43 (2)	60 (3)
	>3 hours	4 (0.2)	5 (0.2)
	Missing*	1 (0.04)	2 (0.1)

*Of those women with baseline survey recorded.

Primary and secondary outcomes

The proportion of eligible ANC appointments attended was significantly higher in the intervention arm compared to control (67 vs. 60%; aOR 1.90; 95% CI 1.36-2.66). A smaller increase was also demonstrated in the proportion of eligible immunisation appointments attended (88 vs 85%; aOR 1.74; 95% CI 1.10-2.77). For the other primary outcomes reporting was similar between arms (Table 3). The pooled aOR for the intervention effect giving a summary measure across all primary outcomes was 1.64 (95%CI 1.28-2.10), p<0.001. The intervention effect on the number of eligible ANC attendances, expressed as an adjusted marginal change (Table 3), was an increase of 0.31 (0.15 to 0.47). The adjusted marginal change in eligible immunisation attendances was not significant (0.14, -

For peer review only - http://bmjopen.bmj.com/site/about/guidelines.xhtml

0.12 to 0.41). Increases in attendances were seen for all visit types when the eligibility requirements defined for the primary analysis were removed, thereby considering all healthcare visits (including any unscheduled visits). The intervention had no effect on the timing of first ANC visit, the mean GA at enrolment was 22.2 weeks for intervention and 22.3 weeks for control, or quite a few weeks after the recommended first visit by the WHO[31] but consistent with other studies in low- and middle-income countries[32]. Postnatal surveys at 5-18 months after delivery were completed by a minority of women, selected outcomes are reported by arm in Table 4. Maternal and perinatal mortality were not systematically recorded, but the available data on these outcomes are summarised in Appendix S2. The ICC was 0.028 for the primary outcome of ANC visits, 0.012 for delivery at a health facility, 0.087 for attendance of at least one eligible PNC visit and 0.011 for immunisation visits.

Table 3: Effect of conditional cash transfers on primary and secondary outcome measures

	**Control clinic**	**Intervention clinic**		
**Primary outcome measures**	***n/N* (%)**	***n/N* (%)**	**OR (95% CI)**	**aOR (95% CI); *P***
Attendance at eligible ANC clinic appointments (following scheduled visits)*	5,827/9,736 (60)	5,741/8,595 (67)	1.95 (1.39-2.72)	1.90 (1.36-2.66); *P*<0.001
Delivery at health facility†	945/1,027 (92)	1,115/1,238 (90)	0.59 (0.26-1.35)	0.58 (0.25-1.33); *P*=0.20
Attendance at one or more eligible PNC clinic appointment (4-12 months after delivery)‡	831/1,027 (81)	1,016/1,235 (82)	1.26 (0.75-2.12)	1.25 (0.74-2.10); *P*=0.40
Attendance at child immunisation appointments (capped at 4)†	3,498/4,108 (85)	4,353/4,952 (88)	1.76 (1.10-2.80)	1.74 (1.10-2.77); *P*=0.02
Pooled intervention estimate	—	—	1.69 (1.32-2.17)	1.64 (1.28-2.10); *P*<0.001
**Secondary outcome measures**	**Control clinic**	**Intervention clinic**		
	***n/N* (%)**	***n/N* (%)**		**aOR (95% CI)**
Attendance at all eligible ANC and PNC visits, child immunisation appointments and delivery at a healthcare facility (per woman)	480/1,027 (47)	632/1,235 (51)		1.14 (0.82 to 1.57)
	Mean (95% CI) ¶, Median (IQR)	***Mean (95% CI) ¶, Median (IQR)***		**Average difference in mean (95% CI) ¶**
**Visit counts (eligible for primary outcome)**				
Total attendances at eligible ANC clinic appointments (following scheduled visits)*	2.04 (1.93-2.14) 2 (0-3)	2.34 (2.22-2.47), 2 (1-3)		0.31 (0.15 to 0.47)
Total attendances at eligible child immunisation appointments (capped at 4)†	3.33 (3.14-3.52), 4 (3-4)	3.48 (3.29-3.67), 4 (4-4)		0.14 (-0.12 to 0.41)

For peer review only - http://bmjopen.bmj.com/site/about/guidelines.xhtml

**Visit counts (no eligibility criteria applied)**			
Total attendances at ANC clinic appointments‡	2.05 (1.93-2.18), 2 (0-3)	2.42 (2.27-2.57), 2 (1-4)	0.37 (0.18 to 0.56)
Total attendances at PNC clinic appointments†	4.17 (3.90-4.44), 4 (2-7)	4.76 (4.46-5.06), 5 (2-7)	0.58 (0.19 to 0.98)
Total attendances at child immunisation appointments†	3.64 (3.40-3.88), 4 (3-5)	4.00 (3.75-4.26), 4 (4-5)	0.36 (0.02 to 0.71)
	**Mean (95% CI)**	**Mean (95% CI)**	**aΔ (weeks) (95% CI)**
GA at enrolment (weeks)*	22.3 (21.9-22.7)	22.2 (21.8-22.6)	-0.1 (-0.6 to 0.5)

aΔ, adjusted difference in mean; aOR, adjusted odds ratio; OR, odds ratio.
*Available for all women included in primary analysis except three (with data from clinic book but no available EDD or ADD).
†Available for 2265/5388 women with data obtained from clinic books. ‡Available for all women included in primary analysis.
¶Marginal value derived from multivariate Poisson mixed effects model applied to visit counts, estimated over the baseline characteristics of all 5388 women with data for at least one of the primary outcomes.
‡Available for 2262 women with data obtained from clinic books and with available ADD or EDD. Available data for attendances and eligible appointments are summed over all women for the *n*/N values.

Table 4: Data from follow-up surveys completed 5-18 months after delivery

**Variable**		**Control clinic**	**Intervention clinic**
Enrolled women	Total *n*	2,949	2,522
Survey answers 5-18 months after delivery			
Survey	Available	626 (21)	993 (39)
	Missing	2,323 (79)	1,529 (61)
Time from delivery at survey (days)		379 (273-463)	375 (273-469)
Self-rated maternal health	Very good	70 (11)	185 (19)
	Good	144 (23)	190 (19)
	Moderate	37 (6)	42 (4)
	Bad	4 (0.6)	3 (0.3)
	Very Bad	0 (0)	0 (0)
	Not asked in survey included	359 (57)	548 (55)
	Missing*	12 (2)	25 (3)
Exclusive breastfeeding to 6 months	Yes	244 (39)	396 (40)
	No	11 (2)	24 (2)
	Not asked in survey included	359 (57)	548 (55)
	Missing*	12 (2)	25 (3)
Family planning advice at last clinic visit	Yes	539 (86)	864 (87)
	No	58 (9)	78 (8)
	Missing*	29 (5)	51 (5)
Current method to prevent pregnancy	None	94 (15)	137 (14)
	NA: pregnant	3 (0.5)	4 (0.4)
	Yes: contraceptive	433 (69)	723 (73)
	Yes: natural methods	57 (9)	49 (5)
	Yes: other	9 (1)	29 (3)
	Missing*	30 (5)	51 (5)

Data shown as *n*, *n* (%) or median (IQR). Data were available for some women for both the planned '6 month survey' and the planned '12 month survey' (although the actual timing was not necessarily as planned), and in these the latter was used if completed 5-18 months after delivery as it contained questions on self-rated maternal health and age at end of exclusive breastfeeding. *Of those women with relevant survey data available.

For peer review only - http://bmjopen.bmj.com/site/about/guidelines.xhtml

Sensitivity analysis

A sensitivity 'per protocol' analysis of the primary outcomes was prespecified to only include those clinics and periods for which payments were being processed. However, as the correct functioning of the payment systems did not follow clear temporal divisions, the sensitivity analysis was conducted including those intervention and control clinics with a proportion of women with prompt payment (within 31 days) of their first ANC visit above the median within each arm.

We evaluated prompt payment from first visit among the 4,156 women with at least one ANC visit included in the primary analysis. There was a prompt payment in 743/2,141 (34.7%) women in the control clinics and in 943/2,015 (46.8%) in the intervention clinics (Figure S1). Across control clinics, the median proportion with prompt payment for first visit was 32.3%, with interquartile range (IQR) 17.8% to 46.1% (Figure S2). Across intervention clinics, the median proportion with prompt payment was 42.4%, with IQR 27.0% to 50.4% (Figure S3).

In the sensitivity analysis, there was no observed positive effect of the implementation on ANC attendance, and the pooled estimate of the intervention effect was negative but not statistically significant (Table S2). As this finding was surprising given the main results, we investigated the cluster-level association between prompt payment and ANC attendance. This revealed a stronger correlation in the control clinics than in the intervention clinics (Figure S4). Consequently, in our sensitivity analysis in terms of ANC attendance rates, we were unexpectedly comparing high performing control clinics with typical intervention clinics, which explains the change to the intervention effect from the main analysis.

Discussion

In this paper, we evaluated the impact of a demand side financing intervention, using CCTs, on the retaining pregnant women in the continuum of care from their first ANC visit until their children reach 1 year of age in rural Kenya. Previous evaluations of CCT programmes in the Sub Saharan Africa region have focussed on either increasing ANC visits[33], institutional deliveries[18, 33] or PNC visits[34]. Two other studies in the region evaluated the impact of demand side financing on the retaining women in the continuum of care. For the continuum of care, one study evaluated the impact of a subsidized reproductive health voucher programme and the introduction of free maternity services in government facilities on ANC visits, facility birth and PNC visits in Kenya[35]. Another study[36] examined the impact of a national CCT pilot programme on the continuum of care in rural Nigeria. To our knowledge, this is the first evaluation of the impact of CCTs on retaining pregnant women in the continuum of care in Kenya and provides crucial evidence to inform policy and practice related to demand side financing mechanisms for improving maternal and new-born health outcomes.

Increased ANC clinic attendance and child immunization appointments

Our main finding suggests that the intervention led to a modest increase in ANC clinic attendance and child immunisation appointments. This is consistent with evidence on the impact of demand side financing programmes on ANC service utilization from the sub–Saharan African region but less so with the evidence on child immunization. For example, a study set in Kenya[35] found that a subsidized reproductive health voucher programme and free maternity services improved early initiation of ANC as well as continuous use of care amongst ANC attendees in government facilities. Others[33, 36] also found that CCT programmes increased ANC attendance in rural Kenya and Nigeria, respectively. This implies that demand side financing interventions, whether using CCTs or vouchers can increase ANC service utilization, though the size of the impact might vary. It is possible that the increase in ANC

For peer review only - http://bmjopen.bmj.com/site/about/guidelines.xhtml

service utilization might be greater if financial incentives such as CCTs and vouchers are combined with other policy measures such as free maternity services.

As this was a cluster RCT, it was possible that knowledge of the incentives spread in the community and women could have attend earlier to collect more incentives. However, the results show that women (in both arms) attended their visit in week 22 on average. According to the WHO recommendations, the first ANC visit should be scheduled between week 8 and 12 of the pregnancy[31]. Late attendance for ANC visits was also found in other studies[32, 37, 38], and the consequences of that late first visit require further investigation, and further research could be done in how to incentivise early attendance.

Limited effect on facility delivery and PNC visits

The Nigerian CCT programme did not find any impacts on neonatal immunization[36]. In Zimbabwe, little improvement was found in immunization amongst children under 5 years of age[34]. This is in contrast with our findings. We did not observe a significant impact on facility delivery, which is consistent with the findings from[36] but not with other studies[18, 39] that found a positive impact of CCTs on facility delivery. We did not find a clear intervention effect on the proportion of women with at least one PNC visit at 4-12 months, but women did have higher numbers of total PNC visits in the intervention clinics. This suggests further research into unpacking why financial incentives do not have as consistent an impact on facility delivery and PNC visits or child immunisation in comparison to the effect on ANC service utilization.

Challenges with the trial

We faced two major and connected challenges related to the technical functioning of the card system and a delay in the transfer of payments, the latter being common for CCT programmes[40]. The touching in of the Afya card reader was intended to record the visit and automatically trigger payments to participants. However, only 26% of payments were triggered automatically (further details in[27]). The remaining transfers required involvement of the field implementation partner to manually record a visit and trigger a transfer. This caused several delays in payments being made to participants, often over months. Other challenges such as healthcare staff not tapping the cards to avoid conflicts with participants over delayed payments, reluctance of new staff at facilities to participate due to challenges with delayed transfers; the card reader being locked by the main staff member actively involved in intervention to avoid theft but limiting use by other healthcare staff contributed to the intervention not being implemented as intended. All these factors, linked to the technology and delay in payments could have diluted the impact of the intervention and prevented service utilization in the latter stages of pregnancy or after babies were born.

Our findings of modest increases in scheduled ANC and immunisation appointment attendance, need to be interpreted in the context of the implementation challenges described, noting that most of these are partly inherent to the intervention we evaluated given the resources available and the trial context. Although implementation of the intervention could have been enhanced for example by either more frequent regular visits to facilities to support clinic staff or by incentivising clinic staff more to process payments throughout a woman's care, these models may be unsustainable at scale. We saw evidence of a modest intervention effect on counts of all attendance types other than delivery when lifting the eligibility requirements for the analysis and thereby potentially including some unscheduled visits and visits additional to the expected maximum required. These findings may reflect in part an increase in visits made without serious health concerns where the cash transfer is a major motivation, though unscheduled visits were not eligible for payments. We conducted a per-protocol sensitivity analysis, but this was ultimately unhelpful since it led to an unfair comparison between

For peer review only - http://bmjopen.bmj.com/site/about/guidelines.xhtml

arms, and so we are unable to quantify the intervention effects that might be expected had implementation been more complete.

Our randomisation process was based on selecting 48 facilities to participate from a shortlist of 60 and simultaneously randomising these to intervention or control. Where the catchment area for a selected facility was found to overlap with a previously selected facility it was replaced. Although we believe the randomisation process was implemented objectively, we acknowledge that there could have been some subjectivity in deciding whether catchment areas overlapped and since the allocation to intervention or control was already revealed at this point it is theoretically possible that bias was introduced.

The collection of outcome data as originally planned was unfeasible and, whilst it is a real strength that we managed to collect ANC attendance data for almost all trial participants, it is a limitation that we managed to collect data on the other primary outcomes for only a minority of women and less commonly in the control arm, and no data on attendance for referrals. The potential bias is however limited to a degree by our approach to analysis in which all outcomes are modelled simultaneously. Although the telephone follow-up surveys proved challenging these data are not central to the analyses and interpretation presented here. We did not recruit to our original sample size target but obtained outcomes from all clusters.

Conclusions

This trial has demonstrated modest benefits from a CCT intervention, that was affected by technical and other implementation challenges. Further research is needed to address how to design a more robust process for registering attendances and ensuring rapid payment of CCTs to ensure women have confidence in receipt of CCTs for future attendances. This could impact incentivise women to attend visits earlier in their pregnancy as well.

Author contribution

FV was the Principal Investigator, leading study implementation from May 2018 to June 2020. OS, TP, and AC were the trial statisticians, with AC leading the design of the data analysis methods and interpretation of the research findings, and OS undertaking the analysis. AO was the Trial Coordinator, leading field implementation under supervision of AM. SD, JS, NB, HHB, TP led specific components of the trial such as the process evaluation and the economic evaluation and contributed to the overall research methods and design. The entire team contributed to the interpretation of the research findings and the writing of the paper. All authors read and approved the final manuscript.

Funding statements

The authors thank the Bill and Melinda Gates Foundation for the funding for this project (grant number: OPP 1142564) and the Swedish International Development Cooperation Agency for providing co-financing (grant number: not applicable). The funders were not involved in the design, implementation, data collection, analysis, writing and decision to submit the paper for publication.

Acknowledgements

The authors thank the following people for their contribution to the trial: Caroline Ochieng, Alie Eleveld, Geordan Shannon, Stacey Noel, Matthew Fielding, Sangoro Onyango and Sarah Odera.

For peer review only - http://bmjopen.bmj.com/site/about/guidelines.xhtml

Competing interest

The authors declare no competing interests, aside from AC who is associate editor of Sexually Transmitted Infections.

Data Availability Statement

De-identified data may be made available upon request to researchers who provide a scientifically and methodologically sound research proposal and obtain ethical approval for their planned analysis. Proposals should be submitted to the corresponding author. Data that can be shared includes number of visits made, type of visit, arm, and date of the visit. Data dictionaries can be made available, as well as the study protocol and the statistical analysis plan. Data are available now.

Figure legend

Figure 1. Flow diagram of enrolment and inclusion in analyses by clinic randomization status. n values refer to women and n_c to clinics. EDD expected date of delivery, ADD actual date of delivery.

For peer review only

References

1. WHO. TRENDS IN MATERNAL MORTALITY: 2000 TO 2017. Estimates by WHO, UNICEF, UNFPA, World Bank Group and the United Nations Population Division [Internet]. Geneva; 2019. Available from: https://www.unfpa.org/sites/default/files/pub-pdf/Maternal_mortality_report.pdf

2. Kenya National Bureau of Statistics. Kenya Demographic and Health Survey 2014 - Key Indicators [Internet]. Nairobi, Kenya; 2015 Mar. Available from: https://www.dhsprogram.com/pubs/pdf/sr227/sr227.pdf

3. UNICEF. Neo-natal mortality and under-five mortality [Internet]. 2020 Sep. Available from: https://data.unicef.org/topic/child-survival/under-five-mortality/#:~:text=Sub%2DSaharan%20Africa%20continues%20to,deaths%20per%201%2C000%20live%20births.

4. Say L, Chou D, Gemmill A, Tunçalp Ö, Moller A-B, Daniels J, et al. Global causes of maternal death: a WHO systematic analysis. Lancet Glob Health. 2014 Jun;2(6):e323–33.

5. UN IGME. Levels & Trends in Child Mortality. Report 2018 [Internet]. UNICEF; 2018. Available from: https://childmortality.org/reports

6. Campbell OM, Graham WJ. Strategies for reducing maternal mortality: getting on with what works. The Lancet. 2006 Oct;368(9543):1284–99.

7. Carroli G, Rooney C, Villar J. How effective is antenatal care in preventing maternal mortality and serious morbidity? An overview of the evidence. Paediatr Perinat Epidemiol. 2001 Jan;15(s1):1–42.

8. Barber SL, Gertler PJ. Empowering women: how Mexico's conditional cash transfer programme raised prenatal care quality and birth weight. J Dev Eff. 2010 Apr 13;2(1):51–73.

9. Okeke EN, Chari AV. Health care at birth and infant mortality: Evidence from nighttime deliveries in Nigeria. Soc Sci Med. 2018 Jan;196:86–95.

10. Daysal NM, Trandafir M, van Ewijk R. Saving Lives at Birth: The Impact of Home Births on Infant Outcomes. Am Econ J Appl Econ. 2015 Jul;7(3):28–50.

11. Warren C, Mwangi A, Oweya E, Kamunya R, Koskei N. Safeguarding maternal and newborn health: improving the quality of postnatal care in Kenya. Int J Qual Health Care. 2010 Feb 1;22(1):24–30.

12. Kerber KJ, de Graft-Johnson JE, Bhutta ZA, Okong P, Starrs A, Lawn JE. Continuum of care for maternal, newborn, and child health: from slogan to service delivery. The Lancet. 2007 Oct;370(9595):1358–69.

13. WHO. Factsheet Maternal Mortality [Internet]. 2018 Feb. Available from: https://www.who.int/news-room/fact-sheets/detail/maternal-mortality

14. Pell C, Meñaca A, Were F, Afrah NA, Chatio S, Manda-Taylor L, et al. Factors Affecting Antenatal Care Attendance: Results from Qualitative Studies in Ghana, Kenya and Malawi. PLOS ONE. 2013 Jan 15;8(1):e53747.

For peer review only

For peer review only - http://bmjopen.bmj.com/site/about/guidelines.xhtml

15. Kenya National Bureau of Statistics (KNBS), ICF Macro. Kenya Demographic and Health Survey 2008-09 [Internet]. Calverton, Maryland; 2010. Available from: https://dhsprogram.com/pubs/pdf/fr229/fr229.pdf

16. Lund S, Nielsen BB, Hemed M, Boas IM, Said A, Said K, et al. Mobile phones improve antenatal care attendance in Zanzibar: a cluster randomized controlled trial. BMC Pregnancy Childbirth. 2014 Jan 17;14(1):29.

17. Kebede AS, Ajayi IO, Arowojolu AO. Effect of enhanced reminders on postnatal clinic attendance in Addis Ababa, Ethiopia: a cluster randomized controlled trial. Glob Health Action. 2019 May 24;12(1):1609297.

18. Grépin KA, Habyarimana J, Jack W. Cash on delivery: Results of a randomized experiment to promote maternal health care in Kenya. J Health Econ. 2019 May 1;65:15–30.

19. Okeke EN, Abubakar IS. Healthcare at the beginning of life and child survival: Evidence from a cash transfer experiment in Nigeria. J Dev Econ. 2020 Mar 1;143:102426.

20. Hurst TE, Semrau K, Patna M, Gawande A, Hirschhorn LR. Demand-side interventions for maternal care: evidence of more use, not better outcomes. BMC Pregnancy Childbirth. 2015 Nov 13;15:297.

21. Ekezie CC, Lamont K, Bhattacharya S. Are Cash Transfer Programs Effective in Improving Maternal and Child Health in Sub-Saharan Africa? A Systematic Review of Randomized Controlled Trials. J Glob Health Columbia Univ [Internet]. 2017 May 21 [cited 2020 Nov 27];7(1). Available from: https://journals.library.columbia.edu/index.php/jgh/article/view/5012

22. Glassman A, Duran D, Fleisher L, Singer D, Sturke R, Angeles G, et al. Impact of conditional cash transfers on maternal and newborn health. J Health Popul Nutr. 2013 Dec;31(4 SUPPL.2):S48–66.

23. Hunter BM, Harrison S, Portela A, Bick D. The effects of cash transfers and vouchers on the use and quality of maternity care services: A systematic review. PLOS ONE. 2017 Mar 22;12(3):e0173068.

24. Lagarde M, Haines A, Palmer N. Conditional cash transfers for improving uptake of health interventions in low- and middle-income countries: a systematic review. JAMA. 2007 Oct 24;298(16):1900–10.

25. Ochieng CA, Haghparast-Bidgoli H, Batura N, Odhiambo A, Shannon G, Copas A, et al. Conditional cash transfers to retain rural Kenyan women in the continuum of care during pregnancy, birth and the postnatal period: protocol for a cluster randomized controlled trial. Trials. 2019 Mar 1;20(1):152.

26. Fielding M, Ochieng C, Odero B. From paper to data: taking medical health records into the future. 2016;7.

27. Dickin S, Vanhuyse F, Odhiambo A, Stirrup O, Liera C, Copas A, et al. Afya health credit incentive: a process evaluation. forthcoming.

For peer review only - http://bmjopen.bmj.com/site/about/guidelines.xhtml

28. Craig P, Dieppe P, Macintyre S, Michie S, Nazareth I, Petticrew M. Developing and evaluating complex interventions [Internet]. London, UK: Medical Research Council; 2019. Available from: https://mrc.ukri.org/documents/pdf/complex-interventions-guidance/

29. Palmer T, Batura N, Skordis J, Stirrup O, Copas A, Dickin S, et al. Cost-effectiveness of conditional cash transfers to retain women in the continuum of care during pregnancy, birth and the postnatal period: an economic evaluation of the Afya trial in Kenya. In 2021.

30. Waithaka D, Kagwanja N, Nzinga J, Tsofa B, Leli H, Mataza C, et al. Prolonged health worker strikes in Kenya- perspectives and experiences of frontline health managers and local communities in Kilifi County. Int J Equity Health. 2020 Feb 10;19(1):23.

31. WHO. WHO recommendations on antenatal care for a positive pregnancy experience. WHO; 2016.

32. Sarker BK, Rahman M, Rahman T, Rahman T, Khalil JJ, Hasan M, et al. Status of the WHO recommended timing and frequency of antenatal care visits in Northern Bangladesh. PLOS ONE. 2020 Nov 5;15(11):e0241185.

33. Kahn C, Iraguha M, Baganizi M, Kolenic GE, Paccione GA, Tejani N. Cash Transfers to Increase Antenatal Care Utilization in Kisoro, Uganda: A Pilot Study. Afr J Reprod Health. 2015 Sep;19(3):144–50.

34. Robertson L, Mushati P, Eaton JW, Dumba L, Mavise G, Makoni J, et al. Effects of unconditional and conditional cash transfers on child health and development in Zimbabwe: a cluster-randomised trial. The Lancet. 2013 Apr 13;381(9874):1283–92.

35. Dennis ML, Benova L, Abuya T, Quartagno M, Bellows B, Campbell OMR. Initiation and continuity of maternal healthcare: examining the role of vouchers and user-fee removal on maternal health service use in Kenya. Health Policy Plan. 2019 Mar 1;34(2):120–31.

36. Okoli U, Morris L, Oshin A, Pate MA, Aigbe C, Muhammad A. Conditional cash transfer schemes in Nigeria: potential gains for maternal and child health service uptake in a national pilot programme. BMC Pregnancy Childbirth. 2014 Dec 12;14(1):408.

37. Gebresilassie B, Belete T, Tilahun W, Berhane B, Gebresilassie S. Timing of first antenatal care attendance and associated factors among pregnant women in public health institutions of Axum town, Tigray, Ethiopia, 2017: a mixed design study. BMC Pregnancy Childbirth. 2019 Sep 18;19(1):340.

38. Jiwani SS, Amouzou-Aguirre A, Carvajal L, Chou D, Keita Y, Moran AC, et al. Timing and number of antenatal care contacts in low and middle-income countries: Analysis in the Countdown to 2030 priority countries. J Glob Health. 2020;10(1):010502.

39. Powell-Jackson T, Mazumdar S, Mills A. Financial incentives in health: New evidence from India's Janani Suraksha Yojana. J Health Econ. 2015 Sep 1;43:154–69.

40. NGEC. Participation of vulnerable populations in their own programme: the cash transfers in Kenya [Internet]. 2014. Available from: https://www.ngeckenya.org/Downloads/cash-transfer-programme-vulnerable-groups-kenya.pdf

For peer review only - http://bmjopen.bmj.com/site/about/guidelines.xhtml

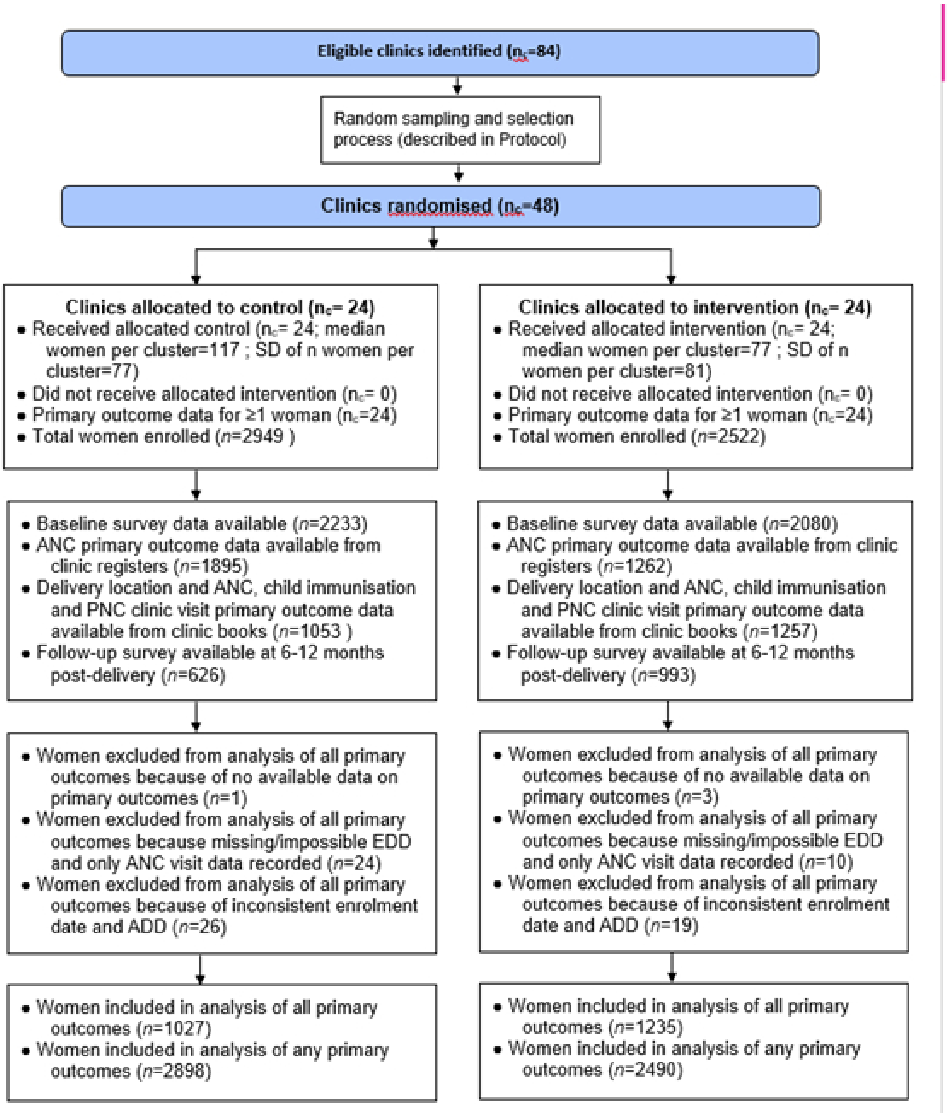

Flow diagram of enrolment and inclusion in analyses by clinic randomization status. n values refer to women and nc to clinics. EDD expected date of delivery, ADD actual date of delivery.

155x171mm (96 x 96 DPI)

Figure S1 Success or failure of prompt payment in individual women (● at 1 or 0), the overall success proportion (blue line) and a 31-day moving average (red line) divided by control and intervention clinics.

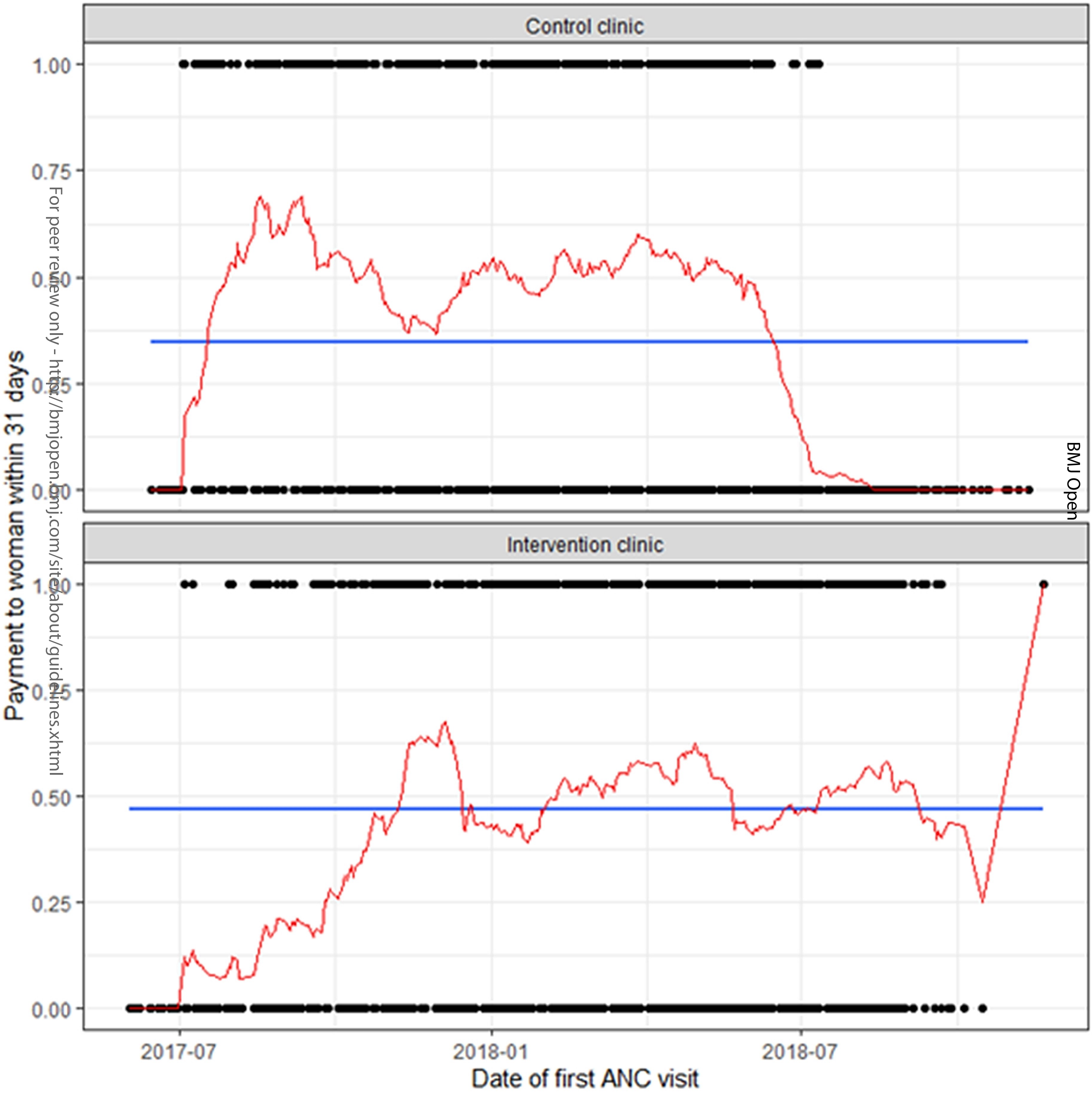

For peer review only - http://bmjopen.bmj.com/site/about/guidelines.xhtml

Figure S2 Success or failure of prompt payment in individual women (● at 1 or 0), the overall success proportion (blue line) and a 31-day moving average (red line) in individual control clinics.

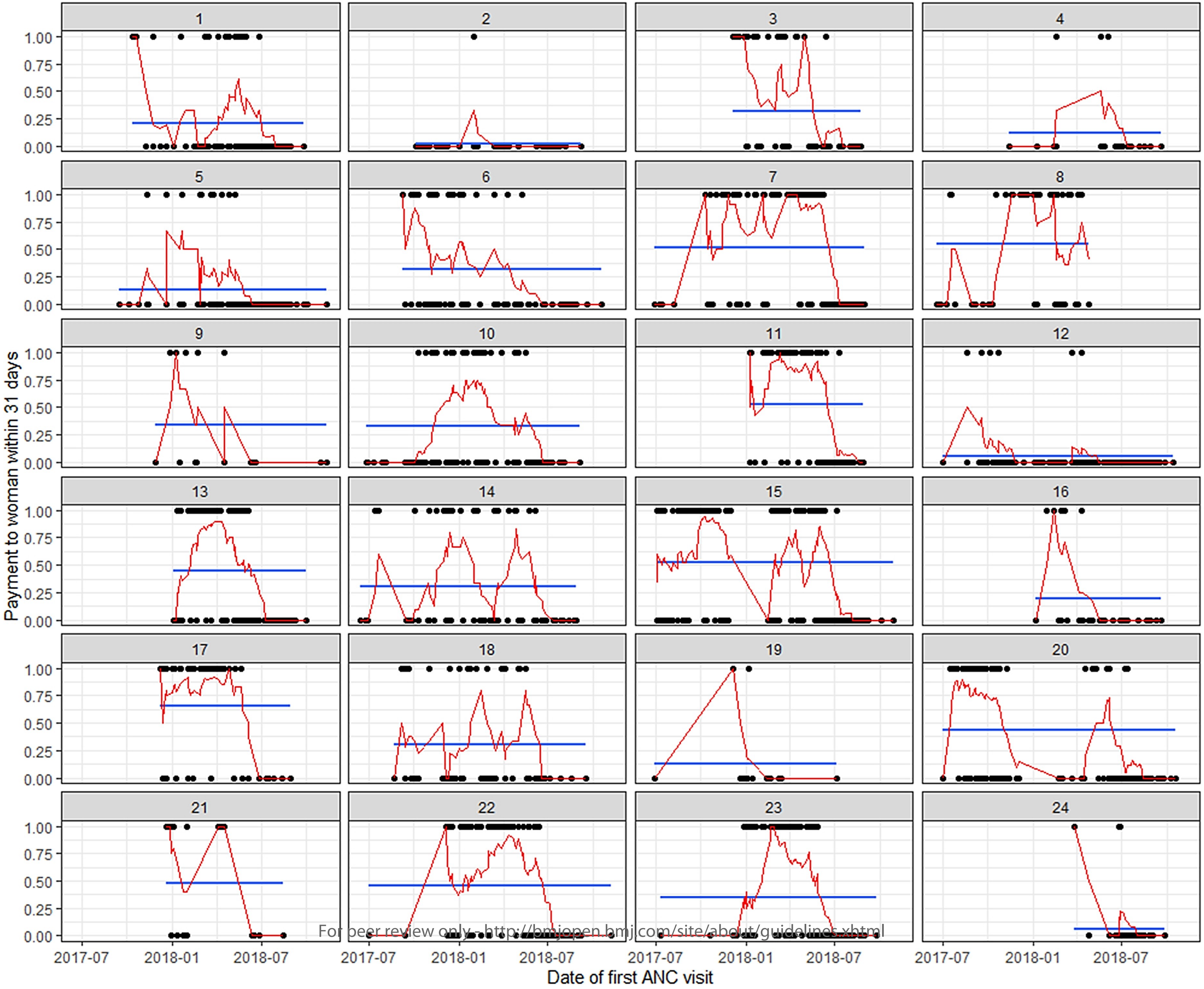

Figure S3 Success or failure of prompt payment in individual women (● at 1 or 0), the overall success proportion (blue line) and a 31-day moving average (red line) in individual intervention clinics.

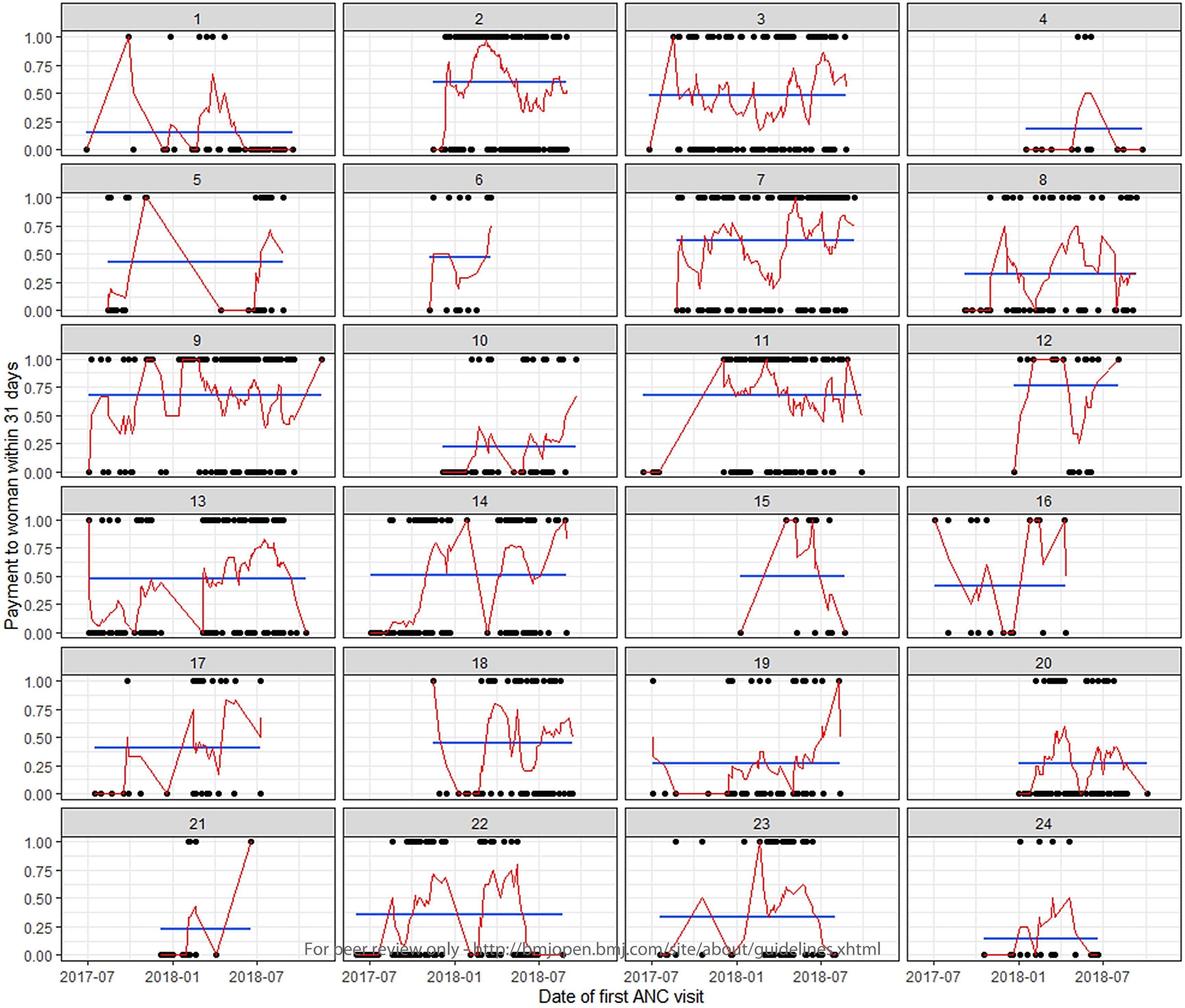

For peer review only - http://bmjopen.bmj.com/site/about/guidelines.xhtml

Figure S4 Exploratory analysis of the clinic-level relationship between proportion of women paid within 31 days of their first ANC clinic visit and the overall attendance proportion for eligible ANC visits. The graph is divided according to control and intervention clinics, with separate linear regression line (blue, with shaded 95%CI) and median percentage women with prompt payment (vertical black line) for each group.

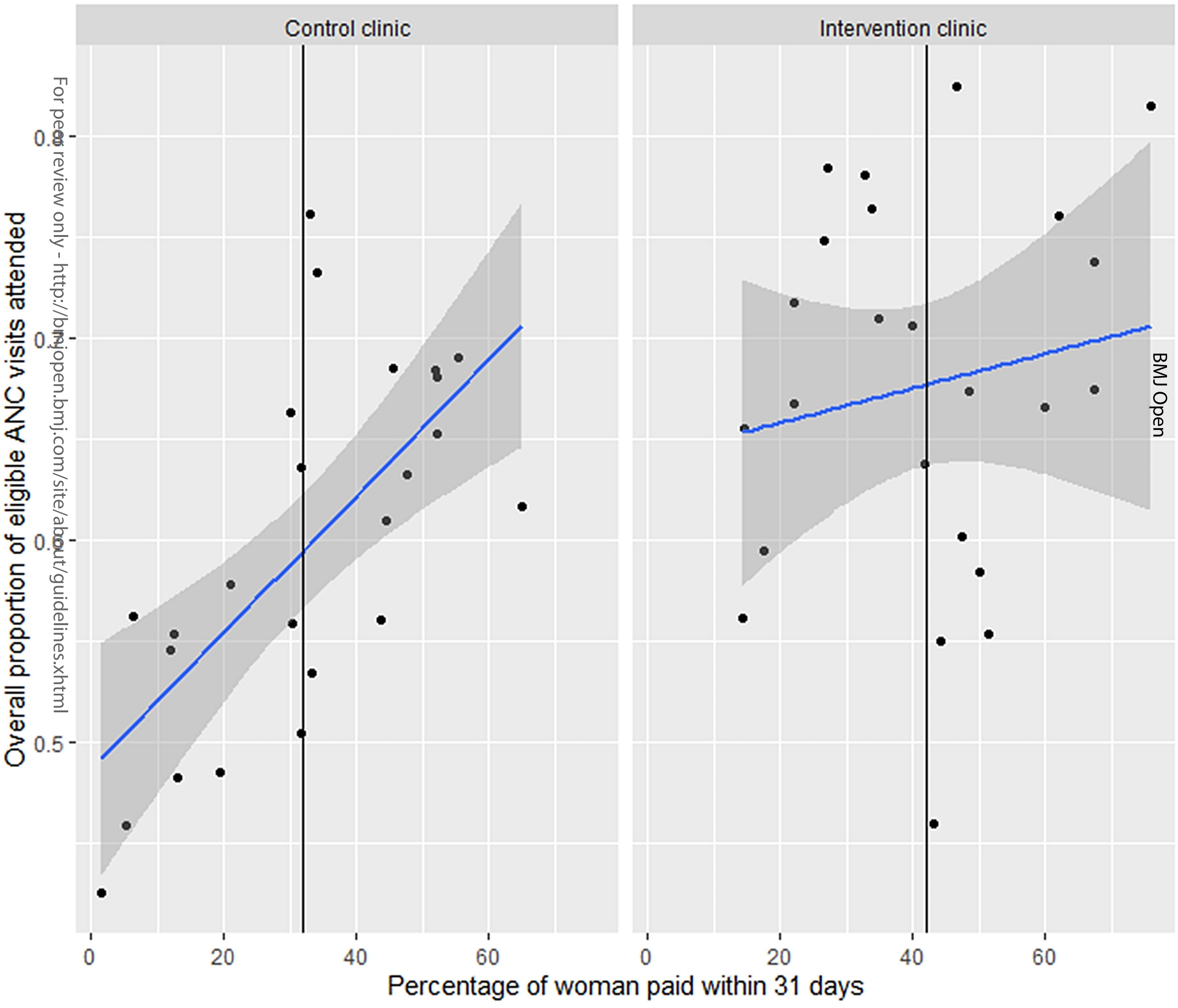

For peer review only - http://bmjopen.bmj.com/site/about/guidelines.xhtml

Supplementary material

Appendix S1 – Differences from published protocol in data collection and analysis

This summary of differences between the trial protocol and the data collection and analysis that were conducted is closely based on that in the statistical analysis plan (SAP) for the study, which was written prior to unblinding and analysis of data by the trial statistician.

The published Protocol for the study stated that the primary outcomes for the study would be derived by combining data from manual extraction of clinic health records and from the electronic card reading system used to process the trial payments. However, due to technical issues in implementation of the card reading system, it was not possible to extract outcome data from this source. As such, outcome data were also obtained from the personal clinic books of women participating in the trial. Specifically, participants were invited to attend the clinic shortly after the end of the trial with their clinic book for collection of the primary outcomes. For women who however did not attend the clinic for that purpose, data were extracted from the clinic register. Only very limited data on any treatment referrals beyond routine visits were collected and as such this primary outcome (5 of 5 in the Protocol) was dropped from the planned analysis. In addition, there were only data available for the primary outcomes of delivery at a healthcare facility and child immunisation and maternal PNC visits for those women with clinic book available for data extraction (only antenatal visits were extracted from clinic registers).

It was planned that data for the secondary outcomes would be derived from clinic records, the electronic card reading system and a series of four follow-up telephone interviews (after enrolment in all women; 2 weeks after delivery in all women who do not give birth in a facility; 6 months after EDD in 50% of women; 12 months after delivery in the same 50% of women). However, as well as issues with the card reader system, the interviews at 6 and 12 months after delivery were combined into one survey which was primarily delivered towards 12 months after delivery, after some 6-month interviews had already been conducted. The following secondary outcomes were dropped as data were not collected: mother's perception of infant health at 6 and 12 months post-delivery, screening and control of infections for mothers and foetus/baby during pregnancy and postnatal periods.

Perinatal and maternal mortality were recorded if a woman (or family member) attended with their clinic book for data extraction, but completeness of the reporting has not been verified. The remaining outcomes were collected as part of the 6–12-month survey but the survey completion rate was ultimately relatively low, and as such, we have reported available data for the specified secondary outcomes at 6-12 months but have not carried out any formal statistical analyses. The completion rate of the baseline survey was high, but not sufficiently high to use these data to adjust our analyses for maternal characteristics such as socioeconomic factors.

Additional secondary outcomes of the counts of ANC, child immunisation and PNC clinic visits (both with and without applying any eligibility criteria regarding the timing of visits or maximum for each type) per woman were analysed and reported; this was not listed in the original Protocol, where the focus was on the primary outcomes of the *proportion* of each appointment type attended. Analysing the total counts in each case also allows for capture of the potential impact of earlier commencement of ANC care, and creates model outputs useful for health economic analysis. A further additional secondary outcome of GA at enrolment to the trial was also added, to evaluate whether the trial intervention encouraged earlier engagement with antenatal care (whereas the primary outcomes only relate to events following enrolment of each woman). This outcome was available for a large majority of women enrolled in the study.

For peer review only - http://bmjopen.bmj.com/site/about/guidelines.xhtml

The published Protocol stated that the analysis of primary outcomes would use logistic regression for binary outcomes and ordinal regression for ordinal outcomes, with a single pooled effect estimate for the intervention across these outcomes estimated using independence estimating equations. However, since delivery and postnatal outcome data were missing in a substantial proportion of participants, we planned to allow for dependence (i.e., correlation) between each of the outcome variables for each woman using structured random effects models. To facilitate this, visit counts for each woman were analysed as repeated binary observations rather than as ordinal variables.

Appendix S2 – Summary of available mortality data

These data have been obtained from both free-text notes in the study visit records and from information collected in the telephone surveys. It is therefore difficult to gauge the level of ascertainment of these adverse outcomes and the level of completeness will also depend on engagement with care and follow-up, which differed between the control and intervention groups.

Among the 2,949 women enrolled into the control arm, there was one record of intrauterine death of the foetus, one record of a stillbirth, 17 records of neonatal deaths (immediately or up to 1 week following delivery), 33 records of infant deaths up to 18 months after delivery and no records of maternal deaths.

Among the 2,522 women enrolled into the intervention arm, there were two records of intrauterine deaths of the foetus, three records of stillbirth, 23 records of neonatal deaths (immediately or up to 1 week following delivery), 45 records of infant deaths up to 18 months after delivery and two records of maternal deaths. The maternal deaths were both recorded at data extraction from maternal clinic books and appear to have occurred within a year of delivery, but not in the immediate neonatal period.

For peer review only

For peer review only - http://bmjopen.bmj.com/site/about/guidelines.xhtml

Additional Tables

Table S1: Cluster-level summaries of the enrolled women included in the primary analysis

Variable	Control clinic	Intervention clinic	ICC
N women per cluster in analysis	113 (67-174;21-301)	77 (31-158, 17-313)	—
**Baseline characteristics**			
Median age	26 (25-27, 22-29)	26 (25-27, 23-30)	—
Proportion nulliparous	0.22 (0.16-0.28, 0.004-0.37)	0.19 (0.10-0.26, 0-0.38)	—
Median GA at enrolment (days)	158 (151-166, 136-179)	153 (149-159, 139-183)	—
Proportion high-risk pregnancies	0.20 (0.11-0.24, 0.06-1)	0.17 (0.14-0.38, 0.06-0.94)	—
**Primary outcomes**			
Mean proportion attendance at eligible ANC clinic appointments (following scheduled visits)	0.61 (0.54-0.67, 0.48-0.75)	0.70 (0.65-0.75, 0.50-0.82)	0.028*
Proportion delivery at health facility	0.92 (0.89-0.96, 0.83-1)	0.90 (0.86-0.94,0.71-1)	0.012†
Proportion attendance eligible PNC clinic appointment (at least one 4-12mo)	0.85 (0.76-0.90, 0.49-1)	0.85 (0.77-0.90, 0.47-0.94)	0.087†
Mean proportion attendance at child immunisation appointments (capped at 4)	0.85 (0.79-0.90, 0.71-1)	0.89 (0.86-0.91, 0.76-0.98)	0.011*

Data shown as median (interquartile range, range) by cluster.
ICC values calculated as $(var(b_i))/(var(b_i)+var(e_{ij}))$ from a linear mixed model with adjustment for intervention and subcounty, where $var(b_i)$ is cluster-level random intercept variance and $var(e_{ij})$ is the residual variance. *Linear mixed model fitted to overall proportion of visits attended as outcome for each woman. †Linear mixed model fitted to binary outcome data (i.e., 0 for no visit and 1 for attendance).

For peer review only

Table S2: Effect of conditional cash transfers on primary outcome measures for the sensitivity analysis only including the 12 intervention clinics and the 12 control clinics with the highest proportion of enrolled women with payment within 31 days of first ANC clinic visit.

	**Control clinic**	**Intervention clinic**		
**Primary outcome measures**	***n/N* (%)**	***n/N* (%)**	**OR (95% CI)**	**aOR (95% CI); *P***
Attendance at eligible ANC clinic appointments (following scheduled visits)*	3491/5335 (65)	3720/5775 (64)	0.78 (0.55-1.11)	0.80 (0.55-1.17); *P*=0.25
Delivery at health facility†	575/624 (92)	754/838 (90)	0.31 (0.11-0.88)	0.32 (0.11-0.92); *P*=0.04
Attendance at eligible PNC clinic appointment (at least one 4-12mo)‡	503/624 (81)	680/835 (81)	0.88 (0.45-1.74)	0.92 (0.47-1.82); *P*=0.81
Attendance at child immunisation appointments (capped at 4)†	2109/2496 (84)	2960/3352 (88)	1.65 (0.89-3.05)	1.72 (0.93-3.18); *P*=0.08
Pooled intervention estimate	—	—	0.89 (0.65-1.22)	0.92 (0.66-1.27); *P*=0.60

aOR, adjusted odds ratio; OR, odds ratio. *Available for all women included in primary analysis except three (with data from clinic book but no available EDD or ADD). †Available for 1462/3279 women with data obtained from clinic books. ‡Available for 1459 women with data obtained from clinic books and with available ADD or EDD. Available data for attendances and eligible appointments are summed over all women for the *n*/N values.

For peer review only

Table 1: CONSORT 2010 checklist of information to include when reporting a cluster randomised trial

Section/Topic	Item No	Standard Checklist item	Extension for cluster designs	Page No *
**Title and abstract**				1-2
	1a	Identification as a randomised trial in the title	Effectiveness of conditional cash transfers (Afya credits incentive) to retain women in the continuum of care during pregnancy, birth and the postnatal period in Kenya: a cluster randomised trial	1
	1b	Structured summary of trial design, methods, results, and conclusions (for specific guidance see CONSORT for abstracts)[1,2]	Objectives<br><br>Given high maternal and child mortality rates, we assessed the impact of Conditional Cash Transfers (CCTs) to retain women in the continuum of care (antenatal care (ANC), delivery at facility, postnatal care (PNC) and child immunization).<br><br>Design<br><br>We conducted an unblinded 1:1 cluster-randomized controlled trial.<br><br>Setting<br><br>48 health facilities in Siaya County, Kenya were randomized. The trial ran from May 2017 to December 2019.<br><br>Participants<br><br>2922 women were recruited to the control and 2522 to the intervention arm.	2

For peer review only - http://bmjopen.bmj.com/site/about/guidelines.xhtml

Interventions

An electronic system recorded attendance and triggered payments to the participant's mobile for the intervention arm (4.5 USD), and phone credit for the control arm (0.5 USD). Eligibility criteria were resident in the catchment area and access to a mobile phone.

Primary outcomes

Primary outcomes were any ANC, delivery, any PNC between 4- 12 months after delivery, childhood immunization, and referral attendance to other facilities for ANC or PNC. Given problems with the electronic system, primary outcomes were obtained from maternal clinic books if participants brought them to data extraction meetings (1257 (50%) of intervention and 1053 (36%) control arm participants). Attendance at referrals to other facilities is not reported because of limited data.

Results

We found a significantly higher proportion of appointments attended for ANC (67% vs. 60%, adjusted OR (aOR) 1.90; 95% CI 1.36-2.66) and child immunization (88 vs 85%; aOR 1.74; 95% CI 1.10-2.77) in intervention than control arm. No intervention effect was seen considering delivery at the facility (90 vs 92%; aOR 0.58; 95% CI 0.25-1.33) and any PNC attendance (82 vs 81%; aOR 1.25; 95% CI 0.74-2.10) separately. The pooled odds ratio across all attendance types was 1.64 (1.28-2.10).

			Conclusions<br><br>Demand-side financing incentives, such as CCTs, can improve attendance for appointments. However, attention needs to be paid to the technology, the barriers that remain for delivery at facility and PNC visits and encouraging women to attend ANC visits within the recommended WHO timeframe.	
**Introduction**				3
**Background and objectives**	2a	Scientific background and explanation of rationale	Rationale for using a cluster design	4
	2b	Specific objectives or hypotheses	Whether objectives pertain to the cluster level, the individual participant level or both	4
**Methods**				
**Trial design**	3a	Description of trial design (such as parallel, factorial) including allocation ratio	The units of randomisation were Level 2 or 3 health facilities (Dispensaries and Health Centres, respectively). The randomisation of facilities was stratified by the six sub-Counties and ensured equal allocation to study arms within each stratum without any overlap of catchment areas,, as described in detail in the trial protocol[25].	3-4
	3b	Important changes to methods after trial commencement (such as eligibility criteria), with reasons		**5-6; 13-14**
**Participants**	4a	Eligibility criteria for participants	Criteria for enrolment were women attending their first ANC visit; long-term resident of the catchment area served by the health facility (living in the area for at least 6 months); access to a mobile phone that	4

For peer review only

			belongs either to themselves or to a member of their household or person whom they trust.	
	4b	Settings and locations where the data were collected	48 Level 2 or 3 health facilities (Dispensaries and Health Centres, respectively) in Siaya County, Kenya	**4**
**Interventions**	5	The interventions for each group with sufficient details to allow replication, including how and when they were actually administered	For each scheduled health visit made following enrolment, women in the intervention arm received a cash transfer of KSH 450 (4.5 USD) on their mobile phones. Women at the control clinics were granted KSH 50 (0.5 USD) mobile phone airtime for each scheduled visit to encourage them to bring their clinic booklet to appointments. In both trial arms, women were issued with a trial card at recruitment and at all facilities there was a card reader, which provided the connection between the trial card and an online portal which stored participants' data on visits and payments. Payments to the women were triggered by tapping the card on a card reader, which also logged the visit in an online portal[26]. In the event of problems with the card reader, or if the woman did not bring her card to the appointment, payments could alternatively be processed manually by contacting the implementing partner: once the visit was verified with the facility, the implementing partner entered the visit data in the portal, which would then trigger a payment as well Nurses were given KSH 400 (4 USD) per woman enrolled during the trial, and an additional KSH 100 (1USD) per woman enrolled at the end of the trial for their collaboration in the trial. These payments were transferred to the nurses electronically. Details of the	4

For peer review only

			intervention design are presented in the protocol[25].	
**Outcomes**	6a	Completely defined pre-specified primary and secondary outcome measures, including how and when they were assessed	The primary outcomes were: 1) attendance or missed attendance at each eligible ANC appointment after recruitment; 2) delivery at a health facility; 3) attendance for at least one PNC appointment between 4 and 12 months after delivery; 4) attendance or missed attendance at each expected child immunization appointment; 5) attendance at referrals to other facilities for ANC, PNC or child immunization. The following secondary outcomes are also reported and analysed according to trial arm: 1) attendance of all eligible maternal, new-born and child health (MNCH) care visits, both prenatal and postnatal, for each woman; 2) the count of attended ANC and child immunisation clinic visits eligible for the primary outcome variables for each woman; 3) the total number of ANC, child immunisation and PNC clinic visits (without applying any eligibility criteria) for each woman; 4) gestational age (GA) at first ANC visit (and enrolment to study).	5
	6b	Any changes to trial outcomes after the trial commenced, with reasons		**5-6; 13-14**
**Sample size**	7a	How sample size was determined	Method of calculation, number of clusters(s) (and whether equal or unequal cluster sizes are assumed), cluster size, a coefficient of intracluster correlation (ICC or *k*), and an indication of its uncertainty	6
	7b	When applicable, explanation of any interim analyses and stopping guidelines		

**Randomisation:**				4
**Sequence generation**	8a	Method used to generate the random allocation sequence	In summary, at a public forum with the county government early 2016, the implementing partner wrote the names of 60 shortlisted facilities on pieces of paper and folded them to hide the names, then included them in transparent boxes, one for each sub-county. Each subcounty had an (even) number of facilities to recruit to the trial proportional to subcounty size. The health management teams from each subcounty selected the pieces of paper, one by one. The first was allocated to intervention, second to control. For each selected facility, county officials from the selected subcounty mapped the location and catchment area of the facility on a large map of the county. If a subsequently selected facility had an overlapping catchment area with a previously selected facility, the newly selected facility was rejected, and another drawn to take its place. This process continued until 48 facilities were selected and allocated for the trial.	**4**
	8b	Type of randomisation; details of any restriction (such as blocking and block size)	The randomisation of facilities was stratified by the six sub-Counties and ensured equal allocation to study arms within each stratum without any overlap of catchment areas, as described in detail in the trial protocol[25].	4
**Allocation concealment mechanism**	9	Mechanism used to implement the random allocation sequence (such as sequentially numbered containers), describing any steps taken to conceal the sequence until interventions were assigned	Specification that allocation was based on clusters rather than individuals and whether allocation concealment (if any) was at the cluster level, the individual participant level or both	4

For peer review only - http://bmjopen.bmj.com/site/about/guidelines.xhtml

**Implementation**	10	Who generated the random allocation sequence, who enrolled participants, and who assigned participants to interventions	Replace by 10a, 10b and 10c	4
	10a		Who generated the random allocation sequence, who enrolled clusters, and who assigned clusters to interventions	4
	10b		Mechanism by which individual participants were included in clusters for the purposes of the trial (such as complete enumeration, random sampling)	4
	10c		Oral informed consent was asked in the local language, and then written down on the participant's enrolment form. Refusals were recorded.	4
**Blinding**	11a	If done, who was blinded after assignment to interventions (for example, participants, care providers, those assessing outcomes) and how	Described in reference 25 – trial protocol	
	11b	If relevant, description of the similarity of interventions		
**Statistical methods**	12a	Statistical methods used to compare groups for primary and secondary outcomes	See section "statistical analysis"	7-8
	12b	Methods for additional analyses, such as subgroup analyses and adjusted analyses	See section "statistical analysis"	**7-8**
**Results**				8-12

For peer review only - http://bmjopen.bmj.com/site/about/guidelines.xhtml

**Participant flow (a diagram is strongly recommended)**	13a	For each group, the numbers of participants who were randomly assigned, received intended treatment, and were analysed for the primary outcome	See Figure 1	8, 15
	13b	For each group, losses and exclusions after randomisation, together with reasons	See Figure 1	8, 15
**Recruitment**	14a	Dates defining the periods of recruitment and follow-up	The trial was conducted in 24 intervention and 24 control clinics and enrolled a total of 2522 women at intervention clinics and 2949 at control clinics over a period from May 2017 to December 2019 (Figure 1)	**8**
	14b	Why the trial ended or was stopped		**8**
**Baseline data**	15	A table showing baseline demographic and clinical characteristics for each group	Table 1 and Table 2	8-9
**Numbers analysed**	16	For each group, number of participants (denominator) included in each analysis and whether the analysis was by original assigned groups	Figure 1 + Table 1 and 2	8-9
**Outcomes and estimation**	17a	For each primary and secondary outcome, results for each group, and the estimated effect size and its precision (such as 95% confidence interval)	Table 3 and Table 4	9-11
	17b	For binary outcomes, presentation of both absolute and relative effect sizes is recommended	Table 3 and Table 4	**9-11**
**Ancillary analyses**	18	Results of any other analyses performed, including subgroup analyses and adjusted	See section sensitivity analysis	**11-12**

		analyses, distinguishing pre-specified from exploratory		
**Harms**	19	All important harms or unintended effects in each group (for specific guidance see CONSORT for harms[3])		**11-12**
**Discussion**				12-14
**Limitations**	20	Trial limitations, addressing sources of potential bias, imprecision, and, if relevant, multiplicity of analyses	• Technical issues with the electronic system and at times low participation of health workers resulted in many visits not being registered, and only 26% of the payments triggered automatically.<br>• Manual payments needed to be triggered, which resulted in delays.<br>• This delay in payment could have diluted the impact of the intervention and prevented service utilization in the latter stages of pregnancy or after babies were born.<br>• As manual data abstraction from clinic registers and from the women's maternal clinic books was necessary, we obtained near complete data for ANC attendance but limited data on facility delivery, PNC and child immunization and no data on referral attendance.<br>• The potential bias is however limited to a degree by our approach to analysis in which all outcomes are modelled simultaneously.	**2; 13-14**

For peer review only

**Generalisability**	21	Generalisability (external validity, applicability) of the trial findings	Generalisability to clusters and/or individual participants (as relevant)	11-12
**Interpretation**	22	Interpretation consistent with results, balancing benefits and harms, and considering other relevant evidence		**11-12**
**Other information**				
**Registration**	23	Registration number and name of trial registry	NCT03021070; clinicalTrials.gov	**2**
**Protocol**	24	Where the full trial protocol can be accessed, if available	Ochieng CA, Haghparast-Bidgoli H, Batura N, Odhiambo A, Shannon G, Copas A, et al. Conditional cash transfers to retain rural Kenyan women in the continuum of care during pregnancy, birth and the postnatal period: protocol for a cluster randomized controlled trial. Trials. 2019 Mar 1;20(1):152.	**3; reference 25**
**Funding**	25	Sources of funding and other support (such as supply of drugs), role of funders	The authors thank the Bill and Melinda Gates Foundation for the funding for this project (grant number: OPP 1142564) and the Swedish International Development Cooperation Agency for providing co-financing (grant number: not applicable). The funders were not involved in the design, implementation, data collection, analysis, writing and decision to submit the paper for publication.	**14**

** Note: page numbers optional depending on journal requirements*

For peer review only

Table 2: Extension of CONSORT for abstracts[1,2] to reports of cluster randomised trials

Item	Standard Checklist item	Extension for cluster trials
**Title**	Identification of study as randomised	**Identification of study as cluster randomised**
**Trial design**	Description of the trial design (e.g. parallel, cluster, non-inferiority)	
**Methods**		
**Participants**	Eligibility criteria for participants and the settings where the data were collected	**Eligibility criteria for clusters**
**Interventions**	Interventions intended for each group	
**Objective**	Specific objective or hypothesis	**Whether objective or hypothesis pertains to the cluster level, the individual participant level or both**
**Outcome**	Clearly defined primary outcome for this report	**Whether the primary outcome pertains to the cluster level, the individual participant level or both**
**Randomization**	How participants were allocated to interventions	**How clusters were allocated to interventions**
**Blinding (masking)**	Whether or not participants, care givers, and those assessing the outcomes were blinded to group assignment	
**Results**		
**Numbers randomized**	Number of participants randomized to each group	**Number of clusters randomized to each group**
**Recruitment**	Trial status[1]	

[1] Relevant to Conference Abstracts

For peer review only - http://bmjopen.bmj.com/site/about/guidelines.xhtml

**Numbers analysed**	Number of participants analysed in each group	**Number of clusters analysed in each group**
**Outcome**	For the primary outcome, a result for each group and the estimated effect size and its precision	**Results at the cluster or individual participant level as applicable for each primary outcome**
**Harms**	Important adverse events or side effects	
**Conclusions**	General interpretation of the results	
**Trial registration**	Registration number and name of trial register	
**Funding**	Source of funding	

For peer review only

REFERENCES

[1] Hopewell S, Clarke M, Moher D, Wager E, Middleton P, Altman DG, et al. CONSORT for reporting randomised trials in journal and conference abstracts. *Lancet* 2008, 371:281-283

[2] Hopewell S, Clarke M, Moher D, Wager E, Middleton P, Altman DG at al (2008) CONSORT for reporting randomized controlled trials in journal and conference abstracts: explanation and elaboration. *PLoS Med* 5(1): e20

[3] Ioannidis JP, Evans SJ, Gotzsche PC, O'Neill RT, Altman DG, Schulz K, Moher D. Better reporting of harms in randomized trials: an extension of the CONSORT statement. *Ann Intern Med* 2004; 141(10):781-788.

For peer review only
